# Systems Biology-Driven Discovery of Host-Targeted Therapeutics for Oropouche Virus: Integrating Network Pharmacology, Molecular Docking, and Drug Repurposing

**DOI:** 10.3390/ph18050613

**Published:** 2025-04-23

**Authors:** Pranab Dev Sharma, Abdulrahman Mohammed Alhudhaibi, Abdullah Al Noman, Emad M. Abdallah, Tarek H. Taha, Himanshu Sharma

**Affiliations:** 1 Biotechnology Program, Department of Mathematics and Natural Science, BRAC University, Dhaka 1212, Bangladesh; pranab.dev.sharma@g.bracu.ac.bd; 2 Department of Biology, College of Science, Imam Mohammad Ibn Saud Islamic University (IMSIU), Riyadh 11623, Saudi Arabia; thali@imamu.edu.sa; 3 School of Pharmacy, BRAC University, Dhaka 1212, Bangladesh; abdullah.al.noman@g.bracu.ac.bd; 4 Department of Biology, College of Science, Qassim University, Buraydah 51452, Saudi Arabia; 140208@qu.edu.sa; 5 Teerthanker Mahaveer College of Pharmacy, Teerthanker Mahaveer University, Moradabad 244001, Uttar Pradesh, India; amitsharmaaligarh786@gmail.com

**Keywords:** computational biology, drug development, virus–host protein interaction, neglected tropical diseases, molecular docking validation

## Abstract

**Background:** Oropouche virus (OROV), part of the Peribunyaviridae family, is an emerging pathogen causing Oropouche fever, a febrile illness endemic in South and Central America. Transmitted primarily through midge bites (*Culicoides paraensis*), OROV has no specific antiviral treatment or vaccine. This study aims to identify host-targeted therapeutics against OROV using computational approaches, offering a potential strategy for sustainable antiviral drug discovery. **Methods:** Virus-associated host targets were identified using the OMIM and GeneCards databases. The Enrichr and DSigDB platforms were used for drug prediction, filtering compounds based on Lipinski’s rule for drug likeness. A protein–protein interaction (PPI) network analysis was conducted using the STRING database and Cytoscape 3.10.3 software. Four key host targets—IL10, FASLG, PTPRC, and FCGR3A—were prioritized based on their roles in immune modulation and OROV pathogenesis. Molecular docking simulations were performed using the PyRx software to evaluate the binding affinities of selected small-molecule inhibitors—Acetohexamide, Deptropine, Methotrexate, Retinoic Acid, and 3-Azido-3-deoxythymidine—against the identified targets. **Results:** The PPI network analysis highlighted immune-mediated pathways such as Fc-gamma receptor signaling, cytokine control, and T-cell receptor signaling as critical intervention points. Molecular docking revealed strong binding affinities between the selected compounds and the prioritized targets, suggesting their potential efficacy as host-targeting antiviral candidates. Acetohexamide and Deptropine showed strong binding to multiple targets, indicating broad-spectrum antiviral potential. Further in vitro and in vivo validations are needed to confirm these findings and translate them into clinically relevant treatments. **Conclusions:** This study highlights the potential of using computational approaches to identify host-targeted therapeutics for Oropouche virus (OROV). By targeting key host proteins involved in immune modulation—IL10, FASLG, PTPRC, and FCGR3A—the selected compounds, Acetohexamide and Deptropine, demonstrate strong binding affinities, suggesting their potential as broad-spectrum antiviral candidates. Further experimental validation is needed to confirm their efficacy and potential for clinical application, offering a promising strategy for sustainable antiviral drug discovery.

## 1. Introduction

In an era when climate change and urbanization contribute to the spread of neglected diseases such as arboviruses (arthropod-borne viruses), the Oropouche virus (OROV) has emerged as a significant yet underrecognized threat, causing outbreaks in tropical regions such as Central and South America. Currently, no antivirals or vaccines are available to prevent its transmission. Although no fatalities have been reported, OROV can lead to systemic infections, affecting both the nervous and circulatory systems and potentially resulting in severe complications [1]. The Oropouche virus (OROV) belongs to the order Bunyavirales, family Peribunyaviridae, and genus Orthobunyavirus, classified under the *Orthobunyavirus oropoucheense* species. It is characterized by a negative-sense, single-stranded RNA genome enclosed within a spherical lipid envelope [2,3]. The transmission of the virus occurs principally via the bites of midges (*Culicoides paraensis*) and to a lesser extent by mosquitoes [4]. Epidemics of OROV have been reported in Brazil, Panama, and Peru, and increasing cases are pointing toward broader dissemination [5]. Its clinical features resemble other arboviral diseases including dengue fever, with fever, headache, myalgia, and sometimes neurological complications [6]. Surprisingly, limited information is available regarding the vectors responsible for the ongoing epidemic. Current evidence indicates that, unlike many other arthropod-borne orthobunyaviruses, Oropouche virus (OROV) is primarily transmitted by culicoid midges (Ceratopogonidae: Culicoides), rather than by mosquitoes or ticks [7].

Historically, this virus, OROV, was first identified in Trinidad and Tobago during an outbreak of febrile illness. Since its discovery, epidemics have been documented in various countries across South and Central America, including Brazil, Peru, and Panama [8]. In Brazil alone, the Oropouche virus has been responsible for over 500,000 reported cases, with recent outbreaks linked to fatalities, suspected vertical transmission, and possible associations with microcephaly in newborns. The true burden of Oropouche fever is likely underestimated due to underreporting and inadequate surveillance. Additionally, the limited availability of diagnostic methods, such as serology and RT-PCR, in resource-constrained settings further hampers efforts to monitor and control the virus’s spread [9]. It, however, has no specific antiviral treatment or vaccine despite its epidemiological importance, given that further speculative research on possible suitable therapeutic interventions to target the cellular factors of the host exploited by viruses for replication and survival is quite a promising strategy in antiviral drug discovery. In contrast to direct-acting antivirals, which often face obstacles emerging from viral mutations and resistance, host-targeted therapeutics offer a much wider and perhaps more sustainable approach for the management of viral infections.

For neglected diseases such as Oropouche virus (OROV), which lack targeted therapeutic options, the integration of systems biology and computational drug discovery represents a transformative approach to addressing emerging viral threats [10]. Although the application of these strategies to arboviruses is still in its early stages, recent advances in network pharmacology and multi-omics analyses have uncovered host dependency factors essential for viral replication [11].

Despite the increasing public health threat posed by OROV, significant gaps remain in our understanding of its molecular etiology and in the development of targeted therapies, highlighting a critical global research deficit. Current studies have largely focused on epidemiological surveillance, with limited mechanistic insight into host–virus interactions or immune evasion strategies [12]. Although host-targeted antivirals have shown promise against other arboviruses such as dengue and Zika [13], similar approaches remain largely unexplored for OROV, reflecting a broader trend of systematic neglect toward underprioritized pathogens within global drug development pipelines.

While computational strategies like network pharmacology have proven effective in identifying host-directed therapies for viruses such as SARS-CoV-2 [14], these methods have yet to be applied to OROV. As a result, its druggable host pathways remain uncharted. Furthermore, regional disparities in research funding have hindered the integration of drug repurposing strategies [15], an essential component of pandemic preparedness, into OROV-related investigations.

This study introduces a multi-layered computational framework that integrates network-based target prioritization, robust molecular docking, and drug repurposing—a cost-effective and efficient strategy particularly suitable for neglected diseases—to identify host-directed therapeutics against OROV. By mapping virus–host interactomes and conducting functional enrichment analyses, we elucidate immune-modulatory pathways (e.g., Fcγ receptor signaling, cytokine regulation) hijacked by OROV, thereby identifying mechanistically relevant host targets (IL10, FASLG, PTPRC, FCGR3A) implicated in both viral pathogenesis and immune homeostasis.

Our approach not only prioritizes repurposed drugs with established binding affinities (e.g., Methotrexate, Retinoic Acid), but also mitigates the risk of resistance commonly associated with direct-acting antivirals. Importantly, this work addresses a critical gap in OROV research, which has traditionally focused on epidemiological surveillance rather than mechanistic, host-targeted interventions.

The computational rigor of our method—including the application of Lipinski’s rule-based filters, protein–protein interaction (PPI) network clustering, and Gene Ontology (GO) term enrichment—ensures translational relevance. Furthermore, the identification of druggable immune pathways offers broader implications for combating related arboviruses. By bridging systems biology with antiviral discovery, this study provides a scalable and adaptable framework for rapid therapeutic development, positioning host-targeted strategies as a cornerstone of pandemic preparedness in an era of climate-driven arboviral emergence.

While this study presents a computational approach for host-directed antiviral candidate finding, further in vitro and in vivo validations are needed for confirming efficacy. This study highlights the opportunities present in computational drug discovery to avert the cry of any mathematical interludes ushered by OROV. By coupling network pharmacology with molecular docking, this finds sundry drug suggestions on basis of advanced study in host-targeted therapeutics for the advancement of antiviral weaponry against Oropouche virus infections.

## 2. Results

### 2.1. Targets for Virus

A total of 214 target genes were obtained from the OMIM and Genecard databases after removing 110 duplicate genes. After that, those virus target genes were subjected to mapping into the UniPort database in the condition in Homo sapiens where 7 genes were not mapped and the other 207 targets were accepted for mapping. These 207 mapped genes were used in other steps.

### 2.2. Drug Prediction

**Figure 1:**
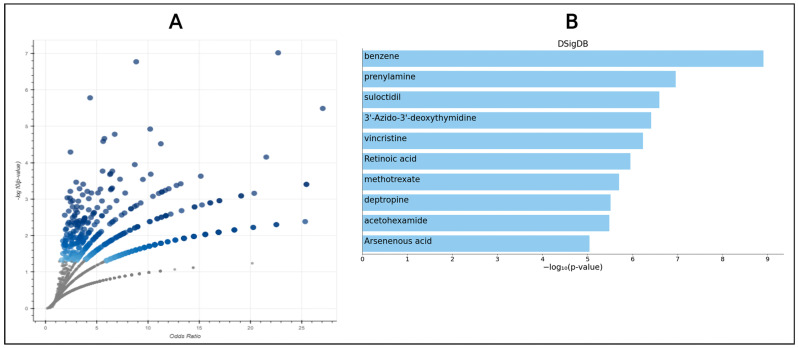
Volcano plot and bar chart for the selected top compounds. (**A**) In the volcano plot, each of the compound is represented by each dot where odds ratio lies along the *x*-axis and −log10(*p*-value) along the y-axis in relation to the enrichment results of the input query gene set. The term is further ordered based on the set enrichment *p*-value for the supplied input gene set, where larger and darker points signify greater terms. Moreover, the colors in this figure highlight significance levels, with darker blue representing highly significant values and lighter shades indicating lower significance. Gray points likely correspond to non-significant data, ensuring contrast in visualization. (**B**) Based on the −log(*p* value), the top 10 enriched terms are displayed for the input gene set. These terms have the highest overlap with the input query gene set. Moreover, the blue-colored bars correspond to terms with significant *p*-values (<0.05).

We used the identified 207 genes for our drug prediction process to see which drugs came out associated with the targets by using the DSigDB database. From that finding, top 10 compounds came out for drug use (Figure 1).

### 2.3. Compound Selection

For known toxicity, compounds should be discontinued from the market as they do not follow the criteria. Five compounds out of ten were removed from further study. The other five molecules were selected, which followed most of the pharmacokinetic criteria (Table 1).

### 2.4. Target Collection for Compounds

According to the different databases, a total of 561 targets were identified for 3-Azido-3-deoxythymidine, 9540 targets for Retinoic Acid, 2691 targets for Methotrexate, 100 for Deptropine, and a total of 115 targets were collected for Acetohexamide. Furthermore, those targets were used to map in the UniProt database by focusing on *Homo sapiens*, where 10,011 targets were mapped and 802 targets were not mapped and removed from the list.

### 2.5. PPI Network Analysis

**Figure 2:**
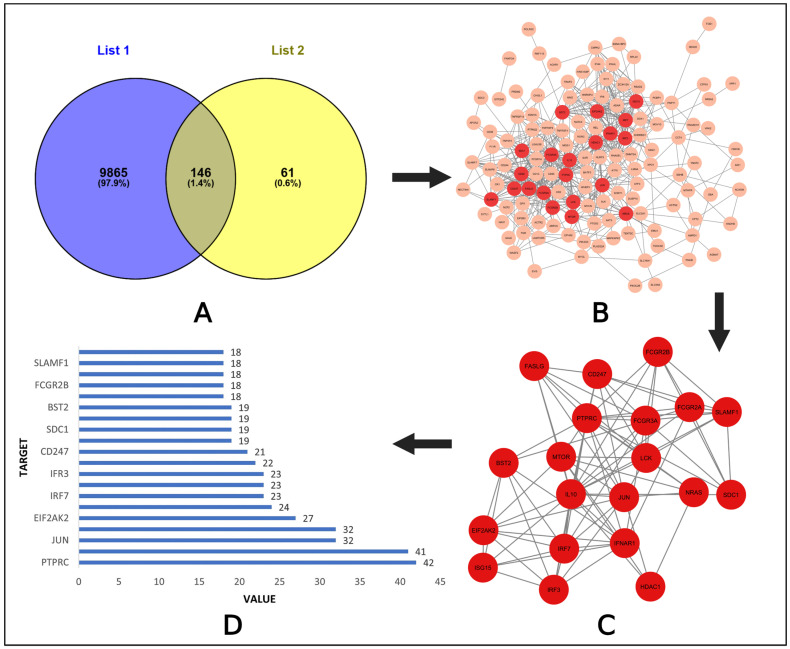
Screening the common targets and their networking interactions. (**A**) Venn diagram showing the intersect targets. (**B**) Protein–protein interaction for common targets. (**C**) Top 20 proteins among the targets. (**D**) List of top proteins with degree value.

From the previous analysis, 207 target genes were collected for the virus and 10,011 targets for potential drugs. By utilizing the Venny tool, we found out the common targets for both specific viruses and compounds. The graph shows a total of 146 common targets (Supplementary Table S1, Figure 2A).

The intersect genes were subjected to an insert into string for constructing a PPI network that had 133 nodes and 597 edges (Figure 2B). We focused on the degree value because a higher degree value means more interactions in the network and shows more importance. We calculated the top 20 hub genes based on the degree value (Figure 2C). The degree value for each top node is shown in Figure 2D. PTPRC, IL10, JUN, FCGR3A, EIF2AK2, LCK, IRF7, IRF3, FCGR2A, IFNAR, CD247, MTOR, SDC1, HDAC1, BST2, NRAS, FCGR2B, FASLG, SLAMF1, and ISG15 were the identified key node proteins.

### 2.6. MCODE Analysis

**Figure 3:**
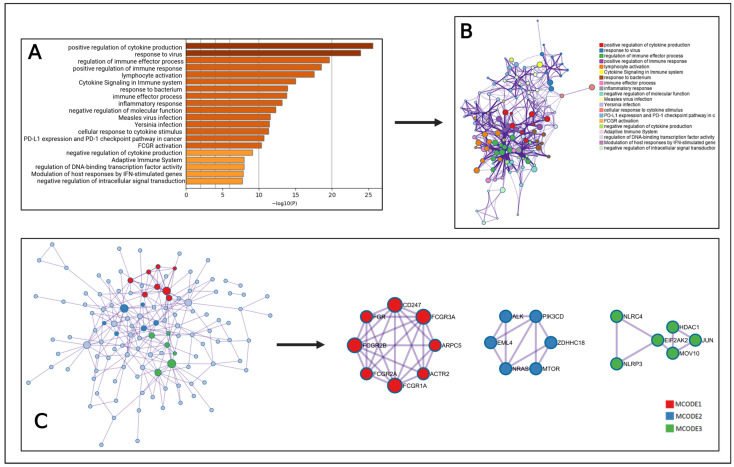
Cluster module analysis diagram. (**A**) Highly enriched terms. The colors in the bars represent the significance of different biological processes, pathways, or functions. Darker shades indicate higher significance levels based on the −log10(*p*) value, while lighter shades represent lower significance. This helps visually distinguish the most statistically relevant terms in the dataset. (**B**) Sub-network formation on specific interaction. (**C**) Different cluster analysis.

To identify core therapeutic targets, the MCODE algorithm was applied, by which we constructed a modular network. Different key functions, such as positive regulation of cytokine production, response to virus, regulation of immune effector process, and positive regulation in immune response, were identified from the topological analysis in Metascape (Figure 3A). A subset network was generated to understand the relationship between pathways and targets (Figure 3B). This network helps us to find out different important functions of targets within different clusters. Moreover, Metascape performed MCODE cluster analysis of the 146 intersect genes. That analysis provided three target modules for treating Oropouche virus by using the selected compounds. These modules help to obtain the biological pathways that might be present in therapeutic impacts on the virus infection.

MCODE 1 is enriched with three different pathway and eight genes, including FGR, CD247, FCGR3A, ARPC5, ACTR2, FCGR1A, FCGR2A, and FCGR2B (Figure 3C, Table 2). Additionally, MCODE 2 and MCODE 3 both showed three pathways, where EML4, NRAS, MTOR, ZDHHC18, PIK3CD, and ALK are the genes present in MCODE 2 and NLRP3, NLRC4, EIF2AK2, HDAC1, JUN, and MOV10 are for MCODE 3 (Figure 3C, Table 2).

### 2.7. Gene Ontology and KEGG Pathway Analysis

**Figure 4:**
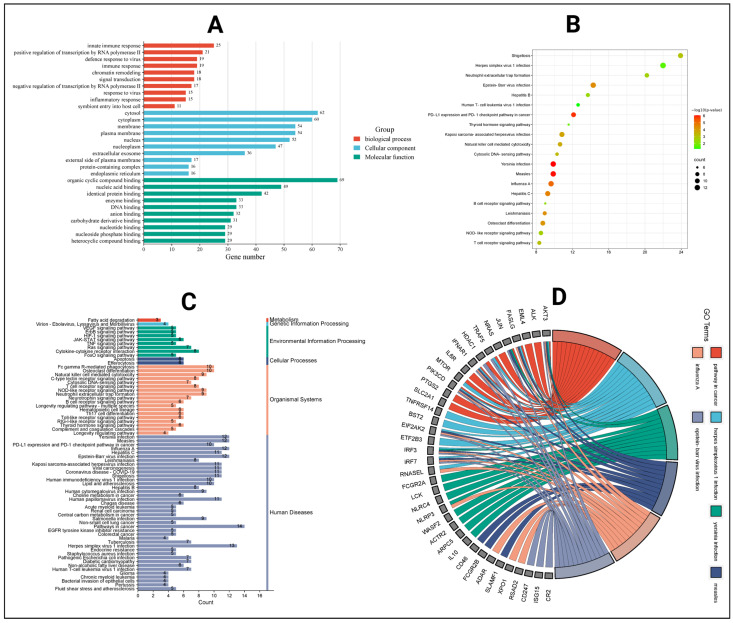
Gene ontology and KEGG pathway analysis. (**A**) Different gene ontology functions in bar chart. (**B**) KEGG enrichment in bubble plot. (**C**) Different systems and actions of KEGG pathways. (**D**) Pathway network for KEGG pathways.

To perform GO functional and KEGG pathway enrichment analysis, the 146 intersecting genes were analyzed in the DAVID online platform. The analysis displayed a total of 312 entries, where 162 were involved in biological process (BP) (Supplementary Table S2), 31 entries for cellular component (CC) (Supplementary Table S3), 49 for molecular function (MF) (Supplementary Table S4), and 70 results for KEGG pathway (Supplementary Table S5). Innate immune response, positive regulation of transcription by RNA polymerase II, defense response to virus, immune response, and chromatin remodeling were present in biological process. The CC terms displayed cytosol, cytoplasm, membrane, plasma membrane, and nucleus, while the MF terms included organic cyclic compound binding, nucleic acid binding, identical protein binding, enzyme binding, and DNA binding. Top 10 entries based on the gene count value are shown in the enrichment bar chart (Figure 4A).

**Figure 5:**
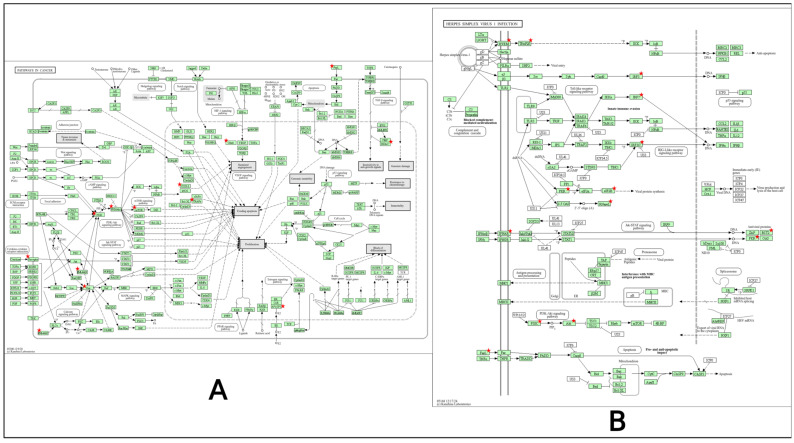
Top 2 pathways based on the gene count value. Red stars indicate key regulatory nodes within the pathway, highlighting critical molecular interactions associated with cancer progression and Herpes simplex virus 1 infection. These elements represent significant genes or proteins involved in cellular signaling and disease mechanisms. (**A**) Pathway in cancer. (**B**) Herpes simplex virus 1 infection.

A total of 70 pathways were identified by the KEGG pathway analysis, where the pathway in cancer and the herpes simplex virus 1 infection pathway had the highest gene value (Figure 5). Another 20 pathways are presented in the bubble plots selected by *p*-value (Figure 4B). Furthermore, all the 70 pathways of KEGG data were mapped based on actions and functions, which selected 1 pathway in metabolism, 1 in genetic information process, 8 pathways in environmental information, 2 are present in cellular process, 18 in organism systems, and human disease displayed 40 pathways (Figure 4C). Moreover, with the highest amount of genes the top six pathways are presented in the chord diagram (Figure 4D).

### 2.8. Target to Pathway Network

**Figure 6:**
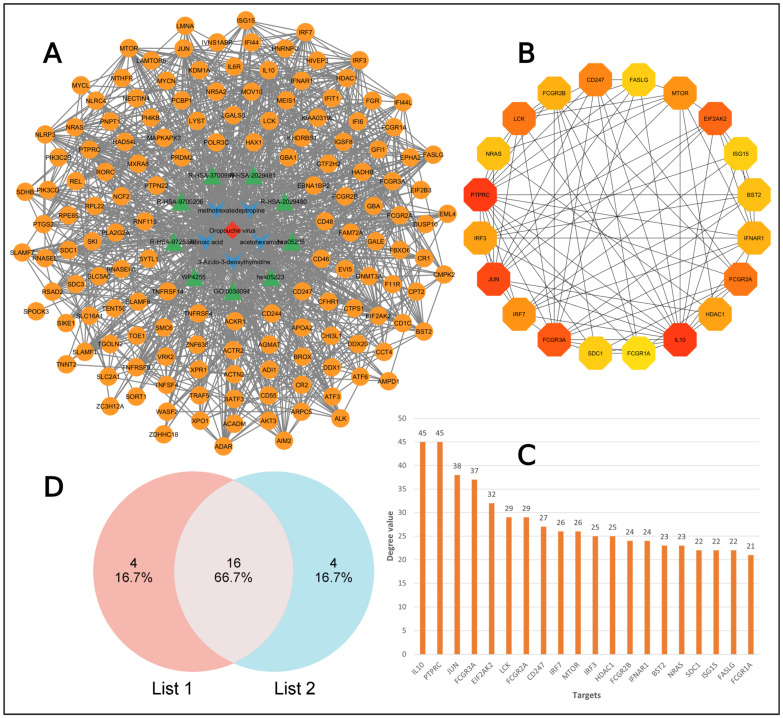
Visualization of target–pathway network interaction. (**A**) Virus–drug–pathway–target network diagram. (**B**) Network of top 20 targets. (**C**) Bar chart for top 20 genes with the degree value. (**D**) Core target identification between PPI network (list 1) and target–pathway network (list 2).

Using the Cytoscape 3.10.3 software, we constructed a target to pathway network involving 162 nodes and 1018 edges (Figure 6A). This network shows interaction among virus, targets, drug, and pathways. The red diamond shape represents the virus, green triangle shapes are the pathways that were identified in MCODE clustering, V-shapes indicates drug components, and the orange-colored circles are the targets.

After that, a network was constructed with the top 20 targets based on their degree value by using the cytohubba plugin in Cytoscape (Figure 6B), and their degree values are presented in the bar chart (Figure 6C). The top 20 targets from protein–protein interaction and top 20 targets from target–pathway network interaction was used to intersect to obtain the common targets and were visualized with a Venn diagram (Figure 6D). Finally, PTPRC, IL10, JUN, FCGR3A, EIF2AK2, LCK, IRF7, CD247, MTOR, SDC1, HDAC1, BST2, NRAS, FCGR2B, FASLG, and ISG15 were identified as core targets.

### 2.9. Key Target Identification

**Figure 7:**
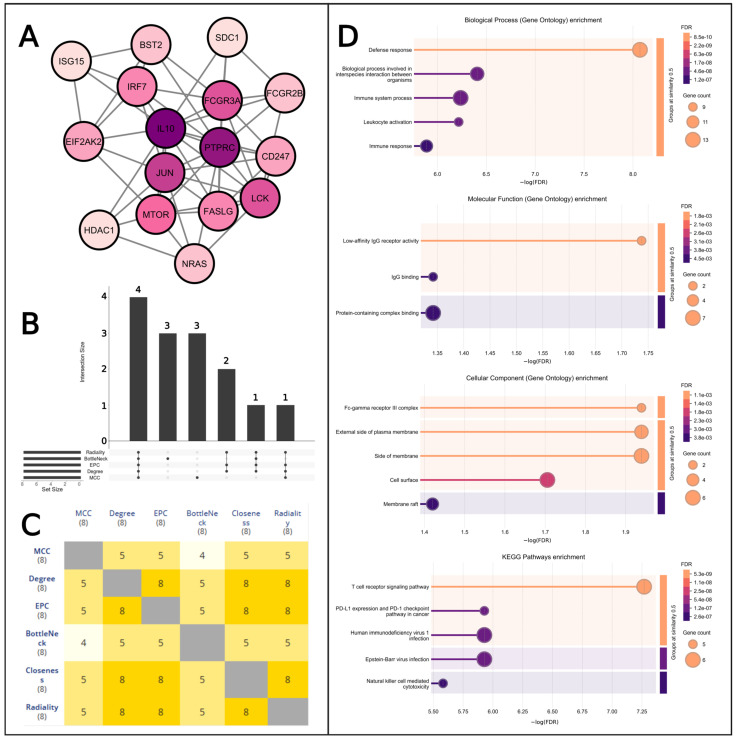
Identification of common targets for different properties and their pathways. (**A**) Network construction between the hub genes. Here, the color gradient likely reflects variations in degree centrality, with darker shades indicating nodes with higher connectivity and lighter shades representing lower-degree values. (**B**) Determining common targets based on topological properties. (**C**) Heat map for showing interaction among two properties. (**D**) Enrichment analysis for the regulatory network. The color gradient in the bars represents False Discovery Rate (FDR) values, providing a visual cue for statistical significance—warmer tones (orange to pink) indicate higher confidence, while cooler shades (purple) reflect decreasing significance. The orange color marks a strong statistical threshold, ensuring data reliability with minimal false positives while acting as a midpoint between highly significant and slightly higher FDR values. This strategic color choice enhances clarity, making robust findings stand out while maintaining a smooth visual transition across the significance scale.

The PPI network from the String database was use in the Cytoscape software to obtain top ten genes for the five different topological properties (Maximal Clique Centrality (MCC), Closeness, BottleNeck, Edge Percolated Component (EPC), Degree value, and Radiality). We created a simple network of those 16 targets to visualize their interactions (Figure 7A). The upset plot shows four targets that are common for all six topological properties in the first bar (Supplementary Table S6, Figure 7B). The first bar represents FASLG, FCGR3A, IL10, and PTPRC as common targets for all the selected properties. Also, the heat map in Figure 7C shows the common genes between two properties. Moreover, we checked different Gene Ontology properties for the top 16 targets. Top five terms for biological processes (BP), cellular component (CC), molecular function (MF) (only has three terms), and KEGG pathways of those genes are visualized in Figure 7D. Defense response, low-affinity lgG receptor activity, Fc-gamma receptor III complex, and T-cell receptor signaling pathway some are the necessary pathways for the 16 hub genes.

### 2.10. Docking Result

By yielding the binding energies among the compounds (small molecules) and proteins (large molecules), the docking interactions among the identified compounds and proteins were validated. The lower the binding affinity, the stronger the interaction between a compound and protein. The binding results of the targets and compounds are summarized in Table 3 and all the affinities were less than −5 kcal/mol.

**Figure 8:**
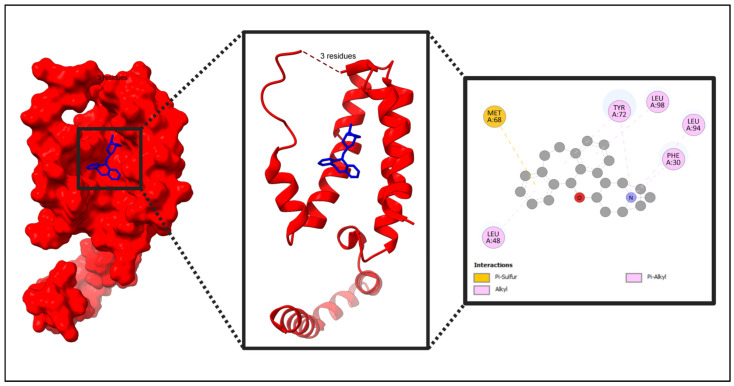
Molecular interaction of IL10 with the selected compound (IL10 + Deptropine). In this figure, red color indicates the protein and blue color indicates the compound. Additional data related to Figure 8 can be found in Supplementary Figures S1–S4.

**Figure 9:**
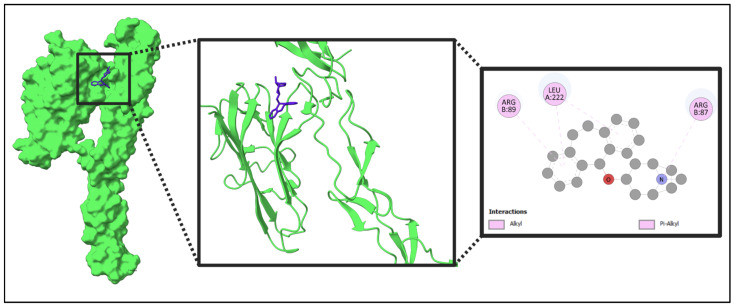
Molecular interaction of FASLG with the selected compound (FASLG + Deptropine). In this figure, green color indicated the protein and blue color indicated the compound. Additional data related to Figure 9 can be found in Supplementary Figures S5–S8.

**Figure 10:**
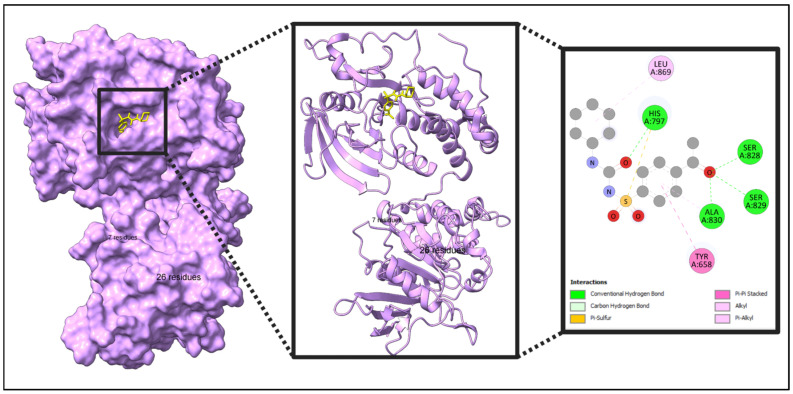
Molecular interaction of PTPRC with the selected compound (PTPRC + Acetohexamide). In this figure, violate color indicates the protein and yellow color indicates the compound. Additional data related to Figure 10 can be found in Supplementary Figures S9–S12.

**Figure 11:**
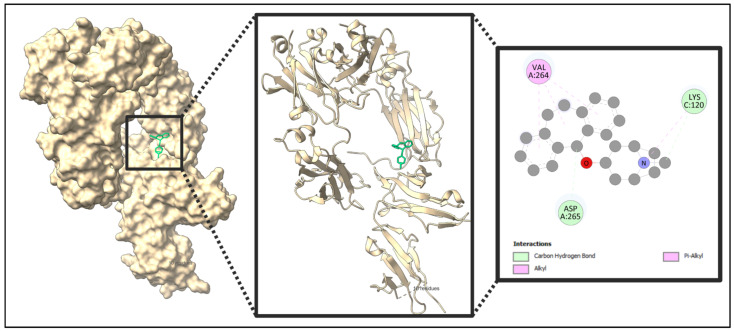
Molecular interaction of FCGR3A with the selected compound (FCGR3A + Deptropine). In this figure, beige color indicates the protein and green color indicates the compound. Additional data related to Figure 10 can be found in Supplementary Figures S13–S16.

The five identified compounds were selected to determine the combinations with four core targets. The visualized results are presented in Figure 8, Figure 9, Figure 10 and Figure 11. Usually, a binding value lower than −7 kcal/mol means a strong predicted binding, and a value lower than −5 kcal/mol suggests moderate binding [16].

**Figure 12:**
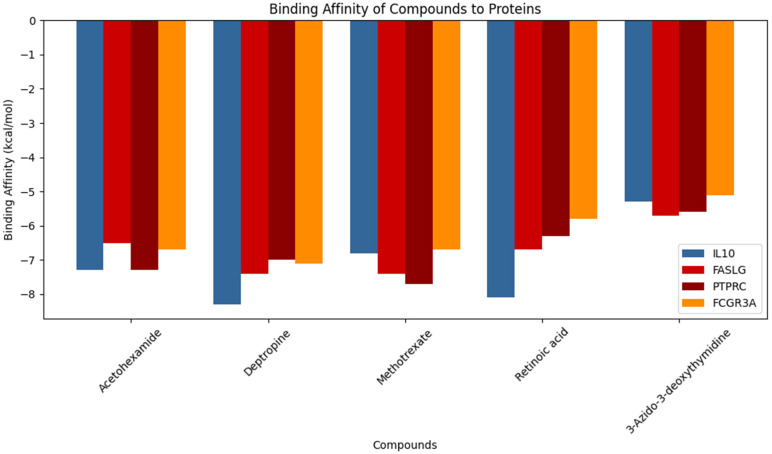
Binding affinity comparison.

The molecular binding interactions from PyRx reveals that Deptropine demonstrates a superior binding affinity across all proteins, particularly to IL10 (−8.3 kcal/mol), significantly outperforming 3-azido-3-deoxythymidine, which consistently exhibited the weakest binding (−5.3 kcal/mol) (Table 3). Acetohexamide and Methotrexate display comparable binding profiles with a strong affinity to IL10 and PTPRC, though Methotrexate binds slightly more strongly to PTPRC (−7.3 vs. −7.7 kcal/mol) (Table 3). Retinoic Acid shows a strong binding to IL10 (−8.1 kcal/mol), with a more gradual decrease across other proteins compared to the steeper drops observed with other compounds. Among proteins, IL10 generally exhibits the strongest compound interactions (except with Acetohexamide where PTPRC binding is stronger), followed by PTPRC, then FASLG with intermediate affinity, while FCGR3A demonstrates a relatively weaker binding (Table 3). The overall binding patterns suggest that aromatic compounds (Deptropine, Methotrexate) and larger molecules bind more strongly, while IL10’s consistent strong binding across compounds indicates it may possess a more accessible binding pocket or favorable electrostatic environment (Figure 12, Table 3).

In this paper, IL10 with Acetohexamide, Deptropine, and Retinoic Acid; FASLG with Deptropine and Methotrexate; PTPRC with Acetohexamide and Methotrexate; and FCGR3A with Deptropine exhibited the strongest binding affinities according to the results from PyRx (Table 3).

**Figure 13:**
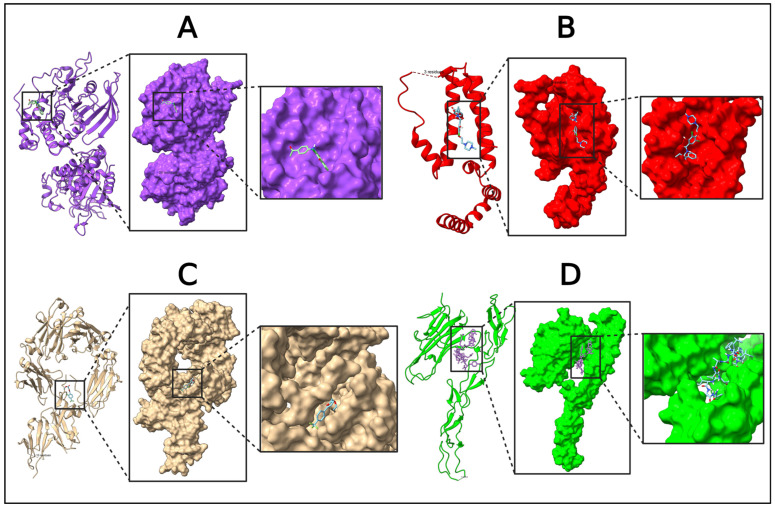
Molecular interactions of target proteins with their know inhibitors. (**A**) PTPRC (violet color) + 2-(4-Acetylanilino)-3-chloronaphthoquinone (sky blue with red), (**B**) IL10 (red color) + JTE-607 (sky blue with red), (**C**) FCGR3A (beige color) + BI-1206 (sky blue with red), and (**D**) FASLG (Green color) + ONL1204 (sky blue with red).

The molecular docking analysis comparing the selected compounds with known inhibitors revealed promising findings based on PyRx (Table 3 and Table 4, Figure 8, Figure 9, Figure 10, Figure 11 and Figure 13). Deptropine exhibited a stronger binding affinity (Table 3, Table 4) than the reference inhibitor JTE-607 (Table 3) for IL10 (−8.3 kcal/mol vs. −6.7 kcal/mol), suggesting a potentially enhanced interaction. Similarly, Methotrexate demonstrated better binding with PTPRC compared to its known inhibitor 2-(4-Acetylanilino)-3-chloronaphthoquinone (−7.7 kcal/mol vs. −7.3 kcal/mol). Additionally, Deptropine showed a higher binding affinity to FCGR3A than BI-1206 (−7.1 kcal/mol vs. −6.5 kcal/mol). However, for FASLG, the known inhibitor ONL1204 exhibited a stronger affinity (−7.7 kcal/mol) than the best-performing selected compounds Methotrexate and Deptropine (−7.4 kcal/mol each) (Table 3 and Table 4). Overall, these results suggest that Deptropine and Methotrexate are promising candidates for IL10, PTPRC, and FCGR3A.

The binding affinity results from the two docking tools (PyRx and SwissDock) reveal both alignments and notable variations across different compounds and proteins (Table 3). While the general trends remain consistent, specific differences highlight methodological nuances between the tools.

For Acetohexamide (CID: 1989), both tools predicted similar binding affinities for IL10 (−7.3 kcal/mol), indicating consistency in the docking results. However, SwissDock predicted a stronger binding for FASLG (−6.8 kcal/mol vs. −6.5 kcal/mol) and FCGR3A (−7.0 kcal/mol vs. −6.7 kcal/mol), with the difference ranging from 0.3 to 0.5 kcal/mol. Conversely, the first tool (PyRx) predicted a stronger binding for PTPRC (−7.3 kcal/mol vs. −7.1 kcal/mol), though the variation remains minor at 0.2 kcal/mol (Table 3).

Deptropine (CID: 203911) shows notable differences, where the first tool consistently predicted stronger binding affinities across all proteins. The largest discrepancy occurred for IL10 (−8.3 kcal/mol vs. −7.0 kcal/mol), showing a difference of 1.3 kcal/mol, while FASLG (−7.4 kcal/mol vs. −6.9 kcal/mol), PTPRC (−7.0 kcal/mol vs. −6.4 kcal/mol), and FCGR3A (−7.1 kcal/mol vs. −6.8 kcal/mol) exhibited differences ranging from 0.3 to 0.6 kcal/mol. This variation suggests that SwissDock may apply different scoring functions or ligand flexibility considerations (Table 3).

Methotrexate (CID: 126941) presented mixed results, with SwissDock predicting a stronger binding for IL10 (−7.8 kcal/mol vs. −6.8 kcal/mol) and FCGR3A (−8.1 kcal/mol vs. −6.7 kcal/mol), showing substantial differences of 1.0 and 1.4 kcal/mol, respectively. Meanwhile, the binding affinities for FASLG remained identical at −7.4 kcal/mol across both tools. The first tool predicted a stronger binding for PTPRC (−7.7 kcal/mol vs. −7.4 kcal/mol), but the difference is smaller at 0.3 kcal/mol (Table 3).

Retinoic Acid (CID: 449171) generally shows a stronger binding in SwissDock, with notable differences for FASLG (−7.1 kcal/mol vs. −6.7 kcal/mol), PTPRC (−7.1 kcal/mol vs. −6.3 kcal/mol), and FCGR3A (−6.9 kcal/mol vs. −5.8 kcal/mol). The largest deviation occurred in FCGR3A at 1.1 kcal/mol, suggesting SwissDock models binding interactions differently, possibly considering additional hydrophobic or electrostatic contributions (Table 3).

Finally, 3-Azido-3-deoxythymidine (CID: 5726) demonstrated the most substantial deviation across all proteins, where SwissDock consistently predicted a stronger binding affinity. The differences ranged from 1.1 to 1.7 kcal/mol, with SwissDock showing a consistently stronger binding.

## 3. Discussion

This study employed a systematic computational approach to identify host-targeted therapeutics for Oropouche virus (OROV) by integrating network pharmacology, molecular docking, and drug repurposing. The findings suggest that targeting host factors could be an effective antiviral strategy, reducing the risk of resistance that often arises with direct-acting antivirals. Our study identified IL10, FASLG, PTPRC, and FCGR3A as key immune-related host proteins involved in OROV infection. Small-molecule inhibitors Acetohexamide, Deptropine, Methotrexate, Retinoic Acid, and 3-Azido-3-deoxythymidine exhibited strong binding affinities with these targets, highlighting their potential for repurposing as antiviral agents.

Our findings align with recent studies on host-directed antiviral strategies for arboviruses, including dengue virus (DENV), Zika virus (ZIKV), and Chikungunya virus (CHIKV) [17,18]. Prior research has demonstrated that targeting immune regulators such as IL10 can modulate viral infection outcomes. A study on DENV suggested that IL10 dysregulation aids viral immune evasion, and blocking IL10 pathways enhances viral clearance [19,20]. Similarly, PTPRC (CD45) has been implicated in viral pathogenesis, particularly in HIV and Epstein–Barr virus (EBV), where T-cell activation plays a crucial role in disease progression [21]. The identification of these immune checkpoints in OROV infection suggests that the virus may employ immune modulation mechanisms similar to other viral pathogens, reinforcing the viability of these proteins as therapeutic targets [22,23].

Our protein–protein interaction (PPI) analysis revealed the clustering of immune-related pathways, including Fc-gamma receptor signaling, cytokine regulation, and T-cell receptor signaling. These pathways have also been reported as crucial intervention points in other arboviral infections, where Fc-gamma receptor signaling modulates viral antibody-dependent enhancement (ADE) [24]. Studies have shown that flaviviruses like ZIKV and DENV exploit Fc-mediated pathways to enhance viral infection, emphasizing the potential role of FCGR3A in viral pathogenesis [25,26]. Our analysis extends these findings to OROV, identifying FCGR3A (CD16a) as a potential antiviral target, aligning with prior work on immune modulation in viral infections [27].

For a proper molecular docking, the active site of each protein was identified using Discovery Studio [28], a widely recognized computational tool known for its robust structural analysis capabilities [29,30,31]. Unlike online tools that merely confirm the presence of an active site, Discovery Studio allows a more refined approach, enabling precise characterization based on ligand interactions, binding pocket geometry, and energetic considerations [32,33]. This level of structural validation strengthens the reliability of the identified active sites, ensuring consistency with established biochemical principles.

The molecular docking analysis demonstrated that Deptropine and Acetohexamide exhibited the strongest binding affinities across multiple targets, suggesting their broad-spectrum antiviral potential. Similar computational approaches have identified repurposable drugs against other viruses, where docking studies highlighted the importance of drug binding to host immune regulators. Notably, our binding affinities for Deptropine (−8.3 kcal/mol with IL10) and Acetohexamide (−7.3 kcal/mol with PTPRC) are comparable to findings from previous antiviral docking studies, where binding affinities below −7 kcal/mol were associated with strong target engagement [16]. These results suggest that the identified compounds could serve as promising candidates for further investigation in antiviral drug discovery, notably, Retinoic Acid, known for its role in immune modulation [34]. Among them, Deptropine and Acetohexamide exhibited strong binding affinities to multiple targets, suggesting broad-spectrum antiviral potential. They have demonstrated antiviral properties against other RNA viruses [35]. Methotrexate, primarily used as an immunosuppressant [36], may influence viral replication through the indirect regulation of host immune responses [37].

The molecular docking results provide insights into the binding potential of the selected compounds compared to known inhibitors. Deptropine exhibited a stronger binding affinity than JTE-607 for IL10 (−8.3 kcal/mol vs. −6.7 kcal/mol), indicating a potentially enhanced interaction. Similarly, Methotrexate demonstrated improved binding to PTPRC compared to its known inhibitor 2-(4-Acetylanilino)-3-chloronaphthoquinone (−7.7 kcal/mol vs. −7.3 kcal/mol). Additionally, Deptropine showed a better binding affinity for FCGR3A than BI-1206 (−7.1 kcal/mol vs. −6.5 kcal/mol), suggesting that it may be a promising candidate for targeting this protein.

The binding affinities predicted by PyRx and SwissDock exhibited both concordance and divergence, underscoring the importance of multi-tool validation in computational drug discovery. While there was consistency with general trends (e.g., Methotrexate’s strong binding to PTPRC), notable discrepancies emerged, such as Deptropine’s IL10 affinity (−8.3 kcal/mol in PyRx vs. −7.0 kcal/mol in SwissDock). These variations likely stem from methodological differences: PyRx’s AutoDock Vina employs a gradient-optimized scoring function, whereas SwissDock’s EADock DSS incorporates solvation effects and broader conformational sampling. The stronger FCGR3A binding predicted by SwissDock for Methotrexate (−8.1 kcal/mol vs. −6.7 kcal/mol in PyRx) suggests its algorithm may better capture hydrophobic interactions, a critical factor for this receptor. Based on integrated docking results from PyRx and SwissDock, Deptropine emerges as the most promising candidate against OROV. It showed the strongest binding to IL10 (−8.3 kcal/mol in PyRx, −7.0 kcal/mol in SwissDock), outperforming the known inhibitor JTE-607 (−6.7 kcal/mol). Deptropine also exhibited robust affinity for FCGR3A (−7.1 kcal/mol in PyRx, −6.8 kcal/mol in SwissDock), surpassing BI-1206 (−6.5 kcal/mol). While Methotrexate bound well to PTPRC (−7.7 kcal/mol), its weaker interaction with IL10 (−6.8 kcal/mol) limits its broad-spectrum potential. Acetohexamide showed moderate binding (−6.5 to −7.3 kcal/mol), while Retinoic Acid and 3-Azido-3-deoxythymidine had consistently weaker affinities (> −7 kcal/mol). Deptropine’s multi-target inhibition of IL10, FCGR3A, and FASLG suggests it could disrupt immune evasion, viral entry, and apoptosis pathways simultaneously.

IL10, a key target in our study, plays a dual role in viral infections. While it modulates inflammatory responses to prevent excessive tissue damage, it can also suppress antiviral immunity, enabling viral persistence [20,27]. The strong docking interactions of Acetohexamide, Deptropine, and Retinoic Acid with IL10 suggest that these drugs could modulate IL10 signaling to enhance antiviral immunity, similar to IL10-targeting strategies explored in HCV and DENV research [19]. Additionally, FASLG (Fas Ligand) is crucial for apoptosis regulation, and many viruses, including DENV and HIV, manipulate apoptotic pathways to avoid immune clearance [38]. Our docking results show that Methotrexate and Deptropine bind strongly to FASLG, suggesting a potential mechanism to restore apoptosis and eliminate infected cells. Furthermore, PTPRC (CD45) is essential for T-cell receptor signaling and immune activation, and its involvement in OROV infection suggests a novel immune evasion strategy by the virus [21]. Our findings align with previous studies on HIV and EBV, where CD45-targeting drugs altered disease progression [21]. Finally, FCGR3A plays an essential role in antibody-dependent cellular cytotoxicity, a key mechanism in viral clearance [27,39]. Though its role in OROV infection is not well-documented, its involvement in other viral infections suggests that targeting this pathway may enhance antiviral immunity [27].

While literature-reported binding sites offer valuable reference points, protein–ligand interactions are inherently dynamic and context-dependent. The observed binding of the proposed compounds to alternative residues suggests potential mechanistic diversity rather than deviation from established findings [40,41,42]. This variability does not diminish the relevance of the compounds; rather, it broadens the scope for further experimental validation [43,44,45]. Such insights can pave the way for discovering novel therapeutic interactions, emphasizing the importance of considering both canonical and alternative binding modes in drug discovery [46,47,48].

Despite the promising findings, our study has several limitations. First, while computational approaches provide valuable insights, they lack experimental validation. Our aim of this study is to systematically integrate host–virus interaction networks with drug prediction with docking validation. Similar approaches have been employed for other arboviruses, such as dengue and chikungunya, where host-targeting strategies have yielded promising antiviral candidates [17,49]. Future studies should include in vitro antiviral assays to confirm the efficacy of identified drug candidates. Additionally, molecular dynamics (MD) simulations should be performed to assess the stability of drug–protein interactions beyond docking. Second, the study primarily focuses on network pharmacology and docking but lacks a full multi-omics approach. Integrating transcriptomics and proteomics data could enhance target validation. Finally, future work should involve in vitro studies in human cell lines and animal models to evaluate the antiviral efficacy of Acetohexamide, Deptropine, and Methotrexate.

In conclusion, this study presents a computational framework for identifying host-targeted antiviral candidates against Oropouche virus. By targeting immune-modulating proteins such as IL10, FASLG, PTPRC, and FCGR3A, we propose a novel antiviral strategy that aligns with recent host-directed antiviral research. However, experimental validation remains critical, and future studies should explore in vitro/in vivo assays to confirm the therapeutic potential of the identified compounds. Integrating multi-omics data and molecular dynamics simulations will further strengthen the translational value of these findings in antiviral drug discovery.

## 4. Materials and Methods

### 4.1. Target Identification for Oropouche Virus

The targets associated with the Oropouche virus were collected from the OMIM (Online Mendelian Inheritance in Man) database (https://www.omim.org/, accessed on 6 March 2025) and GeneCards database (https://www.genecards.org/, accessed on 6 March 2025) [50,51]. The keywords “Oropouche virus” and “Oropouche fever” were set as search options in those databases. Related target symbols corresponding to the Oropouche virus were accumulated.

### 4.2. Identified Drugs

Within the Enrichr platform (https://maayanlab.cloud/Enrichr/, accessed on 6 March 2025), the DSigDB database (https://dsigdb.tanlab.org/DSigDBv1.0/, accessed on 6 March 2025) was applied to detect the drug molecules that are connected to the target genes for the virus [52]. The filtering criterion was adjusted in *p* < 0.05. From that analysis, the top 10 compounds were identified as the potential applicants for inhibiting the Oropouche virus [53].

### 4.3. Selected Compounds

We followed Lipinski’s rule parameters for drug criteria such as molecular weight (<500 g/mol), hydrogen bond acceptors (<10), hydrogen bond donors (≤5), topological polar surface area (<150), and Moriguchi octanol–water partition coefficient (≤4.15). To calculate those criteria, the SwissADME tool was used [54].

### 4.4. Target Gene Identification for Selected Compounds

Different databases were utilized to collect the target genes for each selected compound. The name of the compounds was used as a keyword for searching. For obtaining the target genes, we used CTD (Comparative Toxicogenomics Database) (https://ctdbase.org/, accessed on 6 March 2025), Swiss Target Prediction tool (http://www.swisstargetprediction.ch/, accessed on 6 March 2025), Drug Bank (https://go.drugbank.com/, accessed on 25 February 2025), and SymMap (http://www.symmap.org/, accessed on 6 March 2025) [55,56]. Then, the UniPort database was utilized to cross-reference the detected targets for *Homo sapiens* [57].

### 4.5. Intersection of Targets

To determine the common targets for the virus and compounds, the Venny 2.0 tool (https://bioinfogp.cnb.csic.es/tools/venny/index2.0.2.html, accessed on 6 March 2025) was used. For further analysis, those common targets were applied, and these are the connective targets between considered compounds as drugs and the virus [58].

### 4.6. Protein–Protein Interaction Network

The String database (https://string-db.org/, accessed on 6 March 2025) was applied to create the PPI network among those intersected targets and specifying “Homo sapiens” as the species [59]. The interaction file then used to visualize them in the Cytoscape 3.10.3 software [59]. After that, the top twenty common targets were identified by the CytoHubba plugin of Cytoscape [60].

### 4.7. Analysis by MCODE

For creating functional modules and clustering similar proteins, we used MetaScape (https://metascape.org/, accessed on 6 March 2025) with molecular complex detection (MCODE) [61]. With the aid of a custom MCODE tool, a clustering analysis was performed for common targets where a minimum overlap of 3, a *p*-value cutoff of 0.05, and a minimum enrichment of 1.5 were used. Using MCODE analysis, protein relationship complexes were identified and organized into gene clusters, where each target can be associated with particular gene functions. Moreover, other regulatory transcription factors that modulated the specified targets were also predicted to inform the user of possible target interactions.

### 4.8. Gene Ontology Analysis

By using the DAVID online platform (https://davidbioinformatics.nih.gov/, accessed on 6 March 2025), Gene Ontology (GO) and Kyoto Encyclopedia of Genes and Genomics (KEGG) pathways were analyzed for the intersection targets of the virus and compounds where biological processes (BPs), cellular components (CCs), molecular functions (MFs), and KEGG pathways were included [62]. In this analysis, the top ten GO entries and seventy KEGG pathways were identified based on gene count value. The bioinformatics online platforms (https://www.bioinformatics.com.cn/en, accessed on 6 March 2025) were used for visualization. Furthermore, the overlapping genes underwent KEGG mapping analysis by focusing on Homo sapiens. Metabolism, environmental information processing, cellular processes, systems of the organism, and human diseases are the pathways that were included in that analysis.

### 4.9. Compound–Pathway–Target Interaction Network

From the Metascape analysis, the complex biological pathways were identified by using MCODE cluster calculations. Those complex biological pathways were used to construct a compound–pathway–target interaction network by using the intersecting genes and compounds.

### 4.10. Identified Key Targets

The identified final targets were used to compare them to find out key targets. For further analysis, those final targets were used in the STRING (https://string-db.org/, accessed on 6 March 2025) online platform to create a network among those genes. The required score was set to 0.700 to obtain a high confidence in network construction, and the species was set as Homo sapiens. After that, the Cytoscape 3.10.3 software along with the Cytohubba plugin were utilized to visualize and analyze the network. To find out the key targets, five different topological properties (Maximal Clique Centrality (MCC), Closeness, BottleNeck, Edge Percolated Component (EPC), Degree value, and Radiality) were set. The UpSetR Shinny App (https://gehlenborglab.shinyapps.io/upsetr/, accessed on 6 March 2025) was utilized to see the number of common genes for those different topological factors [63]. We also applied molbiotools (https://molbiotools.com/listcompare.php, accessed on 6 March 2025) for comparing all six properties.

### 4.11. Use of Positive Control Inhibitors

#### 4.11.1. Using Positive Controls

To ensure the reliability and accuracy of our molecular docking simulations, we included positive control inhibitors for each target protein. Positive controls are known compounds that are expected to bind to the target proteins and provide a benchmark for validating the docking methodology [64]. By docking these inhibitors alongside the target proteins, it is helpful to assess the docking protocol’s ability to correctly predict protein–ligand interactions, even in the absence of literature-reported binding affinities [65,66].

#### 4.11.2. Selection of Positive Control Inhibitors

For each of the target proteins (FASLG, FCGR3A, IL10, and PTPRC), the following positive control inhibitors were selected based on their known interactions or theoretical binding to these proteins:

- FASLG (Fas Ligand): One promising inhibitor for FASLG is ONL1204, a small peptide antagonist of the Fas receptor. It has demonstrated neuroprotective effects in models of glaucoma, reducing neuroinflammation and preventing axon degeneration [67].
- FCGR3A (Fc gamma receptor IIIa): The inhibitor BI-1206 was selected for FCGR3A based on its a monoclonal antibody that blocks Fc gamma receptor IIIa to enhance immune responses in cancer therapy [68].
- IL10 (Interleukin 10): A well-characterized positive control inhibitor for IL10 (Interleukin-10) is JTE-607, which selectively inhibits inflammatory cytokine synthesis, including IL10 [69].
- PTPRC (Protein Tyrosine Phosphatase Receptor Type C): A well-characterized positive control inhibitor for PTPRC (CD45) is CD45 Inhibitor VI, also known as 2-(4-Acetylanilino)-3-chloronaphthoquinone. It is a chloronaphthoquinone compound that potently inhibits PTPRC tyrosine phosphatase activity in a non-substrate-competitive and irreversible manner [70].

### 4.12. Molecular Docking

#### 4.12.1. Protein and Compound Preparation

First of all, each protein was added into the Discovery Studio software for removing heteroatoms such as water and other ligands. After that, those proteins were inserted into the Chimera X 1.19 software for further preparation [71]. In this process, hydrogens were added, charges and missing atoms were added, and the prepared proteins were saved as a PDB file for docking [72]. For docking, we used the PyRx software, which has the accessibility of Autodock Vina. Each protein was added there and converted into a macromolecule, and the next grid box for each protein was adjusted for docking.

The saved SDF files of compounds were directly added into PyRx for docking since PyRx has access to Open Babel [73,74]. The charge of each compound was minimized, and those minimized charged compounds were converted into a PDBQT file to continue the docking process [75].

#### 4.12.2. Identification of Binding Site and Grid Box for Protein

The binding sites/active sites for each target were identified by using Discovery Studio’s predictions and the grid boxes optimized around the binding areas suggested by the software [76,77,78]. Active sites help to adjust the grid box according to protein. Discovery Studio is a useful software and helped to find out the active sites quickly [79]. While the binding affinity is an important parameter, ligand interactions also depend on chemical properties, steric interactions, and protein flexibility, which influence binding to specific residues [80,81,82]. For IL10 (PDB ID: 2H24), LEU26, PHE30, MET68, ILE69, TYR72, LEU73, VAL76, MET77, DER93, LEU94, GLY95, and LEU98 were the amino acids for the active site. LEU85, ARG89, CYS132, PRO133, ALA136, SER159, SER161, ASN215, TYR218, GLN220, and LEU222 were the identified amino acids of the active site for FASLG (PDB ID: 4MSV). Furthermore, PRO244, PRO245, LYS246, GLU258, THR260, ASP265, THR299, ARG301, GLN295, HIS44, ASP64, SER66, and ARG155 were the identified amino acids of the active site for FCGR3A (PDB ID: 3AY4). Moreover, ARG637, TYR658, VAL659, ARG736, LYS736, HIS797, SER828, SER829, ALA830, GLY831, VAL 832, GLY833, and ARG834 were identified for PTPRC (PDB ID: 1YGR).

The grid box was set into the center: X = 15.7665, Y = 26.4005, Z = 13.5283 and dimensions: X = 32.4150, Y = 34.1901, Z = 27.6981 for IL10 (PDB ID: 2H24). The box was fixed into the center: X = −18.0398, Y = −7.5283, Z = −52.8173 and dimensions: X = 37.2960, Y = 43.1758, Z = 30.4286 for FASLG (PDB ID: 4MSV). The box was selected into the center: X = 18.0506, Y = −8.2066, Z = 55.3804 and dimensions: X = 39.4536, Y = 42.7338, Z = 36.2982 for PTPRC (PDB ID: 1YGR). Finally, for FCGR3A (PDB ID: 3AY4), the grid box was fixed into the center: X = 9.2275, Y = 22.3744, Z = 110.7291 and dimensions: X = 26.8820, Y = 25.0730, Z = 38.1790.

### 4.13. Alternative Docking

To further validate the docking results, molecular docking was performed using SwissDock, a web-based docking service employing the EADock DSS algorithm [83]. This alternative approach was used to cross-check the binding affinities of selected compounds with the target proteins, ensuring consistency with previous docking findings.

For this online tool, the targets were used directly in the PDB format, as it has an option for preparing proteins [84]. The smiles of the compound were inserted and prepared by the tool [84]. The grid box size and position for each protein were set according to the active site of each protein, which was identified by Discovery Studio in the previous method.

## 5. Conclusions

This study employed a systems biology-driven approach integrating network pharmacology, molecular docking, and drug repurposing to identify host-targeted therapeutics against the Oropouche virus (OROV). By prioritizing key host proteins—IL10, FASLG, PTPRC, and FCGR3A—we uncovered their critical roles in immune modulation and viral pathogenesis. Deptropine and Acetohexamide emerged as promising candidates, demonstrating strong binding affinities across multiple targets, suggesting their potential as broad-spectrum antiviral agents.

Our findings highlight the viability of host-directed antiviral strategies to circumvent viral resistance, a major limitation of direct-acting antivirals. The computational validation of these compounds, supported by multi-docking analyses, provides a robust foundation for further experimental studies. However, in vitro and in vivo assays are essential to confirm their efficacy and safety.

This research bridges a critical gap in OROV therapeutics, offering a cost-effective, rapid-response framework for neglected tropical diseases. Future work should incorporate multi-omics data, molecular dynamics simulations, and clinical trials to advance these findings toward real-world applications. By leveraging computational drug discovery, this study contributes to pandemic preparedness and underscores the importance of host-targeted interventions in combating emerging arboviral threats.

## Figures and Tables

**Table 1:** Different pharmacokinetic criteria.

Name	Weight	H-Bond Acceptor	H-Bond Donor	TPSA	MlogP
3-Azido-3-deoxythymidine	267.24 g/mol	7	2	134.07	−1.25
Retinoic Acid	300.44 g/mol	2	1	37.30	-
Methotrexate	454.44 g/mol	9	5	210.54	−1.13
Deptropine	333.47 g/mol	2	0	12.47	−4.12
Acetohexamide	324.40 g/mol	4	2	100.72	1.12

**Table 2:** Different MCODE cluster analysis.

Color	MCODE	GO	Description	log_10_(*p*)
Red	MCODE_1	R-HSA-2029480	Fc-gamma receptor (FCGR)-dependent phagocytosis	−17.9
Red	MCODE_1	R-HSA-2029481	FCGR activation	−17.3
Red	MCODE_1	GO:0038094	Fc-gamma receptor signaling pathway	−16.3
Blue	MCODE_2	hsa05235	PD-L1 expression and PD-1 checkpoint pathway in cancer	−11.9
Blue	MCODE_2	WP4255	Non-small cell lung cancer	−9.4
Blue	MCODE_2	hsa05223	Non-small cell lung cancer	−9.3
Green	MCODE_3	R-HSA-3700989	Transcriptional regulation by TP53	−6.9
Green	MCODE_3	R-HSA-9700026	Signaling by ALK in cancer	−6.7
Green	MCODE_3	R-HSA-9725370	Signaling by ALK fusions and activated point mutants	−6.7

**Table 3:** Comparison of SWISSDOCK and original docking results for molecular binding analysis.

Compound Name and CID	Protein Name and Binding Affinity							
	PyRx	SwissDock	PyRx	SwissDock	PyRx	SwissDock	PyRx	SwissDock
	IL10		FASLG		PTPRC		FCGR3A	
Acetohexamide, CID: 1989	−7.3 kcal/mol	−7.3 kcal/mol	−6.5 kcal/mol	−6.8 kcal/mol	−7.3 kcal/mol	−7.1 kcal/mol	−6.7 kcal/mol	−7.0 kcal/mol
Deptropine, CID: 203911	−8.3 kcal/mol	−7.0 kcal/mol	−7.4 kcal/mol	−6.9 kcal/mol	−7.0 kcal/mol	−6.4 kcal/mol	−7.1 kcal/mol	−6.8 kcal/mol
Methotrexate, CID: 126941	−6.8 kcal/mol	−7.8 kcal/mol	−7.4 kcal/mol	−7.4 kcal/mol	−7.7 kcal/mol	−7.4 kcal/mol	−6.7 kcal/mol	−8.1 kcal/mol
Retinoic Acid, CID: 449171	−8.1 kcal/mol	−7.4 kcal/mol	−6.7 kcal/mol	−7.1 kcal/mol	−6.3 kcal/mol	−7.1 kcal/mol	−5.8 kcal/mol	−6.9 kcal/mol
3-Azido-3-deoxythymidine, CID: 5726	−5.3 kcal/mol	−6.8 kcal/mol	−5.7 kcal/mol	−6.8 kcal/mol	−5.6 kcal/mol	−6.8 kcal/mol	−5.1 kcal/mol	−6.7 kcal/mol

**Table 4:** Binding affinity of the target proteins with their known inhibitors.

Compound Name and CID	Protein Name	Interaction Between Protein and Compound	Binding Affinity
2-(4-Acetylanilino)-3-chloronaphthoquinone; CID: 781109	PTPRC	PTPRC + 2-(4-Acetylanilino)-3-chloronaphthoquinone	−7.3 kcal/mol
JTE-607; CID: 9938544	IL10	IL10 + JTE-607	−6.7 kcal/mol
BI-1206; CID: 170872117	FCGR3A	FCGR3A + BI-1206	−6.5 kcal/mol
ONL1204; CID: 122677428	FASLG	FASLG + ONL1204	−7.7 kcal/mol

## Data Availability

Data is contained within the article or supplementary material.

## Supplementary Materials

The following supporting information can be downloaded at: https://www.mdpi.com/article/10.3390/ph18050613/s1. Table S1: Target genes common in both virus and drugs. Table S2: List of biological processes. Table S3: List of cellular components. Table S4: List of molecular functions. Table S5: List of keg pathway. Table S6: Targets with different topological properties. Figure S1: Molecular interaction of IL10 with the selected compound (IL10 + Acetohexamide). Figure S2: Molecular interaction of IL10 with the selected compound (IL10 + Methotrexate). Figure S3: Molecular interaction of IL10 with the selected compound (IL10 + Retinoic acid). Figure S4: Molecular interaction of IL10 with the selected compound (IL10 + 3-Azido-3-deoxythymidine). Figure S5: Molecular interaction of FASLG with the selected compounds (FASLG + Acetohexamide). Figure S6: Molecular interaction of FASLG with the selected compounds (FASLG + Methotrexate). Figure S7: Molecular interaction of FASLG with the selected compounds (FASLG + Retinoic acid). Figure S8: Molecular interaction of FASLG with the selected compounds (FASLG + 3-Azido-3-deoxythymidine). Figure S9: Molecular interaction of PTPRC with the selected compound (PTPRC + Deptropine). Figure S10: Molecular interaction of PTPRC with the selected compound (PTPRC + Methotrexate). Figure S11: Molecular interaction of PTPRC with the selected compound (PTPRC + Retinoic acid). Figure S12: Molecular interaction of PTPRC with the selected compound (PTPRC + 3-Azido-3-deoxythymidine). Figure S13: Molecular interaction of FCGR3A with the selected compound (FCGR3A + Acetohexamide). Figure S14: Molecular interaction of FCGR3A with the selected compound (FCGR3A + Methotrexate). Figure S15: Molecular interaction of FCGR3A with the selected compound (FCGR3A + Retinoic acid). Figure S16: Molecular interaction of FCGR3A with the selected compound (FCGR3A + 3-Azido-3-deoxythymidine).
