# Supplementary material for: Systems Biology-Driven Discovery of Host-Targeted Therapeutics for Oropouche Virus: Integrating Network Pharmacology, Molecular Docking, and Drug Repurposing
Source: Pharmaceuticals (Basel). 2025 Apr 23;18(5):613. doi: 10.3390/ph18050613 (PMC12114254; doi:10.3390/ph18050613)
Supplement: Supplementary file 1 [file pharmaceuticals-18-00613-s001.zip › pharmaceuticals-3554853-supplementary.pdf]

Supplementary materials

Table S1: Target genes common in both virus and drugs

Common targets													
FASLG	IFIT1	JUN	DNMT3A	GFI1	EBNA1BP2	MTHFR	ACADM	CD244	EIF2AK2	IL6R	PIK3C2B	SLAMF7	XPR1
IL10	IRF7	MEIS1	GBA1	IFI44	EPHA2	NCF2	ACKR1	CD247	EIF2B3	IRF3	PNPT1	SMC6	ZDHHC18
SDHB	ISG15	MYCN	MAPKAPK2	KDM1A	FCGR3A	PIK3CD	ACTR2	CD46	EML4	KHDRBS1	PRDM2	SORT1	RORC
ATF3	MOV10	APOA2	NR5A2	PLA2G2A	GTF2H2	POLR3C	ADAR	CD48	FAM72A	KIAA0319L	RAD54L	SPOCK3	AMPD1
BST2	MTOR	DUSP10	TNNT2	RPE65	HDAC1	PTPN22	AIM2	CMPK2	FBXO6	LAMTOR5	RNASEH1	TENT5C	PCBP1
CD1C	PTGS2	F11R	ACTN2	SYTL1	IGSF8	PTPRC	AKT3	CPT2	FCGR2A	MXRA8	RNASEL	TNFRSF4	CFHR1
FCGR1A	REL	FGR	AGMAT	TNFRSF14	IVNS1ABP	SDC1	ARPC5	CR1	GALE	NECTIN4	RNF115	TNFSF4	EVI5
HAX1	RSAD2	ALK	CHI3L1	TRAF5	LCK	SKI	BATF3	CR2	HIVEP3	NLRC4	RPL22	TOE1	HADHB
IFI44L	SLC16A1	ATF6	CTPS1	WASF2	LGALS8	TGOLN2	BROX	DDX1	HNRNPU	NLRP3	SDC3	VRK2	LMNA
IFI6	SLC2A1	CD55	FCGR2B	ADI1	LYST	TNFRSF9	CCT4	DDX20	IFNAR1	PI4KB	SLAMF6	XPO1	MYCL
NRAS	NRAS	NRAS	NRAS	NRAS	NRAS								

Table S2: List of biological processes

Category Term Count % PValue Genes List Total Pop Hits Pop Total Fold Enrichment Bonferroni Benjamini FDR
GOTERM_BP_DIRECT GO:0009615~response to virus 15 10.273972602739725 7.266340167325391E-14 IVNS1ABP, BATF3, RSAD2, IFI44, EIF2AK2, ISG15, ADAR, IFIT1, BST2, FGR, TNFSF4, IRF7, XPR1, LAMTOR5, IFNAR1 144 110 19416 18.386363636363637 1.0303158326507855E-10 1.031093669743473E-10 1.0063881131745666E-10
GOTERM_BP_DIRECT GO:0051607~defense response to virus 19 13.013698630136986 1.8104357890169632E-13 RNASEL, RSAD2, DDX1, IFI6, EIF2AK2, ISG15, ADAR, IFIT1, LYST, IFI44L, BST2, MOV10, AIM2, PTPRC, IRF3, POLR3C, ZC3H12A, IRF7, NLRP3 144 239 19416 10.718967921896793 2.5694879557391914E-10 1.2845041923075353E-10 1.253726783894247E-10

GOTERM_BP_DIRECT	GO:0045087~innate immune response	25	17.123287671232877	3.564824844713974E-12	NCF2, DDX1, IFI6, PIK3CD, ADAR, NLRC4, ZDHHC18, NLRP3, SLAMF6, TNFRSF14, FCGR1A, CD55, SLAMF1, RSAD2, ISG15, FGR, BST2, AIM2, IRF3, LCK, POLR3C, REL, IRF7, CD46, CD244	144	564	19416	5.9766548463356965	5.0584726407620906E-9	1.6861621515497096E-9	1.645760803309618E-9
GOTERM_BP_DIRECT	GO:0046718~symbiont entry into host cell	11	7.534246575342466	6.539396083373773E-9	CR2, CR1, TNFRSF14, F11R, CD46, NECTIN4, TNFRSF4, CD55, XPR1, EPHA2, SLAMF1	144	107	19416	13.861370716510905	9.279360093428224E-6	2.3198507605768457E-6	2.264265893868169E-6
GOTERM_BP_DIRECT	GO:0038094~Fc-gamma receptor signaling pathway	6	4.10958904109589	3.834083074416919E-8	FCGR3A, FCGR2A, LCK, CD247, FCGR1A, FCGR2B	144	14	19416	57.785714285714285	5.440415989244052E-5	1.0881127765195216E-5	1.0620410116134866E-5
GOTERM_BP_DIRECT	GO:0006955~immune response	19	13.013698630136986	7.033709986143472E-8	IL10, CR2, IFI6, IFI44, PIK3CD, FASLG, CD1C, IFI44L, FCGR3A, AIM2, TNFSF4, SLAMF7, CD48, SLAMF6, TNFRSF14, FCGR1A, TNFRSF4, CD244, SLAMF1	144	529	19416	4.842785129174543	9.980336745474272E-5	1.6634724117229313E-5	1.6236147218014514E-5
GOTERM_BP_DIRECT	GO:0006954~inflammatory response	15	10.273972602739725	3.1133473177677303E-6	PLA2G2A, PIK3CD, NLRC4, F11R, MTOR, TNFSF4, MAPKAPK2, ZC3H12A, REL, NLRP3, CHI3L1, ACKR1, FCGR2B, TNFRSF4, EPHA2	144	424	19416	4.770047169811321	0.004408102391114999	6.311199777017728E-4	6.159980050154724E-4
GOTERM_BP_DIRECT	GO:0045071~negative regulation of viral genome replication	6	4.10958904109589	1.7411964551535285E-5	BST2, RNASEL, RSAD2, EIF2AK2, ISG15, IFIT1	144	44	19416	18.386363636363633	0.02440505375535873	0.003088447212328571	0.003014446362984546
GOTERM_BP_DIRECT	GO:0050852~T cell receptor signaling pathway	8	5.47945205479452	3.182296206545587E-5	KHDRBS1, EIF2B3, PTPRC, LCK, ZC3H12A, PIK3CD, PTPN22, CD247	144	121	19416	8.914600550964188	0.04415307753608955	0.005017420352320209	0.004897200273406265
GOTERM_BP_DIRECT	GO:0001788~antibody-dependent cellular cytotoxicity	4	2.73972602739726	4.5200265841928956E-5	FCGR3A, FCGR2A, FCGR1A, FCGR2B	144	10	19416	53.93333333333333	0.062126899861912555	0.005830834293608835	0.005691124381006509
GOTERM_BP_DIRECT	GO:0002819~regulation of adaptive immune response	4	2.73972602739726	4.5200265841928956E-5	DUSP10, TNFSF4, IRF7, FCGR2B	144	10	19416	53.93333333333333	0.062126899861912555	0.005830834293608835	0.005691124381006509
GOTERM_BP_DIRECT	GO:0030101~natural killer cell activation	5	3.4246575342465753	6.66177305285075E-5	FCGR3A, PIK3CD, SLAMF7, CD48, CD244	144	30	19416	22.472222222222225	0.09020293375386679	0.00749864087108421	0.007318969419627646
GOTERM_BP_DIRECT	GO:0098586~cellular response to virus	7	4.794520547945205	6.869790790986239E-5	IRF3, ZC3H12A, IFI6, NLRP3, ADAR, LGALS8, IFNAR1	144	94	19416	10.040780141843971	0.09288466854911526	0.00749864087108421	0.007318969419627646
GOTERM_BP_DIRECT	GO:0032728~positive regulation of interferon-beta production	5	3.4246575342465753	2.5407296518104125E-4	IRF3, POLR3C, IRF7, ISG15, PTPN22	144	42	19416	16.051587301587304	0.3027249635906947	0.025752109827992684	0.02513507548398158
GOTERM_BP_DIRECT	GO:0051092~positive regulation of NF-kappaB transcription factor activity	7	4.794520547945205	2.8820534676457163E-4	ALK, AIM2, TRAF5, NLRP3, EIF2AK2, NLRC4, LAMTOR5	144	122	19416	7.7363387978142075	0.3357006023809479	0.026069742659643597	0.02544509766286567

GOTERM_BP_DIRECT GO:0032729~positive regulation of type II interferon production 6 4.10958904109589 2.9395058671902576E-4 TNFSF4, ISG15, SLAMF6, PTPN22, CD244, SLAMF1 144 79 19416 10.240506329113924 0.3410958484146056 0.026069742659643597 0.02544509766286567
GOTERM_BP_DIRECT GO:0032689~negative regulation of type II interferon production 5 3.4246575342465753 3.620703115875208E-4 IL10, CR1, TNFSF4, ZC3H12A, SLAMF1 144 46 19416 14.655797101449275 0.4018243304842658 0.030222221890746588 0.029498081267571547
GOTERM_BP_DIRECT GO:0006338~chromatin remodeling 18 12.32876712328767 4.240467482798223E-4 ALK, KDM1A, HDAC1, DDX1, DDX20, EIF2AK2, PTPN22, PRDM2, VRK2, MTOR, FGR, DUSP10, PTPRC, LCK, MAPKAPK2, AKT3, RAD54L, EPHA2 144 917 19416 2.6466739367502727 0.45220270006318475 0.03342901865605932 0.0326280414648641
GOTERM_BP_DIRECT GO:0050766~positive regulation of phagocytosis 5 3.4246575342465753 5.805077684965591E-4 FCGR2A, PTPRC, APOA2, FCGR1A, FCGR2B 144 52 19416 12.964743589743591 0.5613176525573753 0.04335476439455881 0.04231596101935444
GOTERM_BP_DIRECT GO:0071222~cellular response to lipopolysaccharide 8 5.47945205479452 6.704711742638166E-4 IL10, HADHB, GFI1, TNFSF4, ZC3H12A, NLRP3, CMPK2, PTGS2 144 198 19416 5.447811447811447 0.6139227042781875 0.04756992981401779 0.0464301288177693
GOTERM_BP_DIRECT GO:0045944~positive regulation of transcription by RNA polymerase II 21 14.383561643835616 8.173738098385162E-4 IL10, ACTR2, JUN, RNASEL, KDM1A, HDAC1, HNRNPU, PRDM2, SKI, MEIS1, MYCN, HAX1, NR5A2, IRF3, PCBP1, ZC3H12A, REL, IRF7, NLRP3, ATF6, ATF3 144 1246 19416 2.2724719101123596 0.6866165353216729 0.05523111600765973 0.0539077488869688
GOTERM_BP_DIRECT GO:0002639~positive regulation of immunoglobulin production 4 2.73972602739726 0.001242253435388665 IL10, PTPRC, TNFSF4, TNFRSF4 144 29 19416 18.597701149425287 0.8286168230202009 0.0801253465825689 0.07820550036424095
GOTERM_BP_DIRECT GO:0045959~negative regulation of complement activation, classical pathway 3 2.054794520547945 0.0014650617166585775 CR2, CR1, CD46 144 8 19416 50.5625 0.8751256116242919 0.08380463806988322 0.08179663405693323
GOTERM_BP_DIRECT GO:0006468~protein phosphorylation 9 6.164383561643835 0.0014671738000792164 FGR, RNASEL, LCK, AKT3, MAPKAPK2, EIF2AK2, PIK3CD, VRK2, MTOR 144 292 19416 4.155821917808219 0.875499853549148 0.08380463806988322 0.08179663405693323
GOTERM_BP_DIRECT GO:0032760~positive regulation of tumor necrosis factor production 6 4.10958904109589 0.0015592484186352663 FCGR3A, FCGR2A, PTPRC, MAPKAPK2, FCGR1A, FCGR2B 144 114 19416 7.096491228070175 0.8907700818129299 0.08380463806988322 0.08179663405693323
GOTERM_BP_DIRECT GO:0001819~positive regulation of cytokine production 5 3.4246575342465753 0.001594591422048518 IL10, FGR, TNFSF4, EIF2AK2, PIK3CD 144 68 19416 9.91421568627451 0.8961212794760738 0.08380463806988322 0.08179663405693323
GOTERM_BP_DIRECT GO:0042110~T cell activation 5 3.4246575342465753 0.001594591422048518 PTPRC, LCK, PIK3CD, SLAMF7, SLAMF6 144 68 19416 9.91421568627451 0.8961212794760738 0.08380463806988322 0.08179663405693323
GOTERM_BP_DIRECT GO:0032715~negative regulation of interleukin-6 production 5 3.4246575342465753 0.0016831527760632385 IL10, ZC3H12A, GBA1, PTPN22, SLAMF1 144 69 19416 9.770531400966183 0.9084075160252143 0.08529977818691913 0.08325594981598519

GOTERM_BP_DIRECT	GO:0000122~negative regulation of transcription by RNA polymerase II	17	11.643835616438356	0.00187197636386373	KHDRBS1, JUN, BATF3, KDM1A, HDAC1, GFI1, DNMT3A, RORC, DDX20, HNRNPU, FASLG, PRDM2, SKI, PTPRC, IRF3, IRF7, ATF3	144	958	19416	2.3926583159359778	0.9299694613196904	0.09159774001112526	0.0894030091017678
GOTERM_BP_DIRECT	GO:0006974~DNA damage response	9	6.164383561643835	0.0022194313552838124	AIM2, IRF3, GFI1, ZC3H12A, MAPKAPK2, IRF7, VRK2, SMC6, MTOR	144	312	19416	3.889423076923077	0.9572708183691188	0.10497910310492432	0.102463747568936
GOTERM_BP_DIRECT	GO:0035455~response to interferon-alpha	3	2.054794520547945	0.002836365794812329	BST2, EIF2AK2, ADAR	144	11	19416	36.772727272727266	0.982234942021199	0.12983235686576436	0.12672150405855084
GOTERM_BP_DIRECT	GO:0009410~response to xenobiotic stimulus	8	5.47945205479452	0.002983947893486602	IL10, LCK, DNMT3A, APOA2, RAD54L, MTHFR, CTPS1, PTGS2	144	257	19416	4.197146562905318	0.9856003590270122	0.1323194394017965	0.129148994764967
GOTERM_BP_DIRECT	GO:0061014~positive regulation of mRNA catabolic process	3	2.054794520547945	0.0033872438889481094	MOV10, PNPT1, ZC3H12A	144	12	19416	33.708333333333336	0.9918900039660582	0.13991350901011335	0.13656110639817265
GOTERM_BP_DIRECT	GO:0030217~T cell differentiation	4	2.73972602739726	0.003398039727584276	PTPRC, LCK, PIK3CD, PTPN22	144	41	19416	13.154471544715447	0.9920137130928303	0.13991350901011335	0.13656110639817265
GOTERM_BP_DIRECT	GO:0050731~positive regulation of peptidyl-tyrosine phosphorylation	5	3.4246575342465753	0.0034510026887624857	HAX1, PTPRC, TNFRSF14, IL6R, MTOR	144	84	19416	8.025793650793652	0.9925938314562537	0.13991350901011335	0.13656110639817265
GOTERM_BP_DIRECT	GO:0050830~defense response to Gram-positive bacterium	6	4.10958904109589	0.0038048753934785827	FGR, PLA2G2A, NLRP3, TNFRSF14, IL6R, EPHA2	144	140	19416	5.7785714285714285	0.995525746685926	0.14997550509294746	0.14638201166577325
GOTERM_BP_DIRECT	GO:0032496~response to lipopolysaccharide	6	4.10958904109589	0.004415976270676216	DUSP10, MAPKAPK2, FASLG, PTPN22, PTGS2, IFNAR1	144	145	19416	5.579310344827586	0.998126874862592	0.16935865751593382	0.16530073337531243
GOTERM_BP_DIRECT	GO:0006958~complement activation, classical pathway	4	2.73972602739726	0.004711104273308894	CR2, CR1, CD46, CD55	144	46	19416	11.72463768115942	0.9987701383512596	0.17592255167961374	0.1717073531192847
GOTERM_BP_DIRECT	GO:0042100~B cell proliferation	4	2.73972602739726	0.005310729251371325	IL10, CR2, PTPRC, CTPS1	144	48	19416	11.23611111111111	0.9994770405166513	0.19322884122297207	0.1885989746961355
GOTERM_BP_DIRECT	GO:0060337~type I interferon-mediated signaling pathway	4	2.73972602739726	0.00595422639763306	CR2, IRF3, IRF7, IFNAR1	144	50	19416	10.786666666666665	0.9997912349965218	0.19935256651946212	0.1945759722547252
GOTERM_BP_DIRECT	GO:0051384~response to glucocorticoid	4	2.73972602739726	0.00595422639763306	IL10, APOA2, SDC1, PTGS2	144	50	19416	10.786666666666665	0.9997912349965218	0.19935256651946212	0.1945759722547252
GOTERM_BP_DIRECT	GO:0002456~T cell mediated immunity	3	2.054794520547945	0.006040986864226125	CR2, CR1, CD46	144	16	19416	25.28125	0.9998155546354163	0.19935256651946212	0.1945759722547252
GOTERM_BP_DIRECT	GO:0001779~natural killer cell differentiation	3	2.054794520547945	0.006040986864226125	PTPRC, PIK3CD, SLAMF1	144	16	19416	25.28125	0.9998155546354163	0.19935256651946212	0.1945759722547252

GOTERM_BP_DIRECT	GO:0043123~positive regulation of canonical NF-kappaB signal transduction	7	4.794520547945205	0.0066336841764492	BST2, IRF3, DDX1, TRAF5, REL, FASLG, LAMTOR5	144	226	19416	4.176253687315634	0.9999208810946574	0.21393631469048668	0.20881028600868504
GOTERM_BP_DIRECT	GO:0002221~pattern recognition receptor signaling pathway	3	2.054794520547945	0.006813568632640063	AIM2, NLRP3, NLRC4	144	17	19416	23.794117647058822	0.9999388109688431	0.21485453088258333	0.20970650124903303
GOTERM_BP_DIRECT	GO:0042742~defense response to bacterium	6	4.10958904109589	0.007565735265184265	IL10, PLA2G2A, ISG15, NLRC4, FCGR1A, LYST	144	165	19416	4.903030303030302	0.9999791174437985	0.2303148814210866	0.22479641350824875
GOTERM_BP_DIRECT	GO:0045591~positive regulation of regulatory T cell differentiation	3	2.054794520547945	0.007628470350099416	CR1, DUSP10, CD46	144	18	19416	22.47222222222222	0.9999809091032054	0.2303148814210866	0.22479641350824875
GOTERM_BP_DIRECT	GO:0033209~tumor necrosis factor-mediated signaling pathway	4	2.73972602739726	0.008155760291573892	AIM2, TRAF5, TNFRSF14, TNFRSF4	144	56	19416	9.630952380952381	0.9999910196835473	0.24110466361965316	0.235327666746455
GOTERM_BP_DIRECT	GO:0042981~regulation of apoptotic process	7	4.794520547945205	0.008791154882930889	ALK, KHDRBS1, HAX1, IRF3, ACTN2, TRAF5, NLRC4	144	240	19416	3.932638888888889	0.9999963827828209	0.2545846689567129	0.2484846839359037
GOTERM_BP_DIRECT	GO:0050729~positive regulation of inflammatory response	5	3.4246575342465753	0.00920095663439293	AIM2, TNFSF4, PLA2G2A, NLRP3, NLRC4	144	111	19416	6.073573573573574	0.9999979884259425	0.2611231492840714	0.25486649877268414
GOTERM_BP_DIRECT	GO:0032757~positive regulation of interleukin-8 production	4	2.73972602739726	0.012250740794793357	APOA2, CHI3L1, CD244, LAMTOR5	144	65	19416	8.297435897435896	0.9999999746670809	0.3408588468198387	0.3326916862899765
GOTERM_BP_DIRECT	GO:0010628~positive regulation of gene expression	10	6.8493150684931505	0.012583337282388057	MYCN, HDAC1, LMNA, ZC3H12A, PIK3CD, PTPN22, CD46, ATF3, MTOR, LAMTOR5	144	505	19416	2.6699669966996704	0.9999999842912745	0.34337991545593566	0.3351523487712973
GOTERM_BP_DIRECT	GO:0048015~phosphatidylinositol-mediated signaling	3	2.054794520547945	0.014463513930216589	PIK3CD, PI4KB, PIK3C2B	144	25	19416	16.18	0.9999999989490757	0.3800690049440248	0.37096234802499956
GOTERM_BP_DIRECT	GO:0045088~regulation of innate immune response	3	2.054794520547945	0.014463513930216589	FGR, PTPN22, FCGR2B	144	25	19416	16.18	0.9999999989490757	0.3800690049440248	0.37096234802499956
GOTERM_BP_DIRECT	GO:0007166~cell surface receptor signaling pathway	8	5.47945205479452	0.015362227989288126	FCGR3A, FCGR2A, PTPRC, TRAF5, TNFRSF14, CD247, FCGR1A, FCGR2B	144	351	19416	3.0731244064577394	0.9999999997120335	0.3815139969641382	0.37237271726239
GOTERM_BP_DIRECT	GO:0006909~phagocytosis	4	2.73972602739726	0.015527815842396937	NCF2, FCGR2B, LYST, SLAMF1	144	71	19416	7.596244131455399	0.9999999997731727	0.3815139969641382	0.37237271726239
GOTERM_BP_DIRECT	GO:0055013~cardiac muscle cell development	3	2.054794520547945	0.01559394772651164	ACTN2, HNRNPU, MTOR	144	26	19416	15.557692307692308	0.9999999997937963	0.3815139969641382	0.37237271726239

GOTERM_BP_DIRECT	GO:0032727~positive regulation of interferon-alpha production	3	2.054794520547945	0.01559394772651164	IRF3, IRF7, PTPN22	144	26	19416	15.557692307692308	0.9999999997937963	0.3815139969641382	0.37237271726239
GOTERM_BP_DIRECT	GO:0006898~receptor-mediated endocytosis	4	2.73972602739726	0.0161173182392245	SDC1, PI4KB, FCGR1A, FCGR2B	144	72	19416	7.4907407407407405	0.999999999903046	0.38763516239761975	0.37834721629365986
GOTERM_BP_DIRECT	GO:0001938~positive regulation of endothelial cell proliferation	4	2.73972602739726	0.01671927520613003	IL10, NRAS, AKT3, PIK3CD	144	73	19416	7.3881278538812785	0.9999999999593173	0.39541085862497516	0.3859366026748348
GOTERM_BP_DIRECT	GO:0007165~signal transduction	18	12.32876712328767	0.017986979911270663	ALK, APOA2, PIK3CD, FASLG, VRK2, FGR, DUSP10, PTPRC, TNFSF4, AKT3, TRAF5, NLRP3, CD48, PI4KB, FCGR1A, NECTIN4, ATF6, CD244	144	1326	19416	1.830316742081448	0.9999999999934781	0.41841843432939463	0.4083929045427847
GOTERM_BP_DIRECT	GO:0045070~positive regulation of viral genome replication	3	2.054794520547945	0.019202891267834466	NR5A2, ADAR, IFIT1	144	29	19416	13.948275862068964	0.9999999999988758	0.4394984307912437	0.42896781299920544
GOTERM_BP_DIRECT	GO:0046777~protein autophosphorylation	5	3.4246575342465753	0.019561935662993222	FGR, ALK, EIF2AK2, VRK2, MTOR	144	139	19416	4.850119904076739	0.9999999999993313	0.44060931279027593	0.4300520776705653
GOTERM_BP_DIRECT	GO:0035458~cellular response to interferon-beta	3	2.054794520547945	0.02047646352889117	AIM2, PNPT1, IFNAR1	144	30	19416	13.483333333333333	0.9999999999998221	0.4470169499614857	0.43630618442329644
GOTERM_BP_DIRECT	GO:0010592~positive regulation of lamellipodium assembly	3	2.054794520547945	0.02047646352889117	ACTR2, WASF2, MTOR	144	30	19416	13.483333333333333	0.9999999999998221	0.4470169499614857	0.43630618442329644
GOTERM_BP_DIRECT	GO:0032720~negative regulation of tumor necrosis factor production	4	2.73972602739726	0.021283252628517643	IL10, ZC3H12A, PTPN22, SLAMF1	144	80	19416	6.741666666666666	0.9999999999999447	0.45107681634040236	0.4402687742293568
GOTERM_BP_DIRECT	GO:0045916~negative regulation of complement activation	2	1.36986301369863	0.021933967813592505	CR1, CD55	144	3	19416	89.88888888888889	0.9999999999999785	0.45107681634040236	0.4402687742293568
GOTERM_BP_DIRECT	GO:0045607~regulation of inner ear auditory receptor cell differentiation	2	1.36986301369863	0.021933967813592505	MYCL, MYCN	144	3	19416	89.88888888888889	0.9999999999999785	0.45107681634040236	0.4402687742293568
GOTERM_BP_DIRECT	GO:0071663~positive regulation of granzyme B production	2	1.36986301369863	0.021933967813592505	PTPN22, CD244	144	3	19416	89.88888888888889	0.9999999999999785	0.45107681634040236	0.4402687742293568
GOTERM_BP_DIRECT	GO:0006952~defense response	4	2.73972602739726	0.02416735657579873	NLRP3, CD48, ACKR1, FCGR2B	144	84	19416	6.420634920634921	0.9999999999999991	0.48967332207061603	0.4779404870104321
GOTERM_BP_DIRECT	GO:0070269~pyroptotic inflammatory response	3	2.054794520547945	0.024500920272736953	AIM2, NLRP3, NLRC4	144	33	19416	12.257575757575758	0.9999999999999994	0.48967332207061603	0.4779404870104321
GOTERM_BP_DIRECT	GO:0030183~B cell differentiation	4	2.73972602739726	0.026462278297560052	IL10, CR2, PTPRC, PIK3CD	144	87	19416	6.199233716475096	1.0	0.521527401447746	0.5090313255850093

GOTERM_BP_DIRECT	GO:0002230~positive regulation of defense response to virus by host	3	2.054794520547945	0.027348081063300726	AIM2, ZC3H12A, PTPN22	144	35	19416	11.557142857142857	1.0	0.531601740120873	0.5188642777078288
GOTERM_BP_DIRECT	GO:0016310~phosphorylation	3	2.054794520547945	0.02881927327496404	ALK, PIK3CD, MTOR	144	36	19416	11.23611111111111	1.0	0.5378274290308239	0.5249407957770903
GOTERM_BP_DIRECT	GO:0046500~S-adenosylmethionine metabolic process	2	1.36986301369863	0.029138595774783314	DNMT3A, MTHFR	144	4	19416	67.41666666666666	1.0	0.5378274290308239	0.5249407957770903
GOTERM_BP_DIRECT	GO:0039530~MDA-5 signaling pathway	2	1.36986301369863	0.029138595774783314	IRF3, IRF7	144	4	19416	67.41666666666666	1.0	0.5378274290308239	0.5249407957770903
GOTERM_BP_DIRECT	GO:0032717~negative regulation of interleukin-8 production	3	2.054794520547945	0.030321490008785	IL10, PTPN22, IL6R	144	37	19416	10.932432432432432	1.0	0.5378274290308239	0.5249407957770903
GOTERM_BP_DIRECT	GO:0042267~natural killer cell mediated cytotoxicity	3	2.054794520547945	0.030321490008785	FCGR3A, SLAMF7, LYST	144	37	19416	10.932432432432432	1.0	0.5378274290308239	0.5249407957770903
GOTERM_BP_DIRECT	GO:0008340~determination of adult lifespan	3	2.054794520547945	0.030321490008785	RAD54L, GBA1, PRDM2	144	37	19416	10.932432432432432	1.0	0.5378274290308239	0.5249407957770903
GOTERM_BP_DIRECT	GO:0048709~oligodendrocyte differentiation	3	2.054794520547945	0.030321490008785	DUSP10, HDAC1, MTOR	144	37	19416	10.932432432432432	1.0	0.5378274290308239	0.5249407957770903
GOTERM_BP_DIRECT	GO:0032481~positive regulation of type I interferon production	3	2.054794520547945	0.0318542013704845	IRF3, IRF7, PTPN22	144	38	19416	10.644736842105264	1.0	0.5580384166014507	0.5446675172607536
GOTERM_BP_DIRECT	GO:0050776~regulation of immune response	3	2.054794520547945	0.03341688348913552	FCGR3A, IRF7, FCGR2B	144	39	19416	10.37179487179487	1.0	0.5713079237479917	0.5576190799090686
GOTERM_BP_DIRECT	GO:0048255~mRNA stabilization	3	2.054794520547945	0.03341688348913552	TRAF5, HNRNPU, TENT5C	144	39	19416	10.37179487179487	1.0	0.5713079237479917	0.5576190799090686
GOTERM_BP_DIRECT	GO:0140588~chromatin looping	5	3.4246575342465753	0.03514661153767429	MOV10, DDX1, DDX20, RAD54L, SMC6	144	167	19416	4.036926147704591	1.0	0.5906602704872904	0.5765077340556005
GOTERM_BP_DIRECT	GO:0008284~positive regulation of cell population proliferation	9	6.164383561643835	0.035604471666848644	MEIS1, CR1, HDAC1, TNFSF4, TRAF5, FASLG, IL6R, ATF3, SLAMF1	144	511	19416	2.3747553816046967	1.0	0.5906602704872904	0.5765077340556005
GOTERM_BP_DIRECT	GO:1905037~autophagosome organization	2	1.36986301369863	0.036290521428244786	GBA1, PIK3C2B	144	5	19416	53.93333333333333	1.0	0.5906602704872904	0.5765077340556005
GOTERM_BP_DIRECT	GO:0050856~regulation of T cell receptor signaling pathway	2	1.36986301369863	0.036290521428244786	PTPRC, PTPN22	144	5	19416	53.93333333333333	1.0	0.5906602704872904	0.5765077340556005
GOTERM_BP_DIRECT	GO:0042113~B cell activation	3	2.054794520547945	0.03663009429378545	BST2, CR2, PIK3CD	144	41	19416	9.865853658536587	1.0	0.5906602704872904	0.5765077340556005

GOTERM_BP_DIRECT GO:0032733~positive regulation of interleukin-10 production 3 2.054794520547945 0.03827960484196452 TNFSF4, ISG15, CD46 144 42 19416 9.630952380952381 1.0 0.603541769674974 0.5890805856235651
GOTERM_BP_DIRECT GO:2000648~positive regulation of stem cell proliferation 3 2.054794520547945 0.03827960484196452 PTPRC, KDM1A, HNRNPU 144 42 19416 9.630952380952381 1.0 0.603541769674974 0.5890805856235651
GOTERM_BP_DIRECT GO:0050727~regulation of inflammatory response 4 2.73972602739726 0.039617975496288815 IRF3, TNFSF4, NLRP3, PTGS2 144 102 19416 5.287581699346404 1.0 0.6042921014792207 0.5898129390759131
GOTERM_BP_DIRECT GO:0042307~positive regulation of protein import into nucleus 3 2.054794520547945 0.03995704975524075 ZC3H12A, PTPN22, PTGS2 144 43 19416 9.406976744186046 1.0 0.6042921014792207 0.5898129390759131
GOTERM_BP_DIRECT GO:0031295~T cell costimulation 3 2.054794520547945 0.03995704975524075 LCK, TNFRSF14, MTOR 144 43 19416 9.406976744186046 1.0 0.6042921014792207 0.5898129390759131
GOTERM_BP_DIRECT GO:0006397~mRNA processing 6 4.10958904109589 0.04003062546796811 IVNS1ABP, KHDRBS1, RNASEL, PNPT1, DDX1, ADAR 144 254 19416 3.18503937007874 1.0 0.6042921014792207 0.5898129390759131
GOTERM_BP_DIRECT GO:0048870~cell motility 3 2.054794520547945 0.04166193441811307 SKI, IGSF8, EPHA2 144 44 19416 9.193181818181817 1.0 0.6096609850206863 0.5950531812922133
GOTERM_BP_DIRECT GO:0034157~positive regulation of toll-like receptor 7 signaling pathway 2 1.36986301369863 0.043390127602747505 RSAD2, PTPN22 144 6 19416 44.94444444444444 1.0 0.6096609850206863 0.5950531812922133
GOTERM_BP_DIRECT GO:0141068~autosome genomic imprinting 2 1.36986301369863 0.043390127602747505 MYCN, DNMT3A 144 6 19416 44.94444444444444 1.0 0.6096609850206863 0.5950531812922133
GOTERM_BP_DIRECT GO:0002830~positive regulation of type 2 immune response 2 1.36986301369863 0.043390127602747505 TNFSF4, NLRP3 144 6 19416 44.94444444444444 1.0 0.6096609850206863 0.5950531812922133
GOTERM_BP_DIRECT GO:0006555~methionine metabolic process 2 1.36986301369863 0.043390127602747505 ADI1, MTHFR 144 6 19416 44.94444444444444 1.0 0.6096609850206863 0.5950531812922133
GOTERM_BP_DIRECT GO:0046641~positive regulation of alpha-beta T cell proliferation 2 1.36986301369863 0.043390127602747505 PTPRC, TNFSF4 144 6 19416 44.94444444444444 1.0 0.6096609850206863 0.5950531812922133
GOTERM_BP_DIRECT GO:0032722~positive regulation of chemokine production 3 2.054794520547945 0.043393769899287754 TNFSF4, EIF2AK2, IL6R 144 45 19416 8.988888888888889 1.0 0.6096609850206863 0.5950531812922133
GOTERM_BP_DIRECT GO:0034097~response to cytokine 3 2.054794520547945 0.04515207288134998 MAPKAPK2, REL, IL6R 144 46 19416 8.793478260869565 1.0 0.6281450139081924 0.6130943229477424
GOTERM_BP_DIRECT GO:0007169~cell surface receptor protein tyrosine kinase signaling pathway 4 2.73972602739726 0.04775771955287932 FGR, ALK, LCK, EPHA2 144 110 19416 4.903030303030302 1.0 0.6566167907576667 0.6408839007747487
GOTERM_BP_DIRECT GO:0042130~negative regulation of T cell proliferation 3 2.054794520547945 0.04874617590329995 IL10, CR1, PLA2G2A 144 48 19416 8.427083333333334 1.0 0.6566167907576667 0.6408839007747487

GOTERM_BP_DIRECT	GO:0002719~negative regulation of cytokine production involved in immune response	2	1.36986301369863	0.05043779435700188	IL10, APOA2	144	7	19416	38.523809523809526	1.0	0.6566167907576667	0.6408839007747487
GOTERM_BP_DIRECT	GO:0014902~myotube differentiation	2	1.36986301369863	0.05043779435700188	SKI, SORT1	144	7	19416	38.523809523809526	1.0	0.6566167907576667	0.6408839007747487
GOTERM_BP_DIRECT	GO:0034165~positive regulation of toll-like receptor 9 signaling pathway	2	1.36986301369863	0.05043779435700188	RSAD2, PTPN22	144	7	19416	38.523809523809526	1.0	0.6566167907576667	0.6408839007747487
GOTERM_BP_DIRECT	GO:0045630~positive regulation of T-helper 2 cell differentiation	2	1.36986301369863	0.05043779435700188	TNFSF4, NLRP3	144	7	19416	38.523809523809526	1.0	0.6566167907576667	0.6408839007747487
GOTERM_BP_DIRECT	GO:0090559~regulation of membrane permeability	2	1.36986301369863	0.05043779435700188	F11R, MTOR	144	7	19416	38.523809523809526	1.0	0.6566167907576667	0.6408839007747487
GOTERM_BP_DIRECT	GO:0006635~fatty acid beta-oxidation	3	2.054794520547945	0.052440487383021143	HADHB, CPT2, ACADM	144	50	19416	8.09	1.0	0.6703878522207838	0.6543250002295881
GOTERM_BP_DIRECT	GO:0034198~cellular response to amino acid starvation	3	2.054794520547945	0.052440487383021143	EIF2AK2, ATF3, MTOR	144	50	19416	8.09	1.0	0.6703878522207838	0.6543250002295881
GOTERM_BP_DIRECT	GO:0016477~cell migration	6	4.10958904109589	0.05299914212452987	SDC3, PIK3CD, SDC1, ARPC5, EPHA2, PIK3C2B	144	275	19416	2.941818181818182	1.0	0.671480202452749	0.6553911771649453
GOTERM_BP_DIRECT	GO:0007040~lysosome organization	3	2.054794520547945	0.05623133770847018	GBA1, PI4KB, MTOR	144	52	19416	7.778846153846154	1.0	0.6965701086580425	0.6798799157796962
GOTERM_BP_DIRECT	GO:0046854~phosphatidylinositol phosphate biosynthetic process	3	2.054794520547945	0.05623133770847018	PIK3CD, PI4KB, PIK3C2B	144	52	19416	7.778846153846154	1.0	0.6965701086580425	0.6798799157796962
GOTERM_BP_DIRECT	GO:0009086~methionine biosynthetic process	2	1.36986301369863	0.05743389902254473	ADI1, MTHFR	144	8	19416	33.70833333333333	1.0	0.6965701086580425	0.6798799157796962
GOTERM_BP_DIRECT	GO:0071636~positive regulation of transforming growth factor beta production	2	1.36986301369863	0.05743389902254473	CD46, PTGS2	144	8	19416	33.70833333333333	1.0	0.6965701086580425	0.6798799157796962
GOTERM_BP_DIRECT	GO:0002638~negative regulation of immunoglobulin production	2	1.36986301369863	0.05743389902254473	CR1, FCGR2B	144	8	19416	33.70833333333333	1.0	0.6965701086580425	0.6798799157796962
GOTERM_BP_DIRECT	GO:0032091~negative regulation of protein binding	3	2.054794520547945	0.05816184141929729	KDM1A, CFHR1, IFIT1	144	53	19416	7.632075471698113	1.0	0.6994207879151089	0.6826622912349724
GOTERM_BP_DIRECT	GO:0048661~positive regulation of smooth muscle cell proliferation	3	2.054794520547945	0.0601151420674639	HDAC1, PTGS2, IL6R	144	54	19416	7.4907407407407405	1.0	0.7168351814599266	0.6996594265835083
GOTERM_BP_DIRECT	GO:0002250~adaptive immune response	8	5.47945205479452	0.06097291933148282	PIK3CD, SLAMF7, TNFRSF14, CD247, CD46, CD1C, CD244, SLAMF1	144	473	19416	2.280479210711769	1.0	0.7210047710947843	0.7037291106175309

GOTERM_BP_DIRECT GO:0030968~endoplasmic reticulum unfolded protein response 3 2.054794520547945 0.06209080452264782 EIF2AK2, ATF6, ATF3 144 55 19416 7.354545454545454 1.0 0.722187308341289 0.704883313638256
GOTERM_BP_DIRECT GO:0097191~extrinsic apoptotic signaling pathway 3 2.054794520547945 0.06209080452264782 PTPRC, FASLG, IL6R 144 55 19416 7.354545454545454 1.0 0.722187308341289 0.704883313638256
GOTERM_BP_DIRECT GO:1903753~negative regulation of p38MAPK cascade 2 1.36986301369863 0.0643788161939623 DUSP10, PTPN22 144 9 19416 29.962962962962962 1.0 0.7250280966605754 0.7076560351479189
GOTERM_BP_DIRECT GO:0043382~positive regulation of memory T cell differentiation 2 1.36986301369863 0.0643788161939623 TNFSF4, CD46 144 9 19416 29.962962962962962 1.0 0.7250280966605754 0.7076560351479189
GOTERM_BP_DIRECT GO:2000627~positive regulation of miRNA catabolic process 2 1.36986301369863 0.0643788161939623 PNPT1, ZC3H12A 144 9 19416 29.962962962962962 1.0 0.7250280966605754 0.7076560351479189
GOTERM_BP_DIRECT GO:2000553~positive regulation of T-helper 2 cell cytokine production 2 1.36986301369863 0.0643788161939623 RSAD2, NLRP3 144 9 19416 29.962962962962962 1.0 0.7250280966605754 0.7076560351479189
GOTERM_BP_DIRECT GO:0070374~positive regulation of ERK1 and ERK2 cascade 5 3.4246575342465753 0.06518048758072495 PTPRC, PLA2G2A, CHI3L1, PTPN22, SLAMF1 144 205 19416 3.2886178861788617 1.0 0.7282764714728244 0.7108265771598744
GOTERM_BP_DIRECT GO:0072540~T-helper 17 cell lineage commitment 2 1.36986301369863 0.07127291776998251 SLAMF6, IL6R 144 10 19416 26.966666666666665 1.0 0.7712709704864794 0.7527909049498055
GOTERM_BP_DIRECT GO:0050764~regulation of phagocytosis 2 1.36986301369863 0.07127291776998251 FGR, PTPRC 144 10 19416 26.966666666666665 1.0 0.7712709704864794 0.7527909049498055
GOTERM_BP_DIRECT GO:0002526~acute inflammatory response 2 1.36986301369863 0.07127291776998251 TNFSF4, NLRP3 144 10 19416 26.966666666666665 1.0 0.7712709704864794 0.7527909049498055
GOTERM_BP_DIRECT GO:0140374~antiviral innate immune response 3 2.054794520547945 0.07228966812875388 IRF3, EIF2AK2, IFIT1 144 60 19416 6.741666666666666 1.0 0.7712709704864794 0.7527909049498055
GOTERM_BP_DIRECT GO:0010507~negative regulation of autophagy 3 2.054794520547945 0.07228966812875388 IL10, PTPN22, MTOR 144 60 19416 6.741666666666666 1.0 0.7712709704864794 0.7527909049498055
GOTERM_BP_DIRECT GO:0050853~B cell receptor signaling pathway 3 2.054794520547945 0.07228966812875388 PTPRC, LCK, PIK3CD 144 60 19416 6.741666666666666 1.0 0.7712709704864794 0.7527909049498055
GOTERM_BP_DIRECT GO:0038084~vascular endothelial growth factor signaling pathway 3 2.054794520547945 0.07651107842646716 ALK, PIK3CD, EPHA2 144 62 19416 6.524193548387096 1.0 0.798303090346742 0.7791753207401252
GOTERM_BP_DIRECT GO:1902895~positive regulation of miRNA transcription 3 2.054794520547945 0.07651107842646716 IL10, JUN, MYCN 144 62 19416 6.524193548387096 1.0 0.798303090346742 0.7791753207401252
GOTERM_BP_DIRECT GO:0042102~positive regulation of T cell proliferation 3 2.054794520547945 0.07651107842646716 PTPRC, TNFSF4, CD46 144 62 19416 6.524193548387096 1.0 0.798303090346742 0.7791753207401252

GOTERM_BP_DIRECT	GO:2000273~positive regulation of signaling receptor activity	2	1.36986301369863	0.07811657296664319	IL10, HDAC1	144	11	19416	24.515151515151512	1.0	0.8091052338661802	0.7897186391153345
GOTERM_BP_DIRECT	GO:0071230~cellular response to amino acid stimulus	3	2.054794520547945	0.08080873312442166	DNMT3A, MTOR, LAMTOR5	144	64	19416	6.3203125	1.0	0.830924581909814	0.8110151838936521
GOTERM_BP_DIRECT	GO:0010629~negative regulation of gene expression	6	4.10958904109589	0.0828685182953375	MYCN, HDAC1, ZC3H12A, REL, PTPN22, CD46	144	314	19416	2.5764331210191083	1.0	0.833584426984544	0.8136112976558093
GOTERM_BP_DIRECT	GO:0045893~positive regulation of DNA-templated transcription	10	6.8493150684931505	0.08329720686365089	IL10, JUN, MYCN, NR5A2, IRF3, ACTN2, HDAC1, IRF7, RORC, HIVEP3	144	717	19416	1.880520688052069	1.0	0.833584426984544	0.8136112976558093
GOTERM_BP_DIRECT	GO:0043508~negative regulation of JUN kinase activity	2	1.36986301369863	0.08491014833649205	DUSP10, PTPN22	144	12	19416	22.47222222222222	1.0	0.833584426984544	0.8136112976558093
GOTERM_BP_DIRECT	GO:0043031~negative regulation of macrophage activation	2	1.36986301369863	0.08491014833649205	ZC3H12A, FCGR2B	144	12	19416	22.47222222222222	1.0	0.833584426984544	0.8136112976558093
GOTERM_BP_DIRECT	GO:0031048~regulatory ncRNA-mediated heterochromatin formation	2	1.36986301369863	0.08491014833649205	DNMT3A, HNRNPU	144	12	19416	22.47222222222222	1.0	0.833584426984544	0.8136112976558093
GOTERM_BP_DIRECT	GO:0032731~positive regulation of interleukin-1 beta production	3	2.054794520547945	0.0851795221372508	AIM2, NLRP3, NLRC4	144	66	19416	6.128787878787879	1.0	0.833584426984544	0.8136112976558093
GOTERM_BP_DIRECT	GO:0030097~hemopoiesis	3	2.054794520547945	0.0851795221372508	IL10, MEIS1, LCK	144	66	19416	6.128787878787879	1.0	0.833584426984544	0.8136112976558093
GOTERM_BP_DIRECT	GO:0043433~negative regulation of DNA-binding transcription factor activity	3	2.054794520547945	0.08739139030221228	KDM1A, TNFSF4, TNFRSF4	144	67	19416	6.037313432835821	1.0	0.8390776583915518	0.818972908296194
GOTERM_BP_DIRECT	GO:0071456~cellular response to hypoxia	4	2.73972602739726	0.08756670684951162	LMNA, DNMT3A, PTGS2, MTOR	144	142	19416	3.7981220657276995	1.0	0.8390776583915518	0.818972908296194
GOTERM_BP_DIRECT	GO:0006915~apoptotic process	9	6.164383561643835	0.08800343823295098	IRF3, TNFRSF9, TRAF5, ZC3H12A, IFI6, NLRP3, CHI3L1, FASLG, NLRC4	144	620	19416	1.957258064516129	1.0	0.8390776583915518	0.818972908296194
GOTERM_BP_DIRECT	GO:1901731~positive regulation of platelet aggregation	2	1.36986301369863	0.0916540077876607	F11R, IL6R	144	13	19416	20.743589743589745	1.0	0.8390776583915518	0.818972908296194
GOTERM_BP_DIRECT	GO:0002726~positive regulation of T cell cytokine production	2	1.36986301369863	0.0916540077876607	TNFSF4, CD55	144	13	19416	20.743589743589745	1.0	0.8390776583915518	0.818972908296194
GOTERM_BP_DIRECT	GO:0044828~negative regulation by host of viral genome replication	2	1.36986301369863	0.0916540077876607	ZC3H12A, SMC6	144	13	19416	20.743589743589745	1.0	0.8390776583915518	0.818972908296194
GOTERM_BP_DIRECT	GO:0045793~positive regulation of cell size	2	1.36986301369863	0.0916540077876607	KDM1A, AKT3	144	13	19416	20.743589743589745	1.0	0.8390776583915518	0.818972908296194

GOTERM_BP_DIRECT	GO:0038083~peptidyl-tyrosine autophosphorylation	2	1.36986301369863	0.0916540077876607	ALK, LCK	144	13	19416	20.743589743589745	1.0	0.8390776583915518	0.818972908296194
GOTERM_BP_DIRECT	GO:0043922~negative regulation by host of viral transcription	2	1.36986301369863	0.0916540077876607	JUN, HDAC1	144	13	19416	20.743589743589745	1.0	0.8390776583915518	0.818972908296194
GOTERM_BP_DIRECT	GO:0002089~lens morphogenesis in camera-type eye	2	1.36986301369863	0.0916540077876607	SKI, MEIS1	144	13	19416	20.743589743589745	1.0	0.8390776583915518	0.818972908296194
GOTERM_BP_DIRECT	GO:0043065~positive regulation of apoptotic process	6	4.10958904109589	0.09446483465849823	JUN, DDX20, FASLG, NLRC4, PTGS2, ATF6	144	327	19416	2.474006116207951	1.0	0.8592666691051858	0.8386781795001285
GOTERM_BP_DIRECT	GO:0032922~circadian regulation of gene expression	3	2.054794520547945	0.09640670849909408	HDAC1, RORC, HNRNPU	144	71	19416	5.697183098591549	1.0	0.8668108036234141	0.8460415525147488
GOTERM_BP_DIRECT	GO:0046425~regulation of receptor signaling pathway via JAK-STAT	2	1.36986301369863	0.09834851260279752	PTPRC, IFNAR1	144	14	19416	19.261904761904763	1.0	0.8668108036234141	0.8460415525147488
GOTERM_BP_DIRECT	GO:0097284~hepatocyte apoptotic process	2	1.36986301369863	0.09834851260279752	DNMT3A, ADAR	144	14	19416	19.261904761904763	1.0	0.8668108036234141	0.8460415525147488
GOTERM_BP_DIRECT	GO:0030595~leukocyte chemotaxis	2	1.36986301369863	0.09834851260279752	IL10, LYST	144	14	19416	19.261904761904763	1.0	0.8668108036234141	0.8460415525147488
GOTERM_BP_DIRECT	GO:0034116~positive regulation of heterotypic cell-cell adhesion	2	1.36986301369863	0.09834851260279752	IL10, LCK	144	14	19416	19.261904761904763	1.0	0.8668108036234141	0.8460415525147488
GOTERM_BP_DIRECT	GO:0042127~regulation of cell population proliferation	4	2.73972602739726	0.09896775955369273	ALK, JUN, NR5A2, TNFRSF9	144	150	19416	3.5955555555555554	1.0	0.8668842642388271	0.8461132529744718

Table S3: list of cellular components

Category	Term	Count	%	PValue	Genes	List Total	Pop Hits	Pop Total	Fold Enrichment	Bonferroni	Benjamini	FDR
GOTERM_CC_DIRECT	GO:0009897~external side of plasma membrane	17	11.643835616438356	4.216893962835971E-8	TNFRSF9, FASLG, CD1C, FCGR3A, FCGR2A, PTPRC, SDC1, SLAMF7, CD48, SLAMF6, TNFRSF14, FCGR1A, FCGR2B, IL6R, TNFRSF4, CD244, SLAMF1	146	418	20666	5.756734613619978	1.1090369869348748E-5	1.1090431122258604E-5	1.0753079605231725E-5
GOTERM_CC_DIRECT	GO:0070062~extracellular exosome	36	24.65753424657534	3.979402688772525E-6	ALK, IGSF8, SLC2A1, GBA1, FASLG, F11R, NRAS, FCGR3A, PCBP1, SLAMF6, WASF2, CD55, CCT4, SLAMF1, ACTR2, CR2, CR1, SLC16A1, ACTN2, BROX, RPL22, PLA2G2A, APOA2, SYTL1, ARPC5, FGR, BST2, PTPRC, LCK, MAPKAPK2, MXRA8, CHI3L1, SDC1, CD48, NECTIN4, CD46	146	2242	20666	2.2728483619077875	0.0010460375104919262	5.232914535735871E-4	5.073738428184969E-4

GOTERM_CC_DIRECT	GO:0005829~cytosol	62	42.465753424657535	5.8656827402234606E-5	NCF2, SLC2A1, PIK3CD, HNRNPU, PTPN22, ADAR, IFIT1, LYST, PIK3C2B, RNF115, DUSP10, XPO1, FBXO6, LGALS8, ACTR2, BATF3, ACTN2, RPL22, APOA2, AMPD1, CTPS1, EML4, FGR, SIKE1, AIM2, IRF3, LCK, TNNT2, TRAF5, MAPKAPK2, IRF7, ATF6, FAM72A, KHDRBS1, RNASEL, HDAC1, DDX1, DDX20, NLRC4, NRAS, PCBP1, LMNA, NLRP3, EVI5, WASF2, CCT4, IVNS1ABP, EIF2B3, PNPT1, SORT1, MTHFR, EIF2AK2, ISG15, ARPC5, MTOR, MOV10, GALE, ADI1, POLR3C, REL, PI4KB, LAMTOR5	146	5576	20666	1.573883178396651	0.015308808435011256	0.0051422485355959	0.004985830329189942
GOTERM_CC_DIRECT	GO:0032991~protein-containing complex	16	10.95890410958904	1.0871068084245694E-4	ALK, KHDRBS1, KDM1A, HDAC1, HNRNPU, VRK2, F11R, PTGS2, SKI, XPO1, CFHR1, ZC3H12A, SDC1, RAD54L, WASF2, LAMTOR5	146	692	20666	3.2727848602422993	0.028187566937189268	0.007147727265391543	0.00693030590370663
GOTERM_CC_DIRECT	GO:0005654~nucleoplasm	47	32.19178082191781	2.5761636550129726E-4	KHDRBS1, KDM1A, HDAC1, DDX1, RORC, DDX20, HNRNPU, ADAR, PRDM2, SMC6, PIK3C2B, TGOLN2, DUSP10, XPO1, CPT2, PCBP1, AKT3, ZC3H12A, LMNA, RAD54L, CCT4, IVNS1ABP, TOE1, JUN, BATF3, DNMT3A, EIF2AK2, ISG15, GTF2H2, TENT5C, SDHB, MTOR, FGR, SKI, MYCL, MYCN, AIM2, NR5A2, IRF3, POLR3C, ZNF638, MAPKAPK2, REL, IRF7, CMPK2, ATF6, ATF3	146	4000	20666	1.6631883561643834	0.06551698970378006	0.013550620825368237	0.01313843464056616
GOTERM_CC_DIRECT	GO:0009986~cell surface	14	9.58904109589041	8.350528182783742E-4	CR1, SORT1, SDC3, HNRNPU, BST2, PTPRC, TNFSF4, MXRA8, SDC1, CD46, TNFRSF4, CD55, SLAMF1, EPHA2	146	665	20666	2.9799567411679884	0.1972489507740972	0.036603148534535404	0.0354897447768309
GOTERM_CC_DIRECT	GO:0005737~cytoplasm	60	41.0958904109589	0.0010316335279417978	PIK3CD, PTPN22, ADAR, IFIT1, LYST, IFI44L, PIK3C2B, RNF115, DUSP10, XPO1, AKT3, ZC3H12A, FBXO6, ACADM, LGALS8, ACTR2, ACTN2, RPL22, DNMT3A, CTPS1, VRK2, TENT5C, EML4, NR5A2, AIM2, IRF3, LCK, ZNF638, TRAF5, MAPKAPK2, IRF7, CMPK2, CHI3L1, XPR1, FAM72A, KHDRBS1, HDAC1, DDX1, DDX20, NLRC4, PTGS2, RNASEH1, PCBP1, NLRP3, HIVEP3, IVNS1ABP, TOE1, EIF2B3, PNPT1, IFI44, EIF2AK2, ISG15, ARPC5, MTOR, BST2, SKI, ADI1, REL, CD247, PI4KB	146	5887	20666	1.4426493481108829	0.23773397144349928	0.03875994540695612	0.03758093566073692
GOTERM_CC_DIRECT	GO:0090575~RNA polymerase II transcription regulator complex	6	4.10958904109589	0.0019635240338640543	BATF3, JUN, NR5A2, HNRNPU, ATF6, ATF3	146	126	20666	6.740378343118069	0.4036422105362878	0.06455085261328078	0.06258732857941673
GOTERM_CC_DIRECT	GO:0005635~nuclear envelope	7	4.794520547945205	0.003877127246878113	HAX1, XPO1, BROX, LMNA, VRK2, ATF6, MTOR	146	212	20666	4.673752907728095	0.6400054236657542	0.11329827399210486	0.10985193866154654
GOTERM_CC_DIRECT	GO:0016020~membrane	54	36.986301369863014	0.004838519628817005	ALK, NCF2, SLC2A1, HNRNPU, FASLG, ADAR, LYST, FCGR3A, XPO1, AKT3, LGALS8, IL6R, TNFRSF4, ACTR2, CR2, CR1, CTPS1, EML4, SLC5A6, MYCN, CD48, CD46, ATF6, IGSF8, KHDRBS1, DDX1, SDC3, DDX20, IFI6, TGOLN2, NRAS, PCBP1, SLAMF7, NLRP3, SLAMF6, TNFRSF14, FCGR1A, KIAA0319L, SLAMF1, SLC16A1, SORT1, TNFRSF9, EIF2AK2, MTOR, RPE65, BST2, PTPRC, FCGR2A, TNFSF4, SDC1, PI4KB, ACKR1, FCGR2B, CD244	146	5475	20666	1.3960893225745916	0.7207420050523091	0.12725306623788724	0.12338225053483362
GOTERM_CC_DIRECT	GO:0035861~site of double-strand break	5	3.4246575342465753	0.005417516147909608	ACTR2, AIM2, LMNA, SMC6, ARPC5	146	100	20666	7.0773972602739725	0.7603750292484944	0.12952788608183882	0.12558787433790455

GOTERM_CC_DIRECT	GO:0005886~plasma membrane	54	36.986301369863014	0.00609911915473969	ALK, NCF2, SLC2A1, PIK3CD, FASLG, F11R, PIK3C2B, FCGR3A, IL6R, TNFRSF4, CR2, CR1, ACTN2, SYTL1, SDHB, SLC5A6, FGR, LCK, CD48, CD46, XPR1, EPHA2, IFNAR1, IGSF8, SDC3, IFI6, NLRC4, CD1C, TGOLN2, NRAS, SLAMF7, SLAMF6, TNFRSF14, FCGR1A, KIAA0319L, CD55, SLAMF1, SLC16A1, SORT1, TNFRSF9, PLA2G2A, MTOR, RPE65, BST2, PTPRC, FCGR2A, ADI1, TNFSF4, SDC1, CD247, ACKR1, FCGR2B, NECTIN4, CD244	146	5534	20666	1.381205103197667	0.7999084007709988	0.13367236147471154	0.12960628203821842
GOTERM_CC_DIRECT	GO:0016363~nuclear matrix	5	3.4246575342465753	0.013709745118533638	RNASEL, GFI1, LMNA, DNMT3A, HNRNPU	146	131	20666	5.402593328453414	0.9734999213536951	0.277358689705719	0.26892192347892907
GOTERM_CC_DIRECT	GO:0045121~membrane raft	6	4.10958904109589	0.017631279067434613	BST2, PTPRC, LCK, CD48, CD244, CD55	146	214	20666	3.968633977723723	0.9907060505230632	0.3055694111150183	0.2962745240849037
GOTERM_CC_DIRECT	GO:0005783~endoplasmic reticulum	16	10.95890410958904	0.019426370950577032	RSAD2, PLA2G2A, GBA1, VRK2, PTGS2, CD1C, MTOR, PIK3C2B, HADHB, RNF115, ZDHHC18, HAX1, NLRP3, CHI3L1, SLAMF7, ATF6	146	1185	20666	1.911195884630946	0.9942549332851879	0.3055694111150183	0.2962745240849037
GOTERM_CC_DIRECT	GO:0033001~Fc-gamma receptor III complex	2	1.36986301369863	0.020902728154774453	FCGR3A, CD247	146	3	20666	94.36529680365297	0.9961345857903027	0.3055694111150183	0.2962745240849037
GOTERM_CC_DIRECT	GO:0036464~cytoplasmic ribonucleoprotein granule	4	2.73972602739726	0.021326755404519438	MOV10, PCBP1, ZC3H12A, HNRNPU	146	84	20666	6.740378343118068	0.9965507935588676	0.3055694111150183	0.2962745240849037
GOTERM_CC_DIRECT	GO:0005741~mitochondrial outer membrane	6	4.10958904109589	0.021740198106310716	HADHB, HAX1, RSAD2, PLA2G2A, PI4KB, MTOR	146	226	20666	3.757910049702994	0.9969135678671388	0.3055694111150183	0.2962745240849037
GOTERM_CC_DIRECT	GO:0005794~Golgi apparatus	15	10.273972602739725	0.023145408029106487	RSAD2, SORT1, GBA1, PRDM2, CD1C, BST2, TGOLN2, RNF115, ZDHHC18, NRAS, PI4KB, CD247, ATF6, KIAA0319L, XPR1	146	1103	20666	1.9249493908270094	0.9978851986376632	0.3055694111150183	0.2962745240849037
GOTERM_CC_DIRECT	GO:0005667~transcription regulator complex	6	4.10958904109589	0.023237217575286562	SKI, IVNS1ABP, MEIS1, JUN, HAX1, KDM1A	146	230	20666	3.692555092316855	0.9979368339742992	0.3055694111150183	0.2962745240849037
GOTERM_CC_DIRECT	GO:0043202~lysosomal lumen	4	2.73972602739726	0.03173252249001082	SDC3, SDC1, FASLG, GBA1	146	98	20666	5.777467151244059	0.999792615783128	0.3974120673748974	0.3853234873787028
GOTERM_CC_DIRECT	GO:0030667~secretory granule membrane	4	2.73972602739726	0.03425094499521661	CR1, FCGR2A, PTPRC, CD55	146	101	20666	5.605859216058591	0.9998954536781659	0.40945447880645314	0.39699958971728344
GOTERM_CC_DIRECT	GO:0000785~chromatin	14	9.58904109589041	0.05576114678712996	JUN, BATF3, KDM1A, HDAC1, RORC, MYCL, MEIS1, MYCN, NR5A2, IRF3, REL, IRF7, ATF6, ATF3	146	1134	20666	1.7475054963639438	0.9999997204040141	0.6376165915223991	0.6182214100312236
GOTERM_CC_DIRECT	GO:0030867~rough endoplasmic reticulum membrane	2	1.36986301369863	0.06800352646619207	ZC3H12A, PI4KB	146	10	20666	28.309589041095887	0.999999990964488	0.7196638088308169	0.6977728944937578

GOTERM_CC_DIRECT GO:0017053~transcription repressor complex 3 2.054794520547945 0.0684091073033096 SKI, HDAC1, GFI1 146 61 20666 6.961374354367842 0.9999999919418269 0.7196638088308169 0.6977728944937578
GOTERM_CC_DIRECT GO:0005764~lysosome 6 4.10958904109589 0.07447077886571365 SORT1, GBA1, CD1C, MTOR, LAMTOR5, IFNAR1 146 319 20666 2.6623437969682655 0.9999999985525435 0.7533005708339496 0.7303864850291147
GOTERM_CC_DIRECT GO:0005885~Arp2/3 protein complex 2 1.36986301369863 0.08104268352717507 ACTR2, ARPC5 146 12 20666 23.591324200913242 0.9999999997778356 0.7573545396991678 0.7343171392520448
GOTERM_CC_DIRECT GO:0016607~nuclear speck 7 4.794520547945205 0.08209650676543097 TOE1, ZNF638, PCBP1, LMNA, HNRNPU, GTF2H2, SMC6 146 429 20666 2.30964013155794 0.9999999998357078 0.7573545396991678 0.7343171392520448
GOTERM_CC_DIRECT GO:0005925~focal adhesion 7 4.794520547945205 0.08351057662082079 ACTR2, PTPRC, ACTN2, RPL22, ARPC5, CD46, EPHA2 146 431 20666 2.298922543940501 0.999999999890473 0.7573545396991678 0.7343171392520448
GOTERM_CC_DIRECT GO:0005634~nucleus 52 35.61643835616438 0.08901603135859408 KDM1A, GFI1, RORC, HNRNPU, PTPN22, FASLG, ADAR, PRDM2, SMC6, RNF115, DUSP10, XPO1, AKT3, ZC3H12A, ACADM, ACTR2, BATF3, RPL22, DNMT3A, VRK2, TENT5C, MYCN, NR5A2, IRF3, ZNF638, MAPKAPK2, MXRA8, IRF7, ATF6, ATF3, KHDRBS1, HDAC1, DDX1, DDX20, PCBP1, LMNA, NLRP3, RAD54L, HIVEP3, EVI5, JUN, EIF2AK2, ISG15, GTF2H2, ARPC5, MTOR, RPE65, SKI, MOV10, MEIS1, ADI1, REL 146 6138 20666 1.199167994572325 0.9999999999775445 0.7803738749103415 0.7566362665480497
GOTERM_CC_DIRECT GO:0005901~caveola 3 2.054794520547945 0.09746514946453874 SLC2A1, FASLG, PTGS2 146 75 20666 5.661917808219178 0.9999999999980634 0.8268817519088287 0.8017294552728188

Table S4: list of molecular functions

Category Term Count % PValue Genes List Total Pop Hits Pop Total Fold Enrichment Bonferroni Benjamini FDR
GOTERM_MF_DIRECT GO:0005515~protein binding 136 93.15068493150685 9.319638737432415E-12 KDM1A, NCF2, GFI1, RORC, HNRNPU, GBA1, SMC6, IFIT1, F11R, RNF115, AKT3, ZC3H12A, FBXO6, LGALS8, TNFRSF4, IL6R, RSAD2, ACTN2, BROX, RPL22, SYTL1, VRK2, SDHB, EML4, SLC5A6, MYCL, EBNA1BP2, MYCN, MAPKAPK2, MXRA8, ATF6, ATF3, IFNAR1, EPHA2, IGSF8, KHDRBS1, DDX1, SDC3, CD1C, TGOLN2, RNASEH1, PCBP1, NLRP3, TNFRSF14, HIVEP3, SLAMF6, KIAA0319L, EVI5, SLAMF1, TOE1, IVNS1ABP, JUN, EIF2B3, PNPT1, TNFRSF9, IFI44, MTHFR, EIF2AK2, ISG15, ARPC5, RPE65, BST2, MOV10, PTPRC, ADI1, TNFSF4, REL, SDC1, PI4KB, ALK, SLC2A1, PIK3CD, PTPN22, FASLG, ADAR, LYST, PIK3C2B, FCGR3A, XPO1, DUSP10, CPT2, IL10, ACTR2, CR2, BATF3, CR1, DNMT3A, APOA2, AMPD1, CTPS1, TENT5C, HADHB, FGR, SIKE1, NR5A2, HAX1, AIM2, IRF3, LCK, ZNF638, TNNT2, TRAF5, IRF7, CHI3L1, CD48, CD46, FAM72A, RNASEL, HDAC1, DDX20, IFI6, NLRC4, PTGS2, NRAS, LMNA, RAD54L, FCGR1A, WASF2, CD55, CCT4, SLC16A1, SORT1, GTF2H2, MTOR, AGMAT, SKI, MEIS1, FCGR2A, CFHR1, POLR3C, CD247, ACKR1, FCGR2B, NECTIN4, CD244, LAMTOR5 145 13670 19208 1.3179063138511211 4.473434955798439E-9 4.47342659396756E-9 4.408189122805533E-9

GOTERM_MF_DIRECT GO:0001618~virus receptor activity 11 7.534246575342466 5.524424576360906E-10 CR2, CR1, TNFRSF14, F11R, CD46, NECTIN4, TNFRSF4, CD55, XPR1, EPHA2, SLAMF1 145 82 19208 17.77022708158116 2.6517232898370935E-7 1.3258618983266175E-7 1.3065264123093543E-7
GOTERM_MF_DIRECT GO:0042802~identical protein binding 37 25.34246575342466 2.3130331663018294E-8 ALK, KHDRBS1, RNASEL, KDM1A, SDC3, SLC2A1, HNRNPU, NLRC4, LMNA, SLAMF7, NLRP3, ACADM, SLAMF1, JUN, PNPT1, SLC16A1, ACTN2, RPL22, DNMT3A, AMPD1, EIF2AK2, CTPS1, MTOR, SKI, BST2, GALE, AIM2, IRF3, CFHR1, LCK, TNNT2, TRAF5, SDC1, CD247, NECTIN4, ATF6, ATF3 145 1777 19208 2.7582170647934334 1.1102497687209834E-5 3.7008530660829273E-6 3.646882292202551E-6
GOTERM_MF_DIRECT GO:0019770~IgG receptor activity 4 2.73972602739726 2.2480925772149812E-5 FCGR3A, FCGR2A, FCGR1A, FCGR2B 145 8 19208 66.23448275862069 0.010732952058337464 0.0026977110926579774 0.0026583694725567154
GOTERM_MF_DIRECT GO:0019864~IgG binding 4 2.73972602739726 8.639277518636252E-5 FCGR3A, FCGR2A, FCGR1A, FCGR2B 145 12 19208 44.156321839080455 0.04062219408963541 0.008293706417890803 0.008172756532629895
GOTERM_MF_DIRECT GO:0005524~ATP binding 25 17.123287671232877 4.8632459403121013E-4 ALK, RNASEL, DDX1, DDX20, PIK3CD, HNRNPU, NLRC4, SMC6, PIK3C2B, AKT3, NLRP3, RAD54L, CCT4, ACTR2, EIF2AK2, CTPS1, VRK2, MTOR, FGR, MOV10, LCK, MAPKAPK2, CMPK2, PI4KB, EPHA2 145 1544 19208 2.1448990530641416 0.20823653440980316 0.03890596752249681 0.038338588829460396
GOTERM_MF_DIRECT GO:0016301~kinase activity 8 5.47945205479452 0.0016009915910363682 FGR, ALK, CMPK2, EIF2AK2, PIK3CD, PI4KB, MTOR, EPHA2 145 226 19208 4.689166920964297 0.5365662242587224 0.1097822805282081 0.10818128893717174
GOTERM_MF_DIRECT GO:0003700~DNA-binding transcription factor activity 13 8.904109589041095 0.0018443631048285594 JUN, BATF3, GFI1, RORC, PRDM2, MYCL, MYCN, NR5A2, IRF3, REL, IRF7, ATF6, ATF3 145 598 19208 2.87976011994003 0.5877443851401812 0.11066178628971356 0.10904796857298857
GOTERM_MF_DIRECT GO:0035591~signaling adaptor activity 5 3.4246575342465753 0.0032245158017659916 KHDRBS1, AIM2, HAX1, TRAF5, NLRP3 145 81 19208 8.177096636866752 0.7878089779655314 0.1554677348986488 0.1532004970980435
GOTERM_MF_DIRECT GO:0003723~RNA binding 22 15.068493150684931 0.00323891114372185 KHDRBS1, JUN, RNASEL, PNPT1, DDX1, RPL22, DDX20, EIF2AK2, HNRNPU, ADAR, IFIT1, TENT5C, BST2, HADHB, MOV10, EBNA1BP2, RNASEH1, XPO1, ZNF638, PCBP1, ZC3H12A, CCT4 145 1473 19208 1.9784909988997357 0.7892748348957961 0.1554677348986488 0.1532004970980435
GOTERM_MF_DIRECT GO:0001227~DNA-binding transcription repressor activity, RNA polymerase II-specific 8 5.47945205479452 0.005978263634532078 SKI, BATF3, JUN, IRF3, GFI1, RORC, PRDM2, ATF3 145 287 19208 3.692514718250631 0.9437628887229831 0.26086968587049064 0.2570653362848794
GOTERM_MF_DIRECT GO:0000978~RNA polymerase II cis-regulatory region sequence-specific DNA binding 18 12.32876712328767 0.008389215256895484 JUN, BATF3, HDAC1, GFI1, DNMT3A, RORC, HNRNPU, SKI, MYCL, MEIS1, MYCN, NR5A2, IRF3, REL, IRF7, HIVEP3, ATF6, ATF3 145 1198 19208 1.990351735651373 0.982469434706834 0.31135666699587766 0.3068160489355211

GOTERM_MF_DIRECT	GO:1990837~sequence-specific double-stranded DNA binding	11	7.534246575342466	0.009891085623249092	BATF3, JUN, MYCN, NR5A2, IRF3, GFI1, IRF7, RORC, HNRNPU, ATF6, ATF3	145	562	19208	2.592808933611486	0.9915311211282422	0.31135666699587766	0.3068160489355211
GOTERM_MF_DIRECT	GO:0000976~transcription cis-regulatory region binding	7	4.794520547945205	0.010104043703721588	JUN, NR5A2, IRF3, GFI1, IRF7, ATF6, ATF3	145	243	19208	3.8159784305378173	0.9923619221116142	0.31135666699587766	0.3068160489355211
GOTERM_MF_DIRECT	GO:0004713~protein tyrosine kinase activity	5	3.4246575342465753	0.010388096629827	FGR, ALK, LCK, MTOR, EPHA2	145	113	19208	5.86145865120537	0.9933448624906712	0.31135666699587766	0.3068160489355211
GOTERM_MF_DIRECT	GO:0035401~histone H3Y41 kinase activity	5	3.4246575342465753	0.011027215289437333	FGR, ALK, LCK, EIF2AK2, EPHA2	145	115	19208	5.75952023988006	0.9951192849741884	0.31135666699587766	0.3068160489355211
GOTERM_MF_DIRECT	GO:0140801~histone H2AXY142 kinase activity	5	3.4246575342465753	0.011027215289437333	FGR, ALK, LCK, EIF2AK2, EPHA2	145	115	19208	5.75952023988006	0.9951192849741884	0.31135666699587766	0.3068160489355211
GOTERM_MF_DIRECT	GO:0004540~RNA nuclease activity	3	2.054794520547945	0.011686622043573834	RNASEL, RNASEH1, ZC3H12A	145	22	19208	18.063949843260186	0.9964564092609247	0.3116432544953023	0.3070984570339124
GOTERM_MF_DIRECT	GO:0003677~DNA binding	19	13.013698630136986	0.012752043561494092	KHDRBS1, CR2, JUN, DDX1, DNMT3A, RORC, DDX20, HNRNPU, ADAR, MYCL, MEIS1, MYCN, NR5A2, IRF3, ZC3H12A, REL, IRF7, ATF6, ATF3	145	1355	19208	1.8574984094668536	0.9978884719727165	0.3221568899745876	0.3174587686624582
GOTERM_MF_DIRECT	GO:0004888~transmembrane signaling receptor activity	6	4.10958904109589	0.01459233649239515	CR2, FCGR3A, ACKR1, CD247, FCGR2B, SLAMF1	145	191	19208	4.161328759703918	0.999137718597857	0.34143855639732335	0.33645924411652905
GOTERM_MF_DIRECT	GO:0019772~low-affinity IgG receptor activity	2	1.36986301369863	0.014937936842382895	FCGR3A, FCGR2B	145	2	19208	132.46896551724137	0.9992713398537139	0.34143855639732335	0.33645924411652905
GOTERM_MF_DIRECT	GO:1990841~promoter-specific chromatin binding	4	2.73972602739726	0.015669296613160848	KDM1A, IRF3, HDAC1, HNRNPU	145	70	19208	7.5696551724137935	0.9994898530329321	0.34187556246896394	0.3368898771829582
GOTERM_MF_DIRECT	GO:0003725~double-stranded RNA binding	4	2.73972602739726	0.01751792024678252	DDX1, HNRNPU, EIF2AK2, ADAR	145	73	19208	7.258573452999528	0.9997930687455896	0.36559137906328737	0.36025983811861445
GOTERM_MF_DIRECT	GO:0008143~poly(A) binding	3	2.054794520547945	0.019851128145029876	KHDRBS1, DDX1, HNRNPU	145	29	19208	13.703686087990487	0.9999339030677764	0.3970225629005975	0.3912326505249638
GOTERM_MF_DIRECT	GO:0019899~enzyme binding	8	5.47945205479452	0.022819709489065292	JUN, KDM1A, SORT1, HDAC1, APOA2, PTGS2, IL6R, ATF6	145	374	19208	2.833560759727088	0.9999845881273313	0.41721137933283375	0.4111270467175632
GOTERM_MF_DIRECT	GO:0106222~lncRNA binding	3	2.054794520547945	0.02390064445112802	HADHB, DNMT3A, HNRNPU	145	32	19208	12.418965517241379	0.9999909399102828	0.41721137933283375	0.4111270467175632
GOTERM_MF_DIRECT	GO:0005164~tumor necrosis factor receptor binding	3	2.054794520547945	0.02390064445112802	TNFSF4, TRAF5, FASLG	145	32	19208	12.418965517241379	0.9999909399102828	0.41721137933283375	0.4111270467175632

GOTERM_MF_DIRECT	GO:0019904~protein domain specific binding	6	4.10958904109589	0.02433733046108197	SKI, KHDRBS1, IRF3, ACTN2, DDX20, VRK2	145	218	19208	3.6459348307497628	0.9999926911260333	0.41721137933283375	0.4111270467175632
GOTERM_MF_DIRECT	GO:0038023~signaling receptor activity	6	4.10958904109589	0.027400998337715955	IGSF8, TNFRSF9, ACKR1, CD46, CD244, SLAMF1	145	225	19208	3.5325057471264367	0.9999983847738633	0.45353376558978137	0.4469197315082637
GOTERM_MF_DIRECT	GO:0000822~inositol hexakisphosphate binding	2	1.36986301369863	0.029654258314743165	MTOR, XPR1	145	4	19208	66.23448275862069	0.999999469455186	0.46368482484411333	0.45692275448180336
GOTERM_MF_DIRECT	GO:0031625~ubiquitin protein ligase binding	7	4.794520547945205	0.029946311604515653	SKI, JUN, TRAF5, ISG15, TNFRSF14, PTPN22, SMC6	145	311	19208	2.98161658720479	0.9999995408334664	0.46368482484411333	0.45692275448180336
GOTERM_MF_DIRECT	GO:0001228~DNA-binding transcription activator activity, RNA polymerase II-specific	9	6.164383561643835	0.033187715273472446	MEIS1, JUN, MYCN, NR5A2, IRF3, REL, PRDM2, ATF6, ATF3	145	495	19208	2.408526645768025	0.9999999079048312	0.4978157291020867	0.4905559163860146
GOTERM_MF_DIRECT	GO:0042169~SH2 domain binding	3	2.054794520547945	0.03952858094949203	KHDRBS1, LCK, SLAMF1	145	42	19208	9.462068965517242	0.9999999960867817	0.5740973945312987	0.5657251408610506
GOTERM_MF_DIRECT	GO:0140297~DNA-binding transcription factor binding	5	3.4246575342465753	0.04066523211263366	SKI, KDM1A, HDAC1, DDX20, NLRP3	145	172	19208	3.85084202085004	0.9999999977833942	0.5740973945312987	0.5657251408610506
GOTERM_MF_DIRECT	GO:0004715~non-membrane spanning protein tyrosine kinase activity	3	2.054794520547945	0.046609773116146225	FGR, LCK, EIF2AK2	145	46	19208	8.63928035982009	0.9999999998878152	0.6316349684865757	0.6224236251961465
GOTERM_MF_DIRECT	GO:0003697~single-stranded DNA binding	4	2.73972602739726	0.048767679598889446	POLR3C, PCBP1, HNRNPU, SMC6	145	109	19208	4.861246440999683	0.9999999999621935	0.6316349684865757	0.6224236251961465
GOTERM_MF_DIRECT	GO:0003690~double-stranded DNA binding	4	2.73972602739726	0.048767679598889446	JUN, AIM2, ZNF638, HNRNPU	145	109	19208	4.861246440999683	0.9999999999621935	0.6316349684865757	0.6224236251961465
GOTERM_MF_DIRECT	GO:0035005~1-phosphatidylinositol-4-phosphate 3-kinase activity	2	1.36986301369863	0.051320341189534276	PIK3CD, PIK3C2B	145	7	19208	37.84827586206897	0.9999999999895911	0.6316349684865757	0.6224236251961465
GOTERM_MF_DIRECT	GO:0140608~cysteine-type endopeptidase activator activity	2	1.36986301369863	0.051320341189534276	AIM2, NLRP3	145	7	19208	37.84827586206897	0.9999999999895911	0.6316349684865757	0.6224236251961465
GOTERM_MF_DIRECT	GO:0042803~protein homodimerization activity	11	7.534246575342466	0.05717168856511408	BST2, CR2, GALE, IRF3, APOA2, NLRC4, CD247, F11R, PTGS2, IL6R, ATF3	145	753	19208	1.9351376104776299	0.9999999999994659	0.6836986593964121	0.6737280539468811
GOTERM_MF_DIRECT	GO:0000981~DNA-binding transcription factor activity, RNA polymerase II-specific	16	10.95890410958904	0.0583992604901102	JUN, BATF3, RORC, PRDM2, SKI, MYCL, MEIS1, MYCN, NR5A2, IRF3, PCBP1, REL, IRF7, HIVEP3, ATF6, ATF3	145	1285	19208	1.6494190258956125	0.9999999999997142	0.6836986593964121	0.6737280539468811

GOTERM_MF_DIRECT GO:1990782~protein tyrosine kinase binding 3 2.054794520547945 0.06404781254693412 KHDRBS1, PTPN22, CD247 145 55 19208 7.225579937304075 0.9999999999999841 0.7249532383582326 0.714381003632175
GOTERM_MF_DIRECT GO:0005102~signaling receptor binding 7 4.794520547945205 0.06879229198109568 FGR, PTPRC, LCK, TNFSF4, APOA2, FASLG, GBA1 145 383 19208 2.421103808409111 0.9999999999999986 0.7249532383582326 0.714381003632175
GOTERM_MF_DIRECT GO:0016303~1-phosphatidylinositol-3-kinase activity 2 1.36986301369863 0.07250594408549718 PIK3CD, PIK3C2B 145 10 19208 26.493793103448276 0.9999999999999998 0.7249532383582326 0.714381003632175
GOTERM_MF_DIRECT GO:0005031~tumor necrosis factor receptor activity 2 1.36986301369863 0.07250594408549718 TNFRSF14, TNFRSF4 145 10 19208 26.493793103448276 0.9999999999999998 0.7249532383582326 0.714381003632175
GOTERM_MF_DIRECT GO:0034046~poly(G) binding 2 1.36986301369863 0.07250594408549718 PNPT1, HNRNPU 145 10 19208 26.493793103448276 0.9999999999999998 0.7249532383582326 0.714381003632175
GOTERM_MF_DIRECT GO:0001851~complement component C3b binding 2 1.36986301369863 0.0794628741807901 CR1, CFHR1 145 11 19208 24.085266457680248 1.0 0.7249532383582326 0.714381003632175
GOTERM_MF_DIRECT GO:0017069~snRNA binding 2 1.36986301369863 0.0794628741807901 TOE1, HNRNPU 145 11 19208 24.085266457680248 1.0 0.7249532383582326 0.714381003632175
GOTERM_MF_DIRECT GO:0050660~flavin adenine dinucleotide binding 3 2.054794520547945 0.08553109928787034 KDM1A, MTHFR, ACADM 145 65 19208 6.11395225464191 1.0 0.7249532383582326 0.714381003632175

Table S5: list of keg pathway

Category Term Count % PValue Genes List Total Pop Hits Pop Total Fold Enrichment Bonferroni Benjamini FDR
KEGG_PATHWAY hsa05135:Yersinia infection 12 8.21917808219178 8.305272495107214E-7 IL10, ACTR2, JUN, FCGR2A, IRF3, LCK, AKT3, NLRP3, PIK3CD, NLRC4, ARPC5, WASF2 109 138 8865 7.072197846031114 2.0428891840307273E-4 1.0761812705394359E-4 8.880682842256322E-5
KEGG_PATHWAY hsa05162:Measles 12 8.21917808219178 8.928141487693778E-7 JUN, IRF3, AKT3, IRF7, EIF2AK2, PIK3CD, FASLG, ADAR, CD46, FCGR2B, SLAMF1, IFNAR1 109 139 8865 7.021318724836645 2.1960826120615007E-4 1.0761812705394359E-4 8.880682842256322E-5
KEGG_PATHWAY hsa05235:PD-L1 expression and PD-1 checkpoint pathway in cancer 10 6.8493150684931505 1.312416183584678E-6 EML4, ALK, BATF3, NRAS, JUN, LCK, AKT3, PIK3CD, CD247, MTOR 109 90 8865 9.036697247706423 3.2280248109428644E-4 1.0761812705394359E-4 8.880682842256322E-5
KEGG_PATHWAY hsa04666:Fc gamma R-mediated phagocytosis 10 6.8493150684931505 2.9371210086099473E-6 ACTR2, FCGR3A, FCGR2A, PTPRC, AKT3, PIK3CD, ARPC5, FCGR1A, FCGR2B, WASF2 109 99 8865 8.215179316096748 7.222718652170057E-4 1.8063294202951176E-4 1.4905889118695483E-4
KEGG_PATHWAY hsa05164:Influenza A 12 8.21917808219178 7.619662766236703E-6 RNASEL, XPO1, RSAD2, IRF3, AKT3, IRF7, NLRP3, EIF2AK2, PIK3CD, FASLG, ADAR, IFNAR1 109 173 8865 5.641406374290714 0.0018726885084544564 3.748874080988458E-4 3.0935830830921017E-4

KEGG_PATHWAY hsa05160:Hepatitis C 11 7.534246575342466 2.2235149882553512E-5 RNASEL, NRAS, RSAD2, IRF3, AKT3, IRF7, EIF2AK2, PIK3CD, FASLG, IFIT1, IFNAR1 109 159 8865 5.626622814609659 0.005454974977651128 9.11641145184694E-4 7.522892376930605E-4
KEGG_PATHWAY hsa05169:Epstein-Barr virus infection 12 8.21917808219178 3.587139272232059E-5 CR2, JUN, IRF3, HDAC1, AKT3, TRAF5, IRF7, EIF2AK2, PIK3CD, ISG15, CD247, IFNAR1 109 204 8865 4.784133837021047 0.008785699078681874 0.001260623229955838 0.0010402703889472972
KEGG_PATHWAY hsa05140:Leishmaniasis 8 5.47945205479452 4.618374098005523E-5 IL10, FCGR3A, JUN, CR1, FCGR2A, NCF2, FCGR1A, PTGS2 109 79 8865 8.23597723841598 0.01129716495948574 0.0014201500351366983 0.0011719124273689013
KEGG_PATHWAY hsa04380:Osteoclast differentiation 10 6.8493150684931505 5.798836055328165E-5 FCGR3A, JUN, FCGR2A, NCF2, LCK, AKT3, PIK3CD, FCGR1A, FCGR2B, IFNAR1 109 143 8865 5.687431834220826 0.014164279488404707 0.001585015188456365 0.0013079596880351306
KEGG_PATHWAY hsa05167:Kaposi sarcoma-associated herpesvirus infection 11 7.534246575342466 1.3094412372324888E-4 NRAS, JUN, IRF3, AKT3, MAPKAPK2, IRF7, EIF2AK2, PIK3CD, PTGS2, MTOR, IFNAR1 109 196 8865 4.5644542220557955 0.031701008249707896 0.0032212254435919226 0.0026581657115819523
KEGG_PATHWAY hsa04650:Natural killer cell mediated cytotoxicity 9 6.164383561643835 1.8724924651439715E-4 FCGR3A, NRAS, LCK, PIK3CD, CD48, FASLG, CD247, CD244, IFNAR1 109 131 8865 5.5875761607955745 0.045022623051777955 0.003879669430297915 0.003201515830692995
KEGG_PATHWAY hsa05203:Viral carcinogenesis 11 7.534246575342466 1.892521673316056E-4 NRAS, JUN, IRF3, HDAC1, TRAF5, MAPKAPK2, IRF7, REL, EIF2AK2, PIK3CD, GTF2H2 109 205 8865 4.36406354889237 0.04549313074252459 0.003879669430297915 0.003201515830692995
KEGG_PATHWAY hsa04625:C-type lectin receptor signaling pathway 8 5.47945205479452 2.7987593490024973E-4 IL10, NRAS, JUN, AKT3, MAPKAPK2, NLRP3, PIK3CD, PTGS2 109 105 8865 6.196592398427261 0.06654182027341549 0.0052961138450354946 0.004370370368057746
KEGG_PATHWAY hsa03265:Virion - Ebolavirus, Lyssavirus and Morbillivirus 4 2.73972602739726 3.571329916064536E-4 CD46, FCGR2B, NECTIN4, SLAMF1 109 12 8865 27.110091743119266 0.08412043991851137 0.0062753368525133996 0.0051784283782935776
KEGG_PATHWAY hsa04623:Cytosolic DNA-sensing pathway 7 4.794520547945205 5.018423175299222E-4 AIM2, IRF3, POLR3C, PCBP1, IRF7, NLRP3, ADAR 109 83 8865 6.859179838620538 0.11616439205630735 0.008230214007490725 0.006791599363904948
KEGG_PATHWAY hsa05171:Coronavirus disease - COVID-19 11 7.534246575342466 6.222238599958333E-4 JUN, FCGR2A, IRF3, RPL22, NLRP3, EIF2AK2, PIK3CD, ISG15, ADAR, IL6R, IFNAR1 109 238 8865 3.7589623005165373 0.1419687088724767 0.009566691847435938 0.007894465223697135
KEGG_PATHWAY hsa04660:T cell receptor signaling pathway 8 5.47945205479452 6.955763507491866E-4 IL10, NRAS, JUN, PTPRC, LCK, AKT3, PIK3CD, CD247 109 122 8865 5.333132801925102 0.15732280681538424 0.010065398957899993 0.008305999953063817
KEGG_PATHWAY hsa05131:Shigellosis 11 7.534246575342466 9.09804035363436E-4 ACTR2, JUN, IRF3, AKT3, TRAF5, NLRP3, PIK3CD, NLRC4, ARPC5, WASF2, MTOR 109 250 8865 3.5785321100917433 0.20061585532274706 0.012433988848330029 0.010260567732154306
KEGG_PATHWAY hsa05170:Human immunodeficiency virus 1 infection 10 6.8493150684931505 0.001125826244446173 BST2, NRAS, JUN, IRF3, AKT3, TRAF5, PIK3CD, FASLG, CD247, MTOR 109 213 8865 3.818322780721023 0.24202833390938883 0.014576487164934662 0.012028564611714376

KEGG_PATHWAY	hsa05417:Lipid and atherosclerosis	10	6.8493150684931505	0.0012418643270072837	NRAS, JUN, IRF3, NCF2, AKT3, IRF7, NLRP3, PIK3CD, FASLG, ATF6	109	216	8865	3.7652905198776763	0.26338394237976426	0.015274931222189592	0.01260492291912393
KEGG_PATHWAY	hsa04621:NOD-like receptor signaling pathway	9	6.164383561643835	0.002104428948847478	RNASEL, JUN, AIM2, IRF3, TRAF5, IRF7, NLRP3, NLRC4, IFNAR1	109	189	8865	3.8728702490170384	0.40442920100241964	0.02465188197221331	0.020342813172192283
KEGG_PATHWAY	hsa04613:Neutrophil extracellular trap formation	9	6.164383561643835	0.002398347749684589	FCGR3A, CR1, FCGR2A, NCF2, HDAC1, AKT3, PIK3CD, FCGR1A, MTOR	109	193	8865	3.7926035081047678	0.4460618186859534	0.02681788847374586	0.022130208781180525
KEGG_PATHWAY	hsa04722:Neurotrophin signaling pathway	7	4.794520547945205	0.003380553598813365	NRAS, JUN, SORT1, AKT3, MAPKAPK2, PIK3CD, FASLG	109	120	8865	4.744266055045872	0.5652677796510963	0.036157225448177725	0.02983706002430926
KEGG_PATHWAY	hsa05161:Hepatitis B	8	5.47945205479452	0.003681954818417742	NRAS, JUN, IRF3, AKT3, IRF7, PIK3CD, FASLG, IFNAR1	109	163	8865	3.9916699499071315	0.5964409570912583	0.03774003688878185	0.03114320117245007
KEGG_PATHWAY	hsa04662:B cell receptor signaling pathway	6	4.10958904109589	0.004959946097059796	CR2, NRAS, JUN, AKT3, PIK3CD, FCGR2B	109	91	8865	5.362435729408206	0.7057079725289047	0.04880586959506839	0.04027476230812554
KEGG_PATHWAY	hsa04370:VEGF signaling pathway	5	3.4246575342465753	0.006019546266897303	NRAS, AKT3, MAPKAPK2, PIK3CD, PTGS2	109	60	8865	6.7775229357798175	0.7735618060625503	0.056802915802418724	0.04687395084508537
KEGG_PATHWAY	hsa05163:Human cytomegalovirus infection	9	6.164383561643835	0.006234466368558153	NRAS, IRF3, AKT3, TRAF5, PIK3CD, FASLG, PTGS2, IL6R, MTOR	109	226	8865	3.2388162701956644	0.7852926895123153	0.056802915802418724	0.04687395084508537
KEGG_PATHWAY	hsa04213:Longevity regulating pathway - multiple species	5	3.4246575342465753	0.00675991636836147	NRAS, HDAC1, AKT3, PIK3CD, MTOR	109	62	8865	6.558893163657887	0.8114864802050529	0.058474528913278104	0.04825337142030673
KEGG_PATHWAY	hsa05231:Choline metabolism in cancer	6	4.10958904109589	0.007067380394380596	NRAS, JUN, AKT3, PIK3CD, WASF2, MTOR	109	99	8865	4.929107589658049	0.8253110412147722	0.058474528913278104	0.04825337142030673
KEGG_PATHWAY	hsa05165:Human papillomavirus infection	11	7.534246575342466	0.007247236536065223	NRAS, IRF3, HDAC1, AKT3, EIF2AK2, PIK3CD, FASLG, ISG15, PTGS2, MTOR, IFNAR1	109	333	8865	2.6865856682370444	0.8329248939774391	0.058474528913278104	0.04825337142030673
KEGG_PATHWAY	hsa04640:Hematopoietic cell lineage	6	4.10958904109589	0.0073687414484212245	CR2, CR1, FCGR1A, CD1C, IL6R, CD55	109	100	8865	4.879816513761469	0.8378805848733286	0.058474528913278104	0.04825337142030673
KEGG_PATHWAY	hsa05142:Chagas disease	6	4.10958904109589	0.008326545402376114	IL10, JUN, AKT3, PIK3CD, FASLG, CD247	109	103	8865	4.737685935690745	0.8721510207830837	0.06401031778076638	0.05282152239632348
KEGG_PATHWAY	hsa05221:Acute myeloid leukemia	5	3.4246575342465753	0.00933619335215015	NRAS, AKT3, PIK3CD, FCGR1A, MTOR	109	68	8865	5.980167296276309	0.90048948878266	0.0695970777160284	0.05743173486322668
KEGG_PATHWAY	hsa05211:Renal cell carcinoma	5	3.4246575342465753	0.010319114487606458	NRAS, JUN, AKT3, SLC2A1, PIK3CD	109	70	8865	5.809305373525556	0.9220502209287951	0.07171757503196706	0.05918157614426551

KEGG_PATHWAY	hsa04620:Toll-like receptor signaling pathway	6	4.10958904109589	0.010495254882726887	JUN, IRF3, AKT3, IRF7, PIK3CD, IFNAR1	109	109	8865	4.476895884184834	0.9253897025967472	0.07171757503196706	0.05918157614426551
KEGG_PATHWAY	hsa04659:Th17 cell differentiation	6	4.10958904109589	0.010495254882726887	JUN, LCK, RORC, CD247, IL6R, MTOR	109	109	8865	4.476895884184834	0.9253897025967472	0.07171757503196706	0.05918157614426551
KEGG_PATHWAY	hsa05230:Central carbon metabolism in cancer	5	3.4246575342465753	0.010834768148699657	NRAS, AKT3, SLC2A1, PIK3CD, MTOR	109	71	8865	5.727484171081535	0.9314298079868772	0.07189143749290967	0.059325048012441726
KEGG_PATHWAY	hsa04622:RIG-I-like receptor signaling pathway	5	3.4246575342465753	0.011366803949220601	SIKE1, IRF3, IRF7, ISG15, ADAR	109	72	8865	5.647935779816514	0.9399301577693729	0.07189143749290967	0.059325048012441726
KEGG_PATHWAY	hsa05132:Salmonella infection	9	6.164383561643835	0.011397423017168607	ACTR2, JUN, PTPRC, AKT3, NLRP3, PIK3CD, NLRC4, ARPC5, PIK3C2B	109	251	8865	2.916225008223985	0.9403860914804698	0.07189143749290967	0.059325048012441726
KEGG_PATHWAY	hsa05223:Non-small cell lung cancer	5	3.4246575342465753	0.011915405443862055	EML4, ALK, NRAS, AKT3, PIK3CD	109	73	8865	5.570566796531356	0.9475970749838247	0.07327974347975165	0.060470682627599935
KEGG_PATHWAY	hsa05200:Pathways in cancer	14	9.58904109589041	0.012512616632601482	ALK, JUN, HDAC1, SLC2A1, PIK3CD, FASLG, PTGS2, MTOR, EML4, NRAS, AKT3, TRAF5, IL6R, IFNAR1	109	533	8865	2.136254884073188	0.9548390914674287	0.0750756997956089	0.06195271161995368
KEGG_PATHWAY	hsa01521:EGFR tyrosine kinase inhibitor resistance	5	3.4246575342465753	0.01623376607473659	NRAS, AKT3, PIK3CD, IL6R, MTOR	109	80	8865	5.083142201834862	0.9821593624690261	0.09416169113199509	0.07770253373900407
KEGG_PATHWAY	hsa04919:Thyroid hormone signaling pathway	6	4.10958904109589	0.01645915739299101	NRAS, HDAC1, AKT3, SLC2A1, PIK3CD, MTOR	109	122	8865	3.9998496014438265	0.983137180209455	0.09416169113199509	0.07770253373900407
KEGG_PATHWAY	hsa04012:ErbB signaling pathway	5	3.4246575342465753	0.02062857857280343	NRAS, JUN, AKT3, PIK3CD, MTOR	109	86	8865	4.728504373799872	0.9940698316342874	0.11533250747521918	0.09517276023361583
KEGG_PATHWAY	hsa05210:Colorectal cancer	5	3.4246575342465753	0.021425399552130426	NRAS, JUN, AKT3, PIK3CD, MTOR	109	87	8865	4.674153748813667	0.9951458964617248	0.11712551755164632	0.09665235797961058
KEGG_PATHWAY	hsa04610:Complement and coagulation cascades	5	3.4246575342465753	0.022240900682968995	CR2, CR1, CFHR1, CD46, CD55	109	88	8865	4.621038365304421	0.9960459777040372	0.1189404688697907	0.09815006170962404
KEGG_PATHWAY	hsa05144:Malaria	4	2.73972602739726	0.02277787211483233	IL10, CR1, SDC1, ACKR1	109	50	8865	6.5064220183486245	0.9965457804521874	0.11922035192018623	0.0983810221129992
KEGG_PATHWAY	hsa05152:Tuberculosis	7	4.794520547945205	0.02354907910458894	IL10, FCGR3A, CR1, FCGR2A, AKT3, FCGR1A, FCGR2B	109	182	8865	3.1280875088214537	0.9971555182124059	0.1206890304110183	0.09959298037982406
KEGG_PATHWAY	hsa05168:Herpes simplex virus 1 infection	13	8.904109589041095	0.02494554910802146	RNASEL, EIF2B3, EIF2AK2, PIK3CD, FASLG, MTOR, BST2, IRF3, AKT3, TRAF5, IRF7, TNFRSF14, IFNAR1	109	523	8865	2.021593839353062	0.9979996693135121	0.12316912193902013	0.10163955997406945

KEGG_PATHWAY	hsa04210:Apoptosis	6	4.10958904109589	0.02503437437784962	NRAS, JUN, AKT3, LMNA, PIK3CD, FASLG	109	136	8865	3.588100377765785	0.9980440002535061	0.12316912193902013	0.10163955997406945
KEGG_PATHWAY	hsa01522:Endocrine resistance	5	3.4246575342465753	0.03246863999975019	NRAS, JUN, AKT3, PIK3CD, MTOR	109	99	8865	4.107589658048374	0.9997024209122025	0.15661343999879504	0.12923791999900566
KEGG_PATHWAY	hsa05150:Staphylococcus aureus infection	5	3.4246575342465753	0.03566481600339276	IL10, FCGR3A, FCGR2A, FCGR1A, FCGR2B	109	102	8865	3.986778197517539	0.9998681466867879	0.16872201416989652	0.13922995478247557
KEGG_PATHWAY	hsa05130:Pathogenic Escherichia coli infection	7	4.794520547945205	0.03737124431471292	ACTR2, JUN, FCGR2A, NLRP3, FASLG, ARPC5, WASF2	109	203	8865	2.8044922492882	0.9999147150545148	0.17345898304564866	0.14313891690352307
KEGG_PATHWAY	hsa05415:Diabetic cardiomyopathy	7	4.794520547945205	0.038910528575842734	CPT2, NCF2, AKT3, SLC2A1, PIK3CD, SDHB, MTOR	109	205	8865	2.7771313492951446	0.999942469303134	0.17725907462328355	0.1462747648314088
KEGG_PATHWAY	hsa04932:Non-alcoholic fatty liver disease	6	4.10958904109589	0.0425468339258505	JUN, AKT3, PIK3CD, FASLG, IL6R, SDHB	109	157	8865	3.1081633845614447	0.999977358002339	0.186902163317129	0.15423227298120806
KEGG_PATHWAY	hsa04148:Efferocytosis	6	4.10958904109589	0.0425468339258505	IL10, SLC16A1, DNMT3A, MAPKAPK2, SLC2A1, PTGS2	109	157	8865	3.1081633845614447	0.999977358002339	0.186902163317129	0.15423227298120806
KEGG_PATHWAY	hsa04066:HIF-1 signaling pathway	5	3.4246575342465753	0.043809598082963815	AKT3, SLC2A1, PIK3CD, IL6R, MTOR	109	109	8865	3.730746570154028	0.9999836349445513	0.1890730022527912	0.15602365633055534
KEGG_PATHWAY	hsa04630:JAK-STAT signaling pathway	6	4.10958904109589	0.05407816448911953	IL10, AKT3, PIK3CD, IL6R, MTOR, IFNAR1	109	168	8865	2.9046526867627787	0.9999988508103945	0.2293660080055759	0.18927357571191836
KEGG_PATHWAY	hsa05166:Human T-cell leukemia virus 1 infection	7	4.794520547945205	0.05556210384220087	NRAS, JUN, XPO1, LCK, AKT3, SLC2A1, PIK3CD	109	224	8865	2.541571100917431	0.9999992189880971	0.23166572110476977	0.19117130644011487
KEGG_PATHWAY	hsa04668:TNF signaling pathway	5	3.4246575342465753	0.05711374669294454	JUN, AKT3, TRAF5, PIK3CD, PTGS2	109	119	8865	3.417238455015034	0.9999994788187369	0.2341663614410726	0.1932348429777957
KEGG_PATHWAY	hsa05214:Glioma	4	2.73972602739726	0.06527425814525975	NRAS, AKT3, PIK3CD, MTOR	109	76	8865	4.280540801545148	0.9999999385794132	0.263237172192359	0.21722417054897916
KEGG_PATHWAY	hsa05220:Chronic myeloid leukemia	4	2.73972602739726	0.06734163528708677	NRAS, HDAC1, AKT3, PIK3CD	109	77	8865	4.2249493625640415	0.9999999643748215	0.2653162125559607	0.2189398014181302
KEGG_PATHWAY	hsa05100:Bacterial invasion of epithelial cells	4	2.73972602739726	0.06943878253316975	ACTR2, PIK3CD, ARPC5, WASF2	109	78	8865	4.1707833450952725	0.999999979523422	0.2653162125559607	0.2189398014181302
KEGG_PATHWAY	hsa05133:Pertussis	4	2.73972602739726	0.06943878253316975	IL10, JUN, IRF3, NLRP3	109	78	8865	4.1707833450952725	0.999999979523422	0.2653162125559607	0.2189398014181302
KEGG_PATHWAY	hsa04014:Ras signaling pathway	7	4.794520547945205	0.0702296852505627	NRAS, AKT3, PLA2G2A, REL, PIK3CD, FASLG, EPHA2	109	238	8865	2.3920669185105234	0.999999983388214	0.2653162125559607	0.2189398014181302

KEGG_PATHWAY hsa04060:Cytokine-cytokine receptor interaction 8 5.47945205479452 0.0711823984906236 IL10, TNFRSF9, TNFSF4, FASLG, TNFRSF14, IL6R, TNFRSF4, IFNAR1 109 298 8865 2.1833630934055783 0.9999999870911525 0.2653162125559607 0.2189398014181302
KEGG_PATHWAY hsa04068:FoxO signaling pathway 5 3.4246575342465753 0.0789758877801211 IL10, NRAS, AKT3, PIK3CD, FASLG 109 133 8865 3.05752914396082 0.9999999983757009 0.2899711700583551 0.23928515252782961
KEGG_PATHWAY hsa05418:Fluid shear stress and atherosclerosis 5 3.4246575342465753 0.09309598598049582 JUN, NCF2, AKT3, PIK3CD, SDC1 109 141 8865 2.8840523130977944 0.999999999963685 0.3367884198706172 0.2779188993241272
KEGG_PATHWAY hsa00071:Fatty acid degradation 3 2.054794520547945 0.09623933863633448 HADHB, CPT2, ACADM 109 43 8865 5.674205248559847 0.9999999999845423 0.3402242551510958 0.2807541617710262
KEGG_PATHWAY hsa04211:Longevity regulating pathway 4 2.73972602739726 0.09681177992104352 NRAS, AKT3, PIK3CD, MTOR 109 90 8865 3.6146788990825693 0.9999999999867732 0.3402242551510958 0.2807541617710262

Table S6: targets with different topological properties

Item	Occurences	Present In
FASLG	6	MCC, Degree, EPC, BottleNeck, Closeness, Radiality
FCGR3A	6	MCC, Degree, EPC, BottleNeck, Closeness, Radiality
IL10	6	MCC, Degree, EPC, BottleNeck, Closeness, Radiality
PTPRC	6	MCC, Degree, EPC, BottleNeck, Closeness, Radiality
JUN	5	Degree, EPC, BottleNeck, Closeness, Radiality
MTOR	5	MCC, Degree, EPC, Closeness, Radiality
IRF7	4	Degree, EPC, Closeness, Radiality
LCK	4	Degree, EPC, Closeness, Radiality

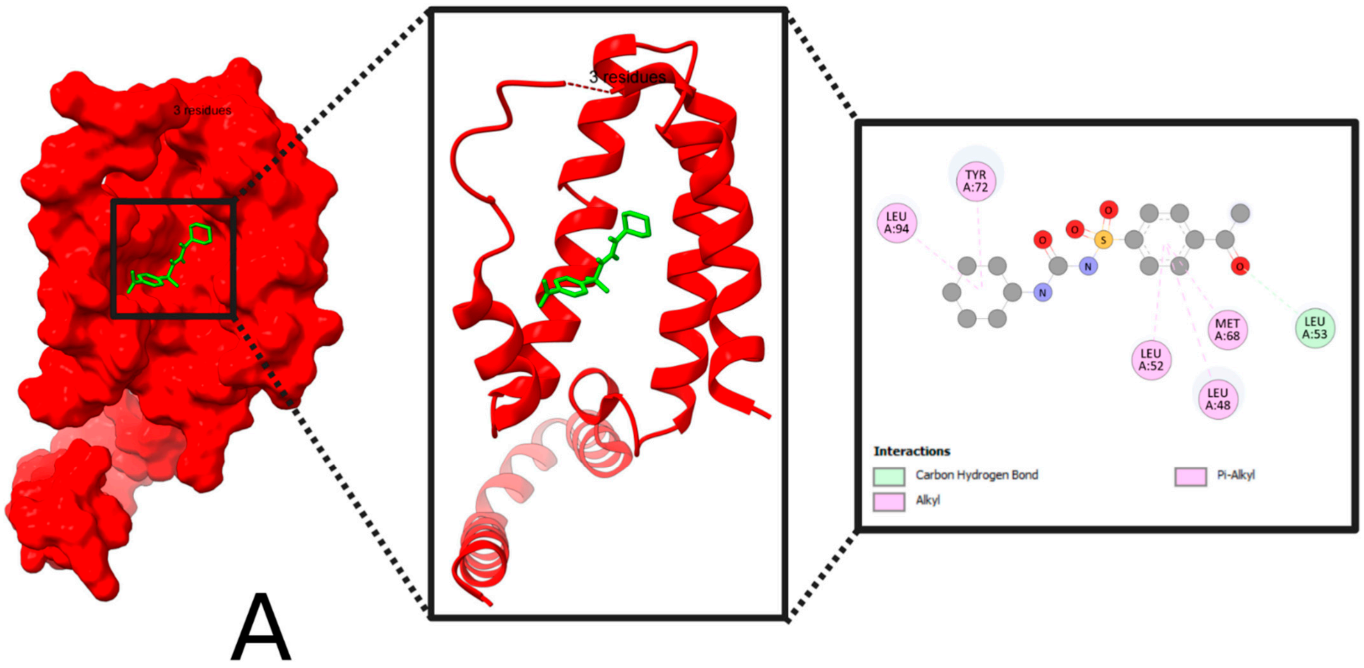

Figure S1: Molecular interaction of IL10 with the selected compound (IL10 + Acetohexamide)

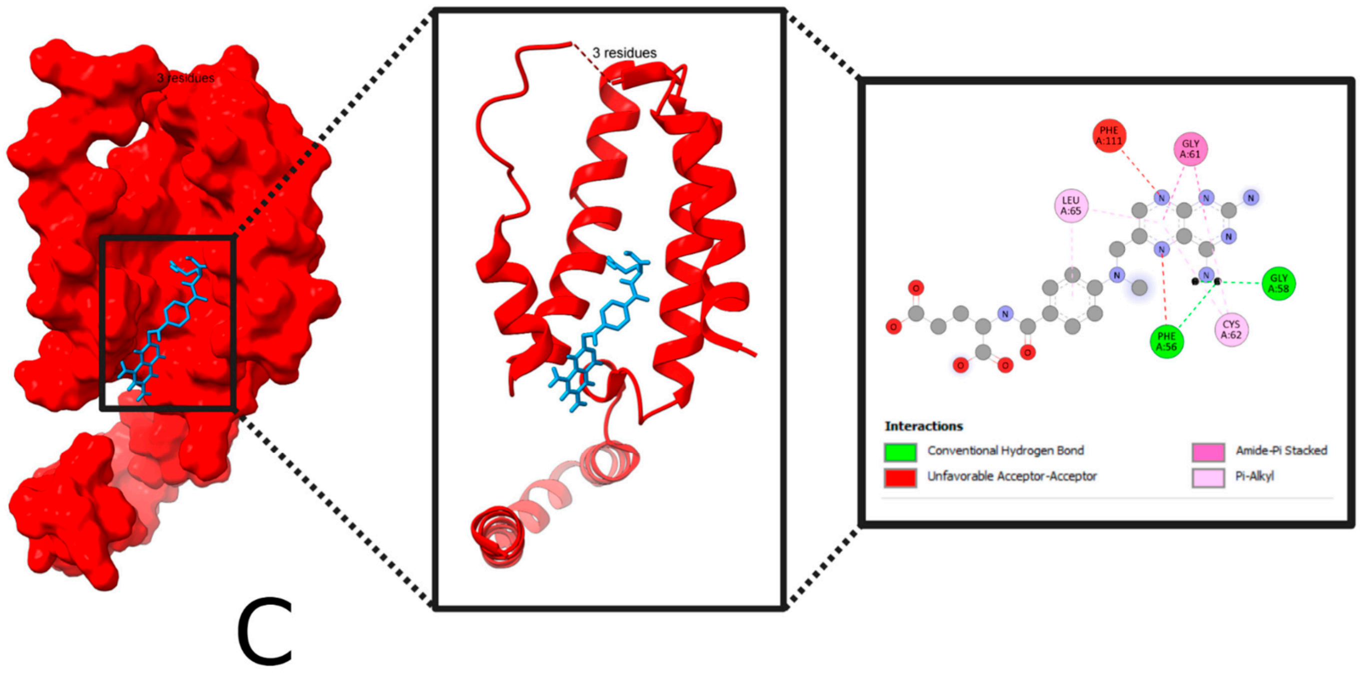

Figure S2: Molecular interaction of IL10 with the selected compound (IL10 + Methotrexate)

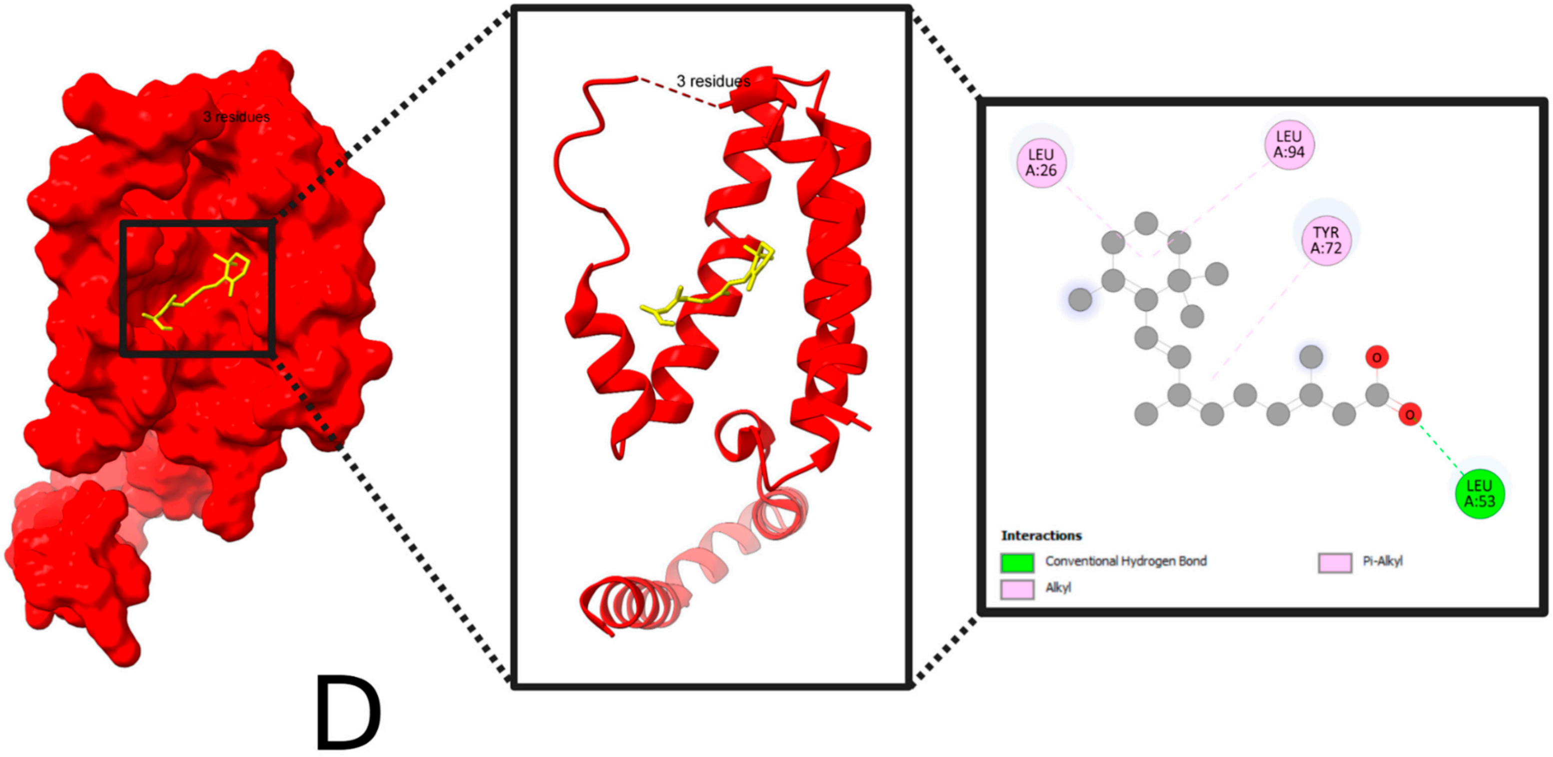

Figure S3: Molecular interaction of IL10 with the selected compound (IL10 + Retinoic acid)

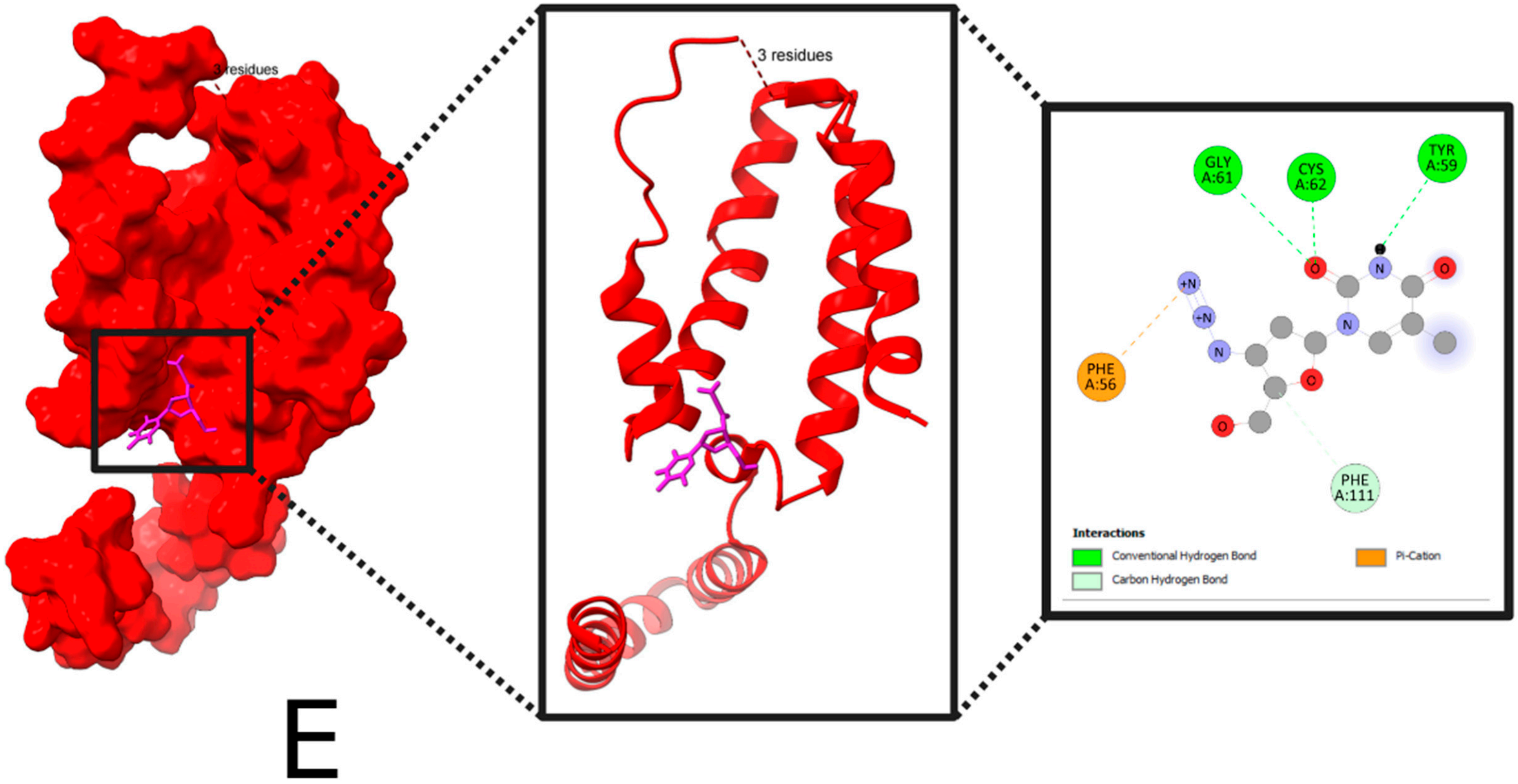

Figure S4: Molecular interaction of IL10 with the selected compound (IL10 + 3-Azido-3-deoxythymidine)

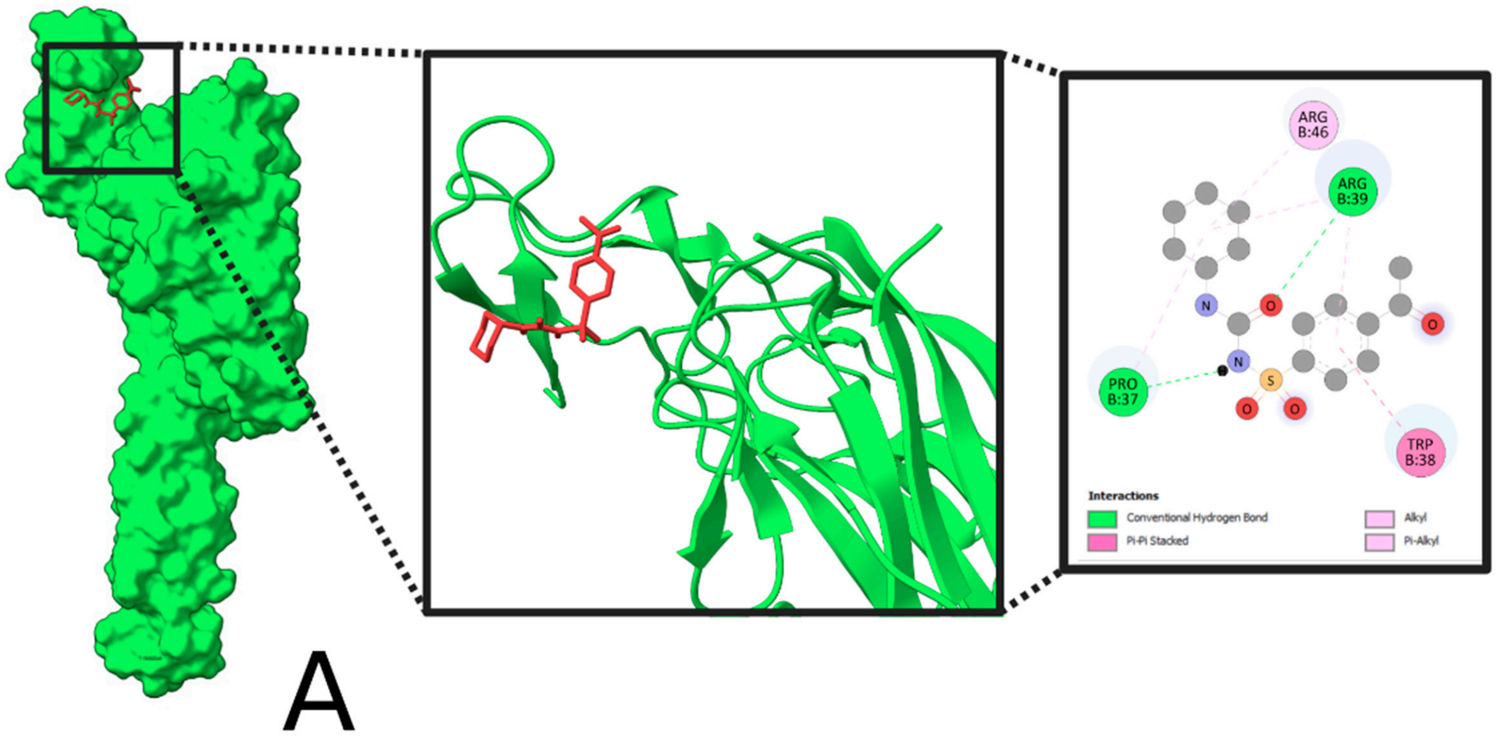

Figure S5: Molecular interaction of FASLG with the selected compounds (FASLG + Acetohexamide)

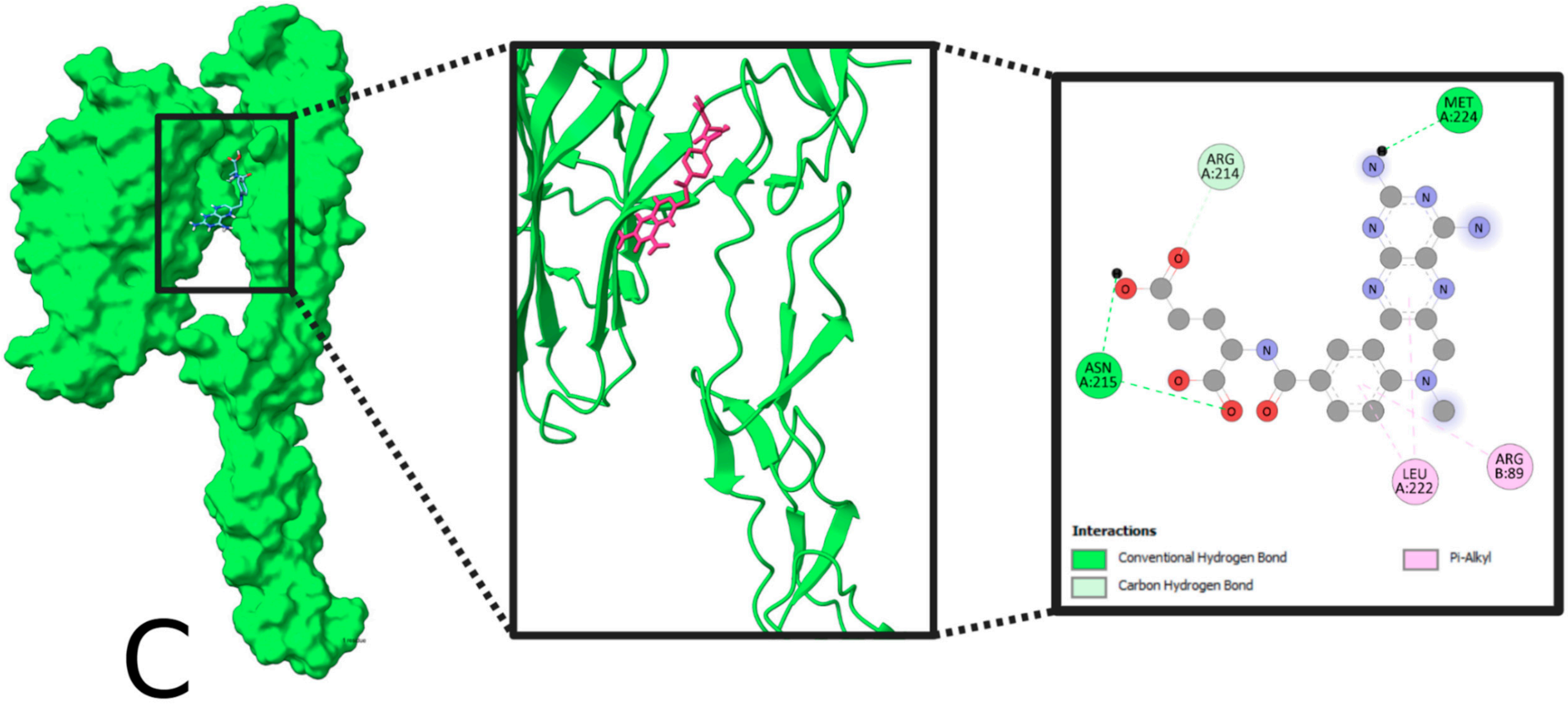

Figure S6: Molecular interaction of FASLG with the selected compounds (FASLG + Methotrexate)

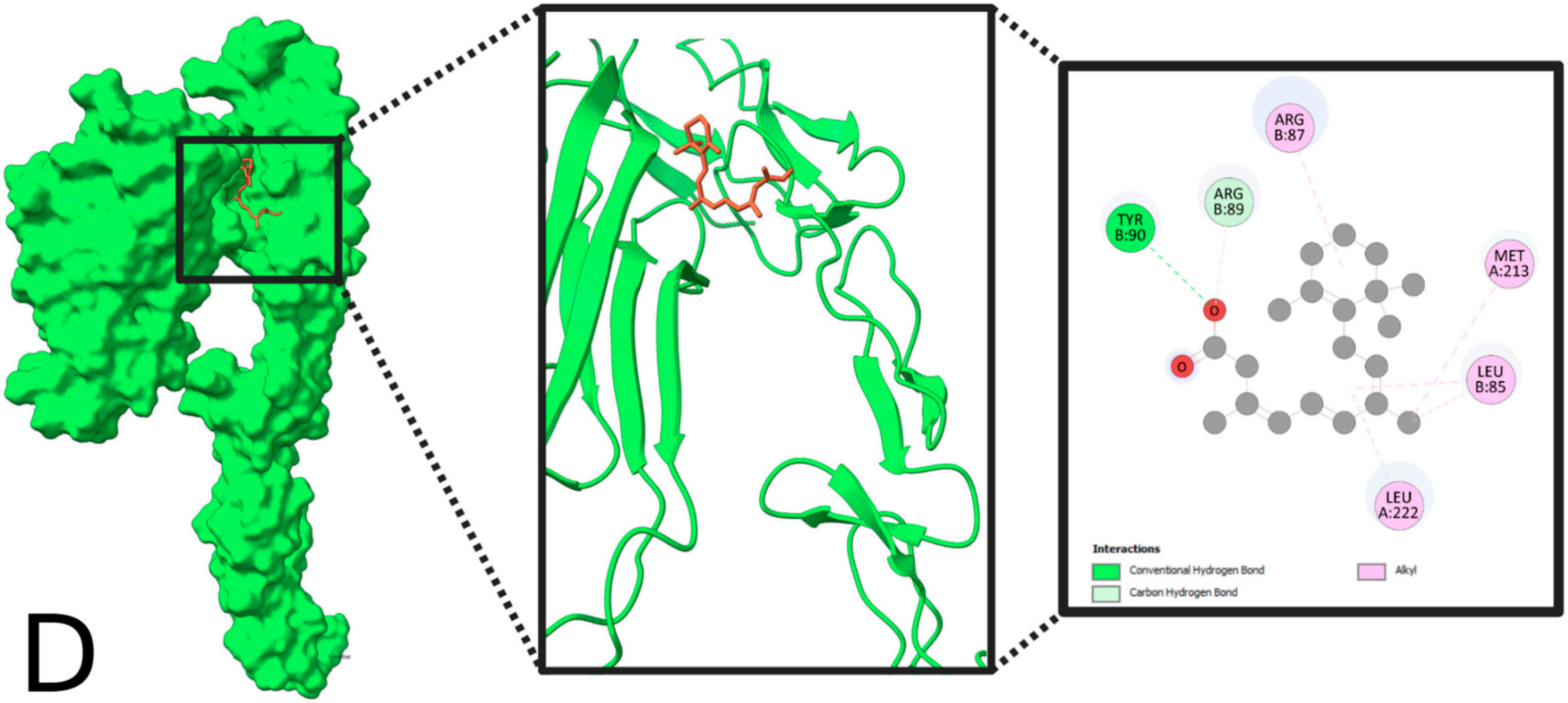

Figure S7: Molecular interaction of FASLG with the selected compounds (FASLG + Retinoic acid)

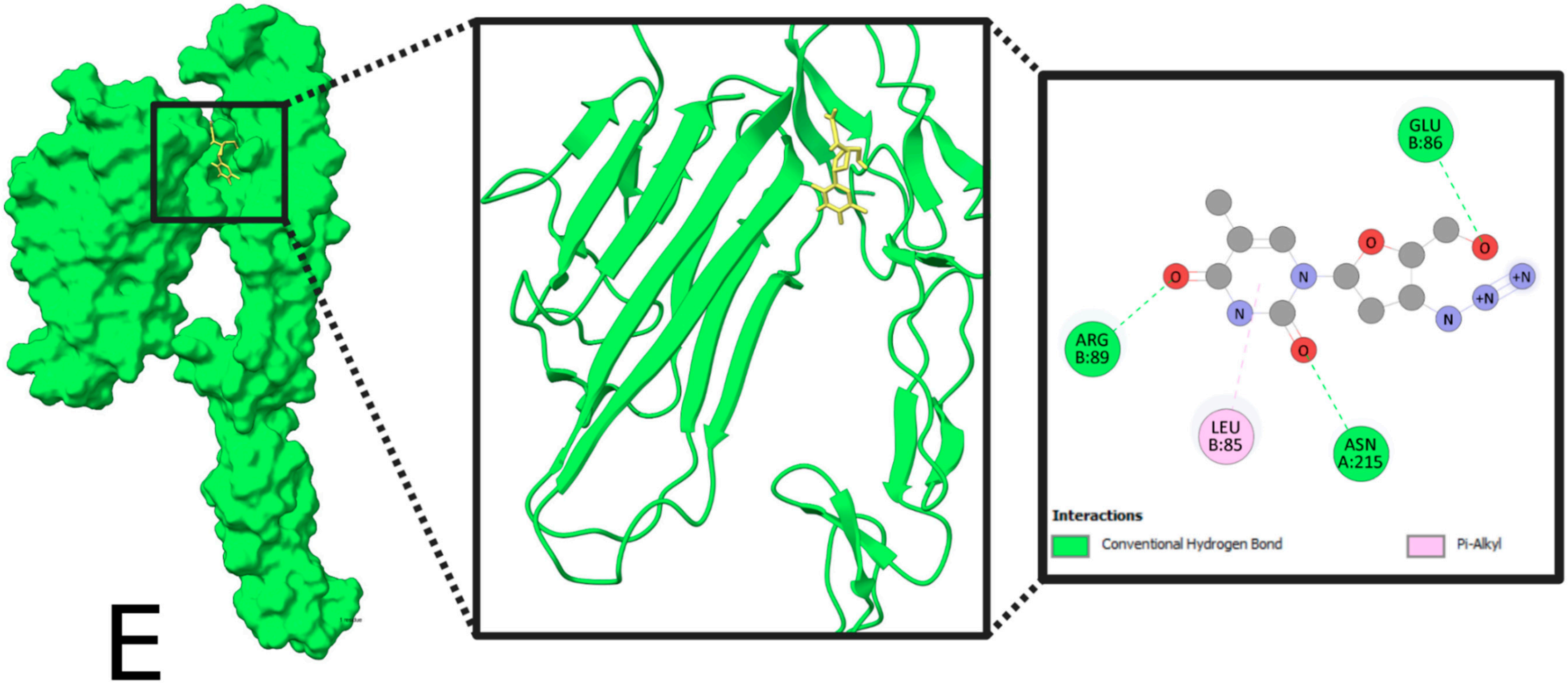

Figure S8: Molecular interaction of FASLG with the selected compounds (FASLG + 3-Azido-3-deoxythymidine)

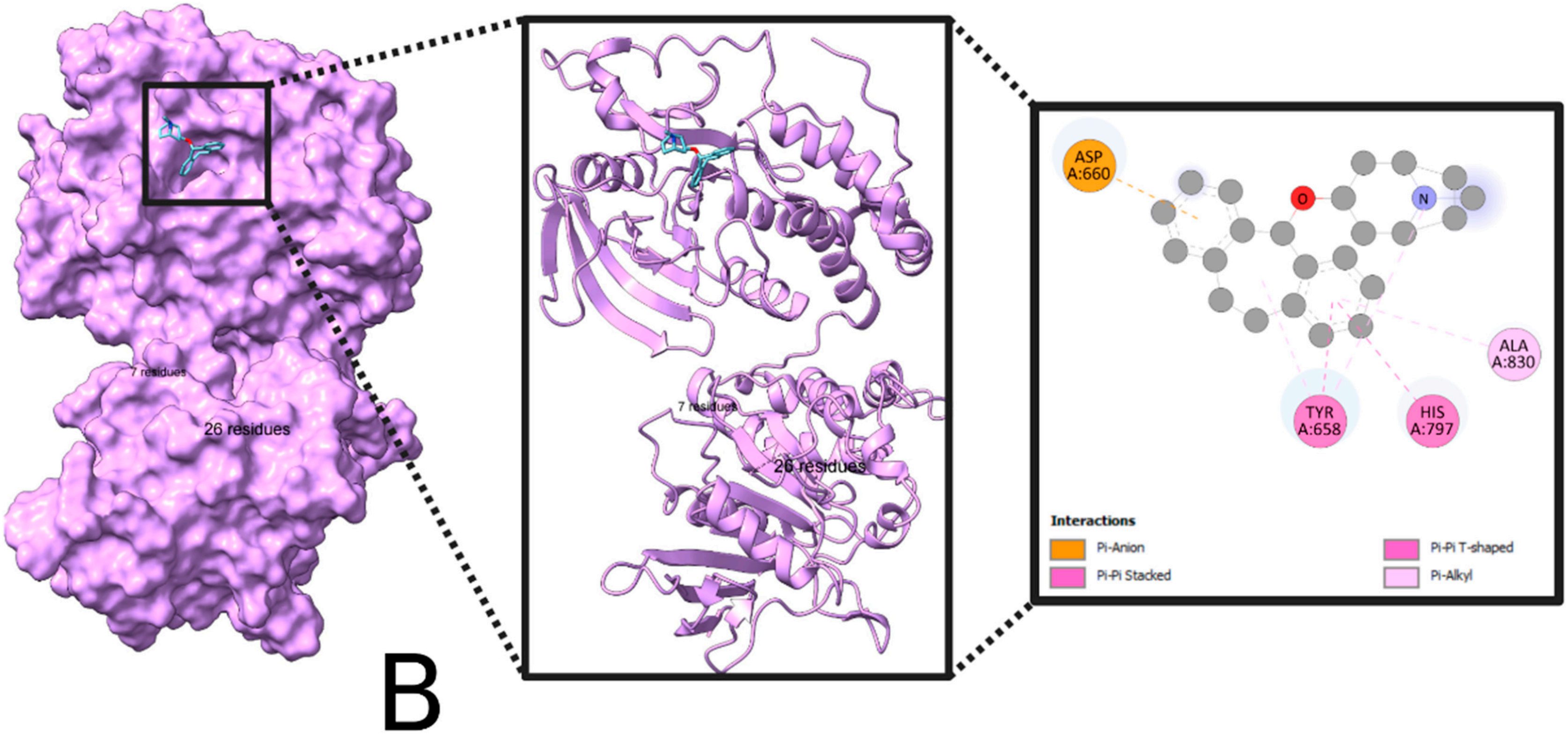

Figure S9: Molecular interaction of PTPRC with the selected compound (PTPRC + Deptropine)

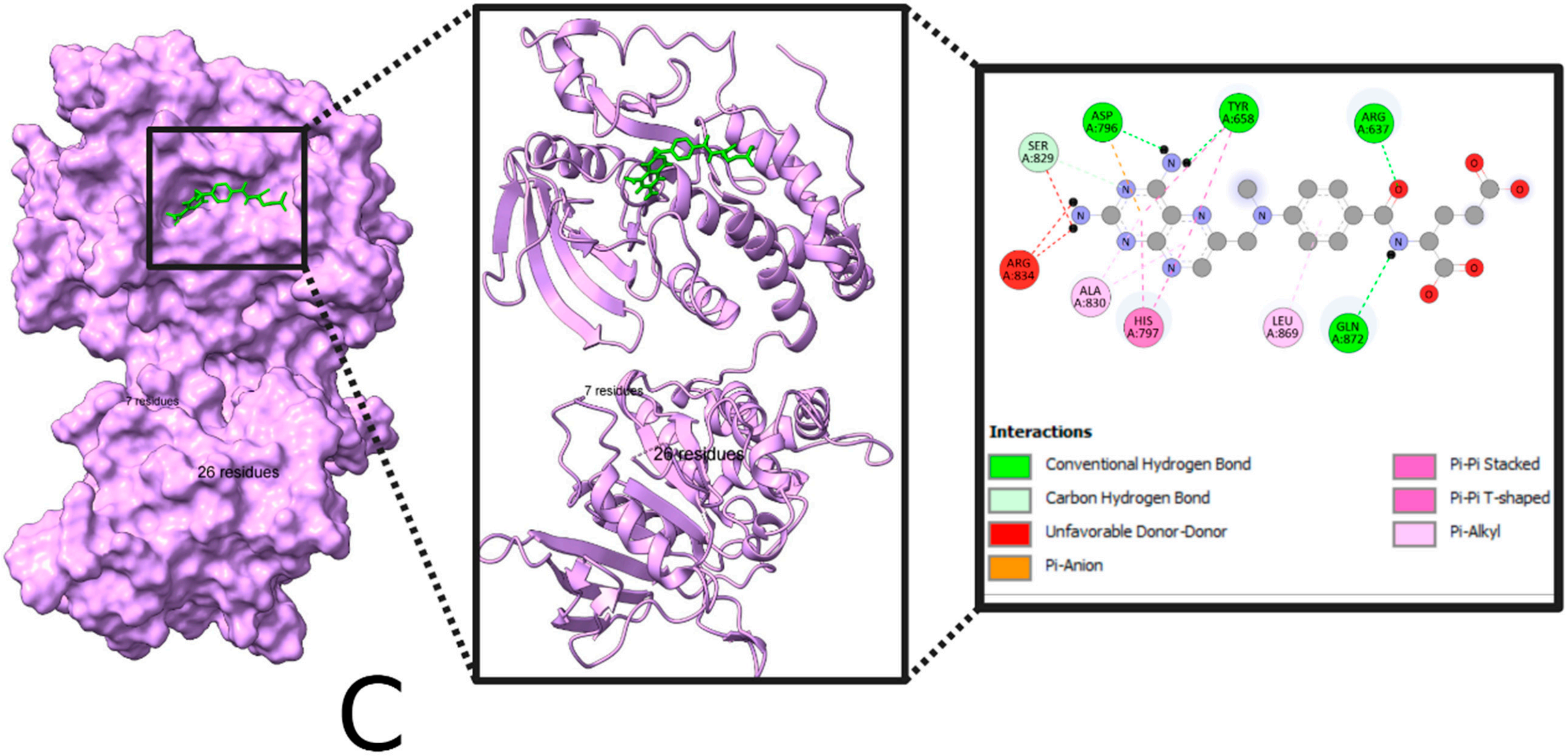

Figure S10: Molecular interaction of PTPRC with the selected compound (PTPRC + Methotrexate)

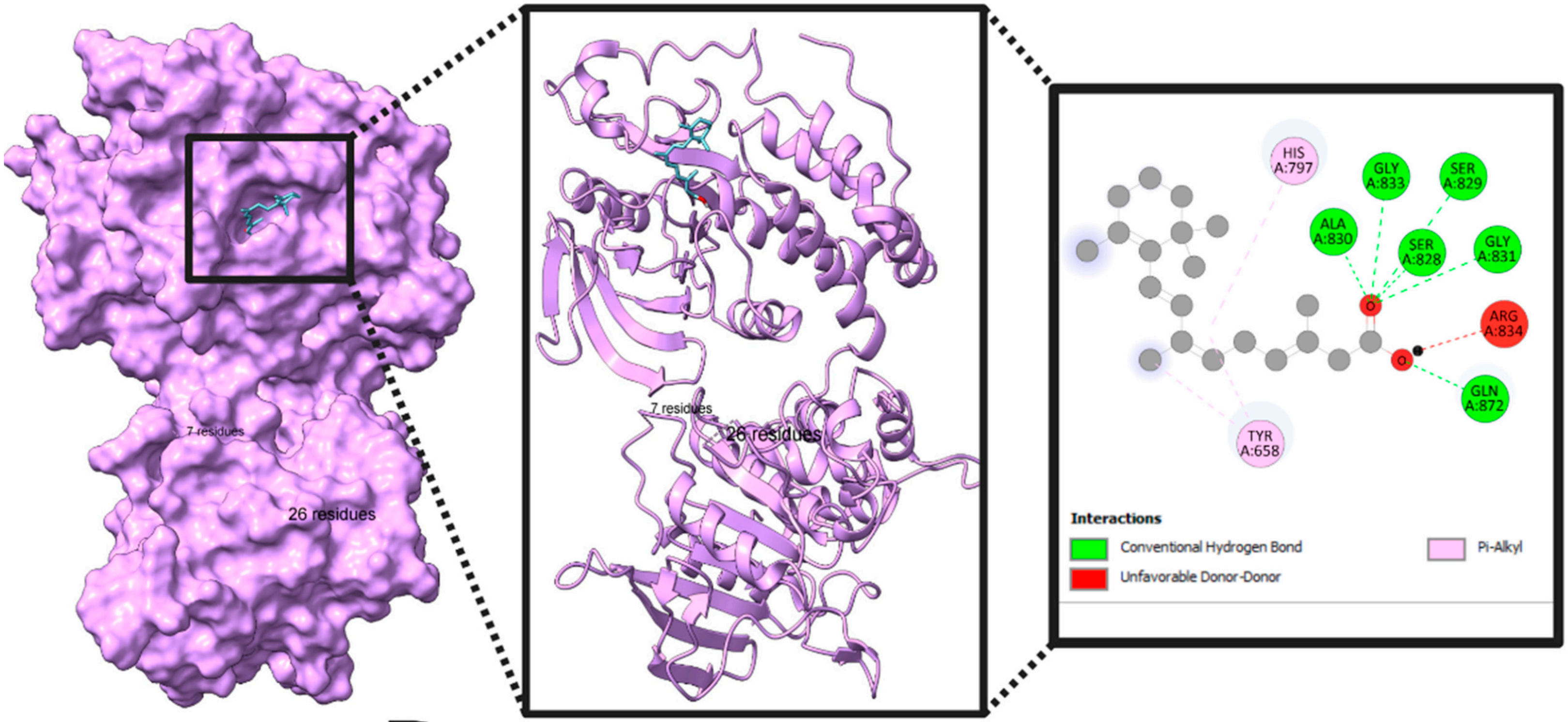

D

Figure S11: Molecular interaction of PTPRC with the selected compound (PTPRC + Retinoic acid)

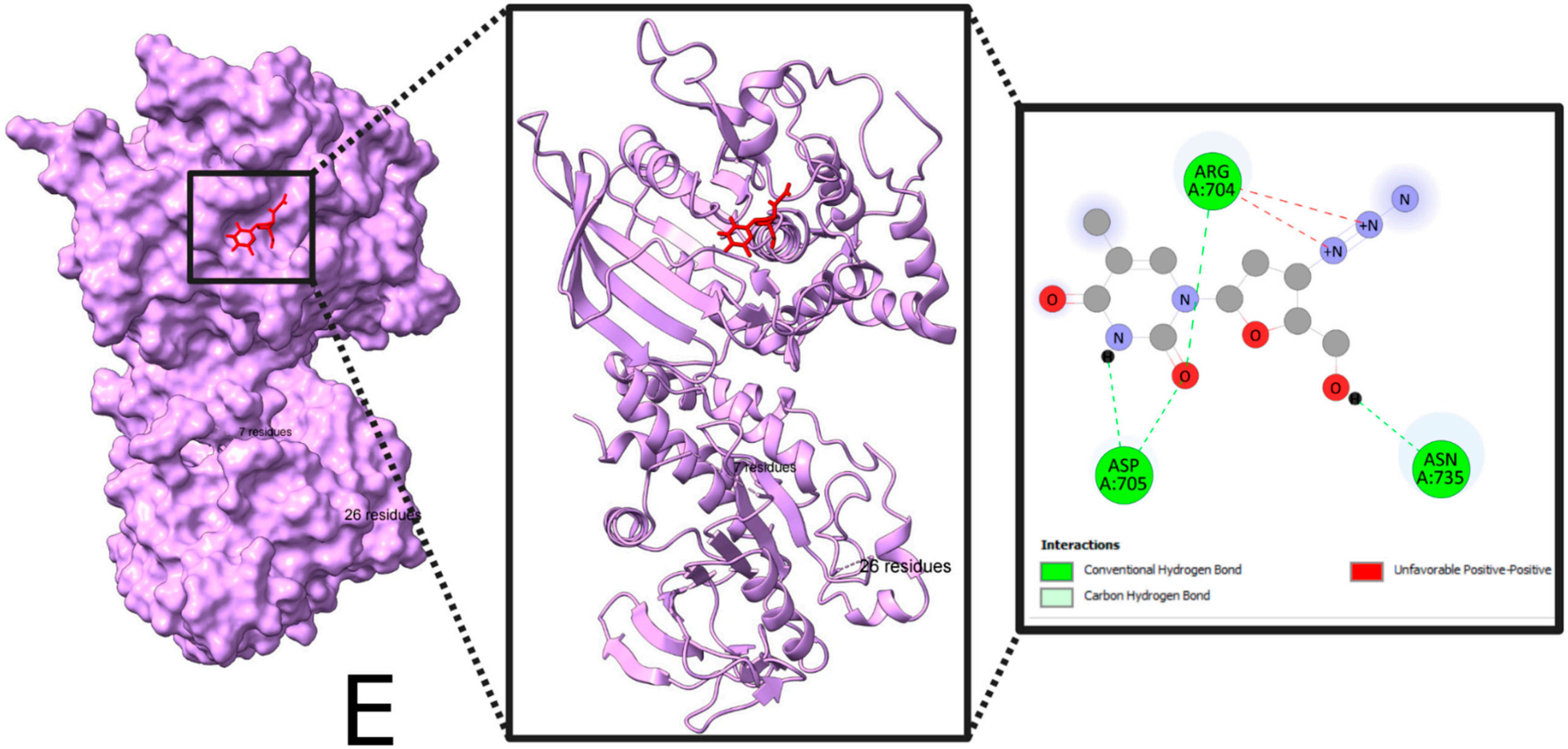

Figure S12: Molecular interaction of PTPRC with the selected compound (PTPRC + 3-Azido-3-deoxythymidine)

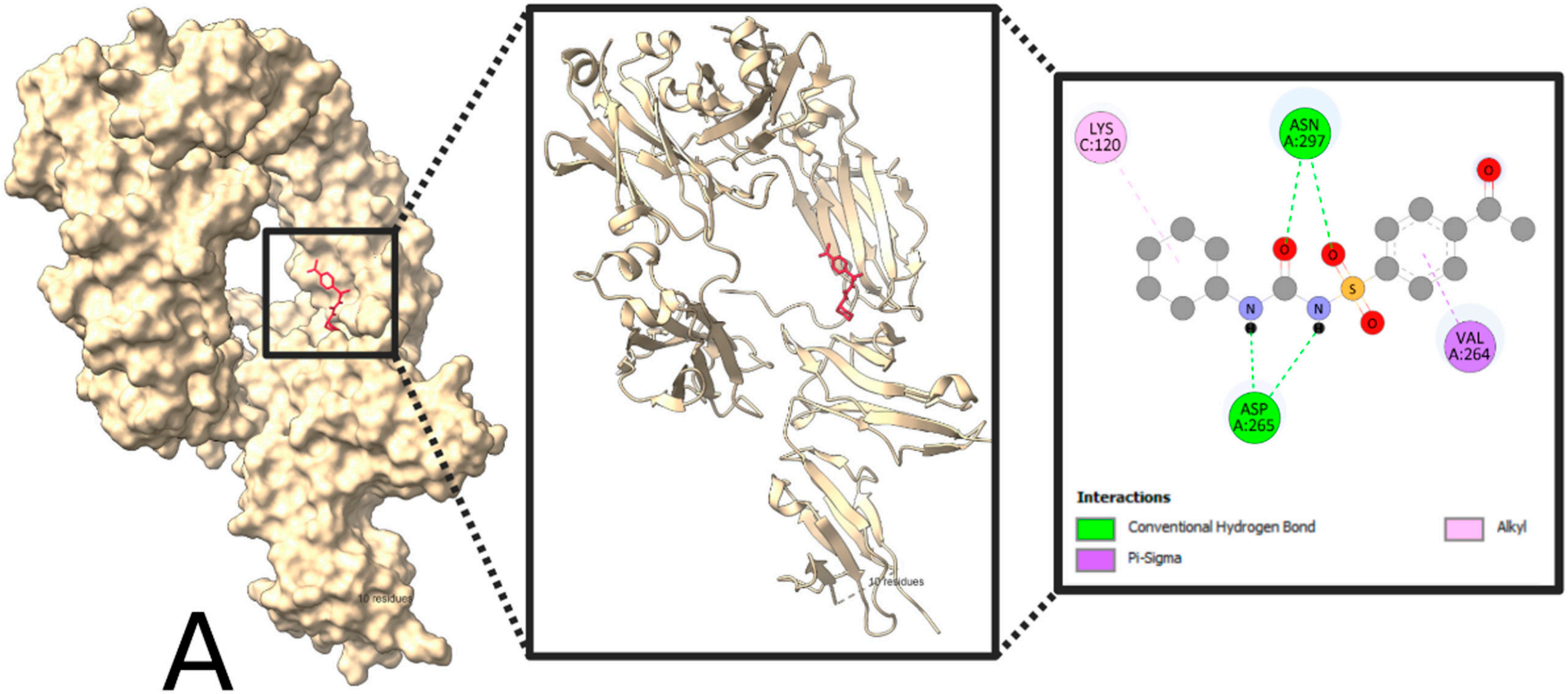

Figure S13: Molecular interaction of FCGR3A with the selected compound (FCGR3A + Acetohexamide)

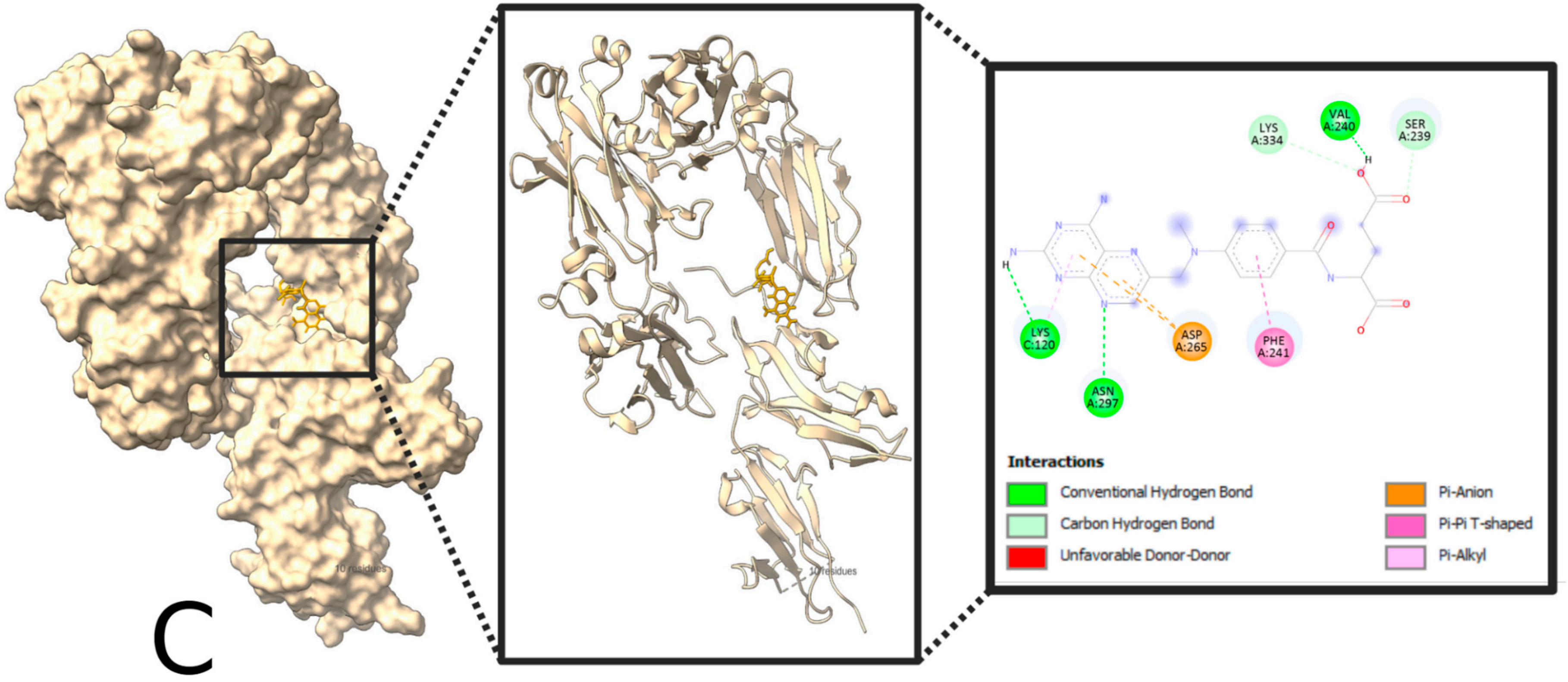

Figure S14: Molecular interaction of FCGR3A with the selected compound (FCGR3A + Methotrexate)

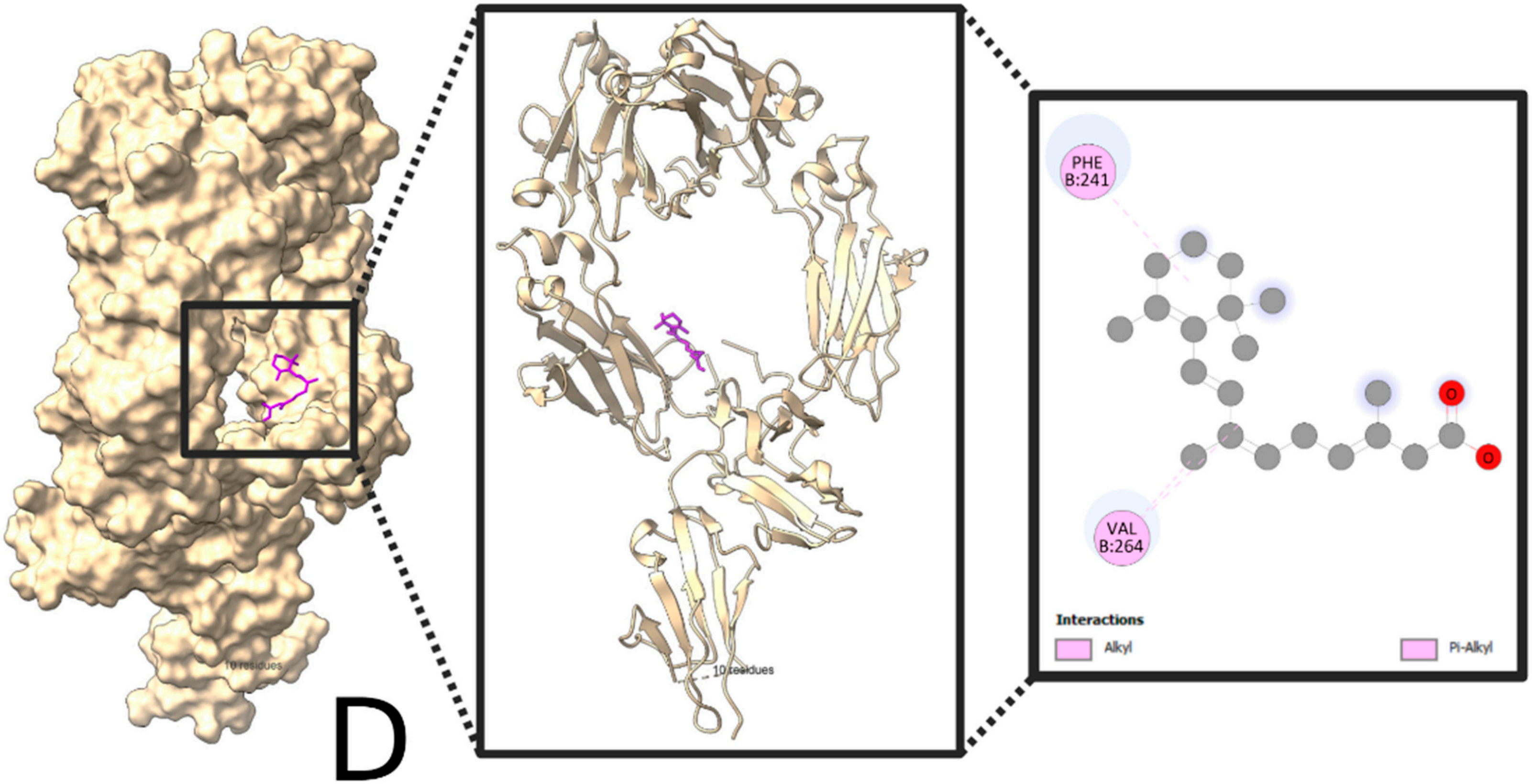

Figure S15: Molecular interaction of FCGR3A with the selected compound (FCGR3A + Retinoic acid)

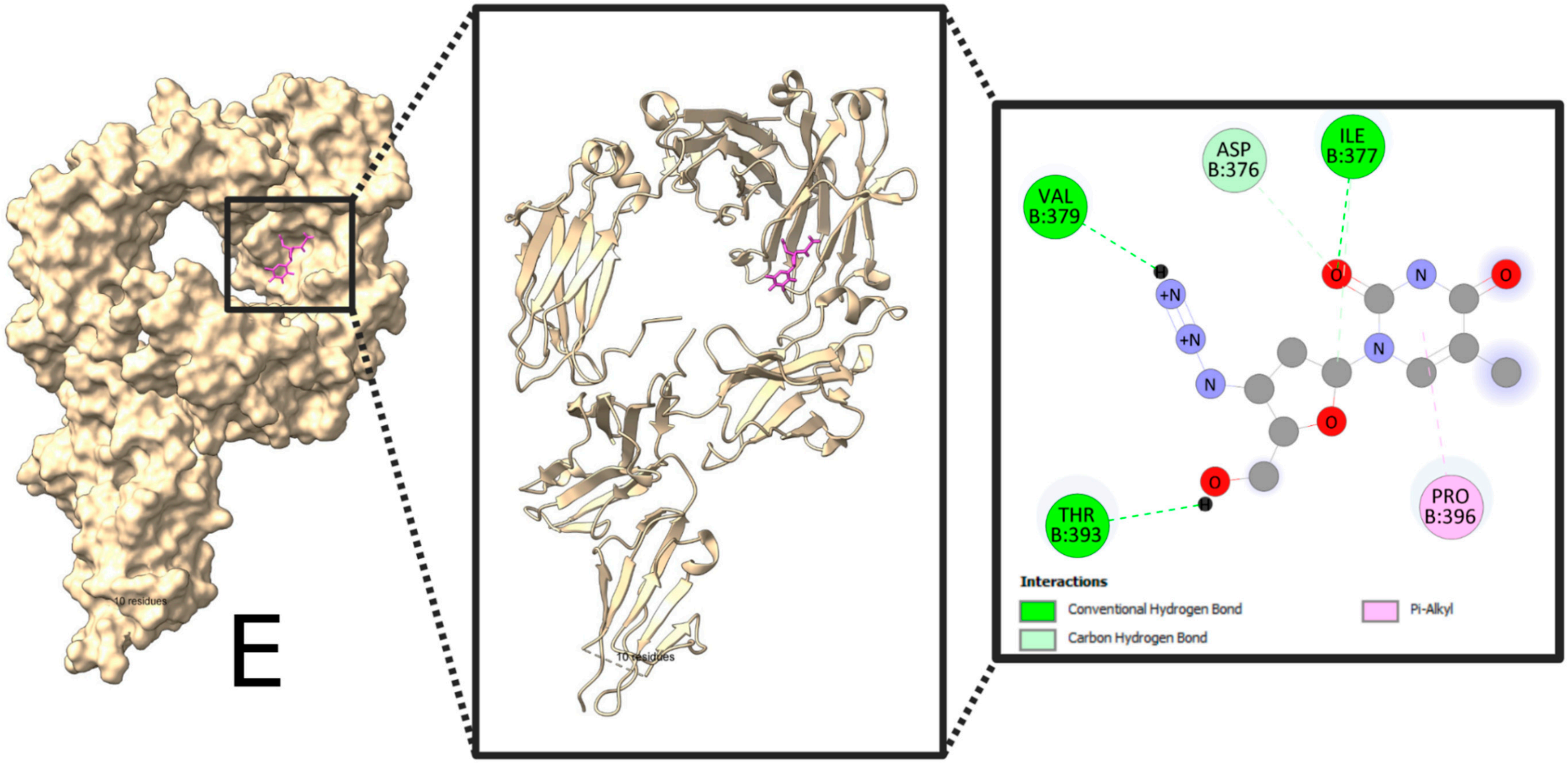

Figure S16: Molecular interaction of FCGR3A with the selected compound (FCGR3A + 3-Azido-3-deoxythymidine)
